# SR‐A3 Promotes USP18‐Mediated deISGylation of STING to Protect Against Myocardial Ischemia‐Reperfusion Injury

**DOI:** 10.1002/advs.77196

**Published:** 2026-08-17

**Authors:** Yufei Han, Guolin Miao, Yinqi Zhao, Zhiyong Du, Kaikai Lu, Changqi Xiao, Aiden Xian, Jiaxin Duanmu, Zihao Zhou, Jinxuan Chen, Lianxin Zhang, Chao Zhai, Yitong Xu, Zhaoling Li, Xianqin Shen, Sijing Shi, Jingxuan Chen, Si Mei, Yuan Fang, Liwen Zheng, Ling Zhang, Yuhui Wang, Wei Huang, Erdan Dong, Xunde Xian

**Affiliations:** ^1^ Institute of Cardiovascular Sciences State Key Laboratory of Vascular Homeostasis and Remodeling School of Basic Medical Sciences Peking University Beijing China; ^2^ Department of Cardiology and Institute of Vascular Medicine Peking University Third Hospital Beijing China; ^3^ National Clinical Research Center for Cardiovascular Diseases Beijing Institute of Heart Lung and Blood Vessel Disease Beijing Anzhen Hospital Capital Medical University Beijing China; ^4^ Shepton High School Plano Texas USA; ^5^ Department of Cardiology Peking University First Hospital Beijing China; ^6^ Research Center for Cardiopulmonary Rehabilitation University of Health and Rehabilitation Sciences Qingdao Hospital (Qingdao Municipal Hospital) School of Health and Life Sciences University of Health and Rehabilitation Sciences Qingdao China; ^7^ Research Unit of Medical Science Research Management/Basic and Clinical Research of Metabolic Cardiovascular Diseases Chinese Academy of Medical Sciences Beijing China; ^8^ Department of Human Anatomy and Histology and Embryology School of Basic Medical Sciences Peking University Beijing China; ^9^ Beijing Key Laboratory of Cardiovascular Receptors Research Peking University Third Hospital Beijing China

**Keywords:** interferon‐stimulated gene 15, myocardial ischemia/reperfusion, scavenger receptor class A member 3, stimulator of interferon response CGAMP interactor, ubiquitin‐specific peptidase 18

## Abstract

Acute myocardial infarction (MI) is a leading cause of mortality worldwide. Although reperfusion restores coronary blood flow, it paradoxically induces secondary injury that limits therapeutic benefits, and effective interventions for myocardial ischemia/reperfusion injury (MI/RI) remain lacking. Here, we identify scavenger receptor class A member 3 (SR‐A3) as a previously unrecognized cardioprotective factor that plays a central regulatory role in ischemic injury. SR‐A3 was significantly downregulated in myocardial tissues from MI/RI patients. Global or cardiomyocyte‐specific deletion of SR‐A3 exacerbated MI/RI‐induced cardiac damage, whereas AAV9‐mediated overexpression alleviated myocardial injury and improved cardiac function. Notably, SR‐A3 expression in cardiomyocytes underwent the most dramatic reduction upon I/R stress, underscoring its critical role in the injured myocardium. Mechanistically, SR‐A3 interacts with ubiquitin‐specific peptidase 18 (USP18) to suppress interferon‐stimulated gene 15 (ISG15)‐dependent ISGylation, thereby negatively regulating activation of the STING signaling pathway and its downstream innate immune inflammatory cascades. Treatment with Selonsertib, an ASK1 inhibitor that increased USP18 abundance in our MI/RI model, recapitulated the cardioprotective effects, highlighting the translational potential of targeting this signaling axis. Collectively, our findings uncover a novel SR‐A3/USP18/ISG15/STING regulatory pathway governing innate immune activation in MI/RI and position SR‐A3 as a promising therapeutic target for ischemic heart disease.

## Graphical abstract

MI/RI is a major clinical challenge with high morbidity and mortality. This study demonstrates that SR‐A3 expression is downregulated in MI/RI, and its deletion exacerbates damage via the USP18/ISG15/STING axis, revealing SR‐A3 as a potential therapeutic target. Importantly, Selonsertib, a Phase III clinical drug, upregulates USP18 to inhibit this pathway, offering a promising strategy to mitigate MI/RI and improve cardiovascular outcomes.

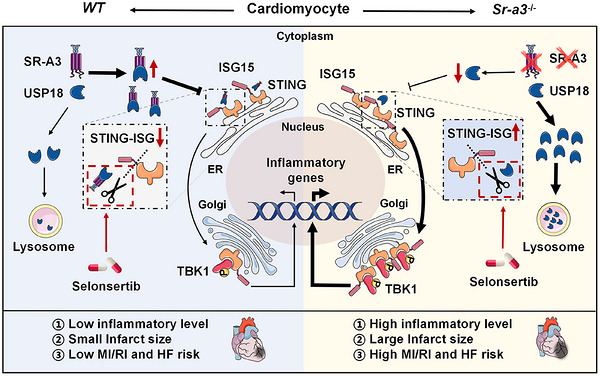

**Abbreviations**
AAV9: Adeno‐associated virus 9
CK: Creatine kinase
H/R: Hypoxia/reoxygenation
IHD: Ischemic heart disease
ISG15: Interferon‐stimulated gene 15
LDH: Lactate dehydrogenase
MI: Myocardial infarction
MI/RI: Myocardial ischemia‐reperfusion injury
NRCMs: Neonatal rat cardiomyocytes
PCI: Percutaneous coronary intervention
ROS: Reactive oxygen species
SR‐A3: Scavenger receptor class A3
STING: Stimulator of interferon response cGAMP interactor
TUNEL: Terminal deoxynucleotidyl transferase‐mediated dUTP Nick‐end labeling
USP18: Ubiquitin specific peptidase 18

## 1 Introduction

Acute myocardial infarction (MI) is one of the most severe and life‐threatening clinical manifestations of cardiovascular disease and remains a major contributor to global morbidity and mortality, and timely reperfusion to restore coronary blood flow through primary percutaneous coronary intervention (PCI), coronary artery bypass graft, or thrombolysis is the primary treatment for MI [1, 2]. However, reperfusion of oxygen paradoxically further exacerbates irreversible tissue damage through mechanisms involving oxidative stress, calcium overload, and inflammatory cascades, leading to myocardial injury, which is termed myocardial ischemia/reperfusion injury (MI/RI), a major cause of adverse outcomes of revascularization after MI [3, 4]. Increasing lines of evidence highlight the role of damage‐associated molecular patterns (DAMPs), such as extracellular RIPK3, which elicit innate immune pathways via interacting with RAGE to activate CaMKII, amplifying myocardial inflammation and apoptosis [5]. Despite progress in understanding these mechanisms, the absence of effective clinical therapies to mitigate MI/RI emphasizes the urgent need to identify new therapeutic molecules and then target this process in ischemic heart disease treatment.

The scavenger receptor class A (SR‐A) family has been traditionally recognized for their dual roles in lipid metabolism and innate immunity [6]. Prior studies have highlighted the emerging significance of SR‐A1, the first identified member of SR‐A family, as a pivotal contributor to cardiovascular pathologies [7]. Notably, certain SR‐A members are implicated in sensing damage‐associated molecular patterns (DAMPs), thus bridging innate immune activation to tissue injury [8]. Unfortunately, in the context of MI/RI, the relationship between SR‐A receptors, DAMPs, and myocardial injury progression has not been established yet.

Unlike other SR‐A family members that are highly expressed at the cell surface plasma membrane, SR‐A3 lacks the C‐terminal globular binding domain of scavenger receptor cysteine‐rich (SRCR), which mainly localizes at the endoplasmic reticulum (ER) and Golgi apparatus [6]. Recently, SR‐A3 has been reported to execute a protective function against cellular damage caused by UV irradiation and oxidative stress [9, 10]. Additionally, loss of hepatic SR‐A3 predisposes multiple small rodent animal models to liver lipid accumulation, inflammation, and fibrosis by activating the XIAP/PTEN/AKT axis [11]. These findings demonstrate a crucial role of SR‐A3 in maintaining cellular homeostasis under metabolic stress and its potential as a therapeutic target in metabolic disease. However, to date, its specific involvement in MI/RI remains unknown, warranting further investigation into its cardioprotective potential.

Here, we found that genetic ablation of SR‐A3 exacerbated cardiac damage, manifesting as enlarged infarct areas and heightened cardiomyocyte inflammation, whereas systemic or cardiomyocyte‐specific SR‐A3 overexpression mediated by adeno‐associated virus 9 (AAV9) conferred dual preventive and therapeutic benefits against MI/RI and subsequent heart failure. Mechanistically, SR‐A3 attenuates MI/RI by interacting with ubiquitin‐specific peptidase 18 (USP18), thereby suppressing ISG15‐dependent ISGylation and activation of the stimulator of interferon response cGAMP interactor (STING). Importantly, we demonstrated that Selonsertib, a clinically available chemotherapeutic agent, exerts cardioprotective effects by upregulating USP18 expression to ameliorate MI/RI. This study elucidates the fundamental role of SR‐A3 in MI/RI and addresses the critical gaps in understanding its cardioprotective functions and the potential therapeutic strategies of treating this devastating disease.

## 2 Results

### 2.1 Decreased Expression of SR‐A3 in Heart Tissues is Linked to MI/RI in both Humans and Mice

**FIGURE 1:**
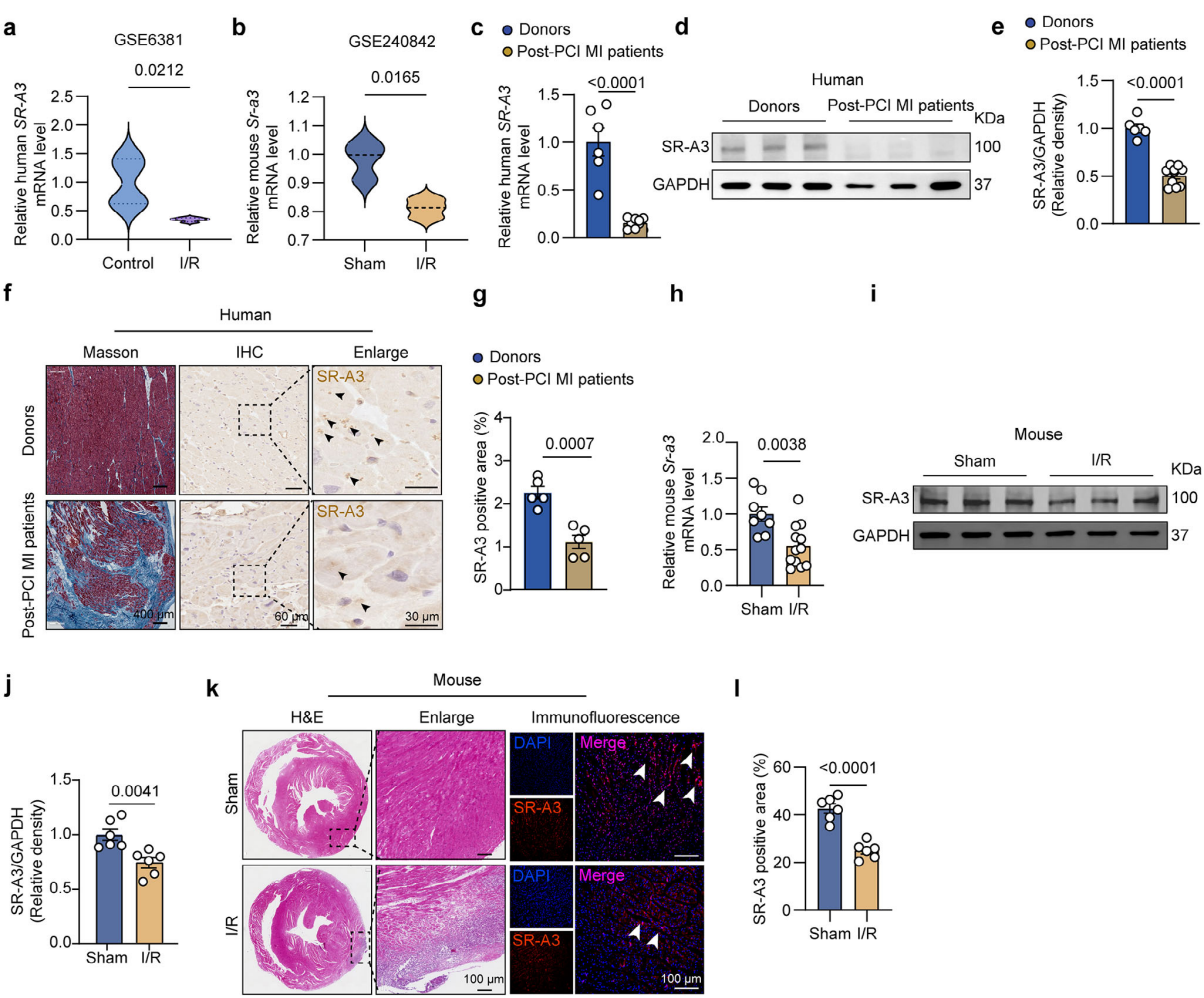
SR‐A3 is downregulated during myocardial ischemia/reperfusion injury. (a) Transcriptional expression of *SR‐A3* was assessed using RNA‐sequencing data (GSE6381) from human heart samples, including controls (before ischemia, *n* = 4) and patients undergoing ischemia‐reperfusion (after 5 min of reperfusion, *n* = 4). (b) Transcriptional expression of *Sr‐a3* was evaluated in RNA‐sequencing data (GSE240842) from mice, comparing sham‐operated controls (*n* = 4) and mice subjected to myocardial ischemia/reperfusion injury (MI/RI, *n* = 4). (c) QPCR analysis of *SR‐A3* mRNA levels in heart tissues from donors (*n* = 6) and post‐percutaneous coronary intervention (PCI) myocardial infarction (MI) patients (*n* = 10). (d) Western blot analysis of SR‐A3 protein expression in heart tissues from donors (*n* = 6) and post‐PCI MI patients (*n* = 10). (e) Quantification of the data shown in (d). (f) Representative images of Masson's trichrome and immunohistochemical staining for SR‐A3 in heart tissues from a donor and a post‐PCI MI patient. (g) Quantitative analysis of SR‐A3 positive staining area and intensity. (h) QPCR analysis of *Sr‐a3* mRNA levels in heart tissues from sham‐operated controls (*n* = 8) and mice subjected to MI/RI (*n* = 12). (i) Western blot analysis of SR‐A3 protein levels in mouse hearts after sham or MI/RI. (j) Quantification of the data shown in (i). (k) Representative images of H&E and immunofluorescence staining for SR‐A3 in heart tissues from sham‐operated controls and MI/RI mice. (l) Quantification of the immunofluorescence data shown in (k). Black arrowheads indicate SR‐A3‐positive staining in heart tissues from a normal control and a post‐PCI patient; white arrowheads indicate SR‐A3‐positive staining in heart tissues from sham‐operated controls and MI/RI mice. Data are presented as the mean ± SEM and were analyzed by two‐tailed unpaired Student's *t*‐test.

To clarify the potential role of SR‐A3 in MI/RI, we first measured *SR‐A3* mRNA expression levels in the heart in both humans and mice with MI/RI using the GEO public database, in which a significant reduction in *SR‐A3* mRNA levels was observed in the heart tissues following MI/RI (Figure 1a,b). Next, we examined SR‐A3 expression in the heart tissues collected from patients undergoing percutaneous coronary intervention (PCI), both mRNA and protein levels of *SR‐A3* were markedly lower than those in control heart tissues (Figure 1c–g). Consistently, in a mouse MI/RI model, qPCR, Western blotting, and immunofluorescence staining showed that both *Sr‐a3* mRNA and protein expression levels were significantly decreased after MI/RI compared with the sham‐operated group (Figure 1h–l). Together, these results establish a negative correlation between SR‐A3 expression level in heart tissues and the damage of MI/RI.

### 2.2 Depletion of Endogenous *Sr‐a3* Exacerbated MI/RI‐Induced Cardiac Injury and Remodeling in Mice

**FIGURE 2:**
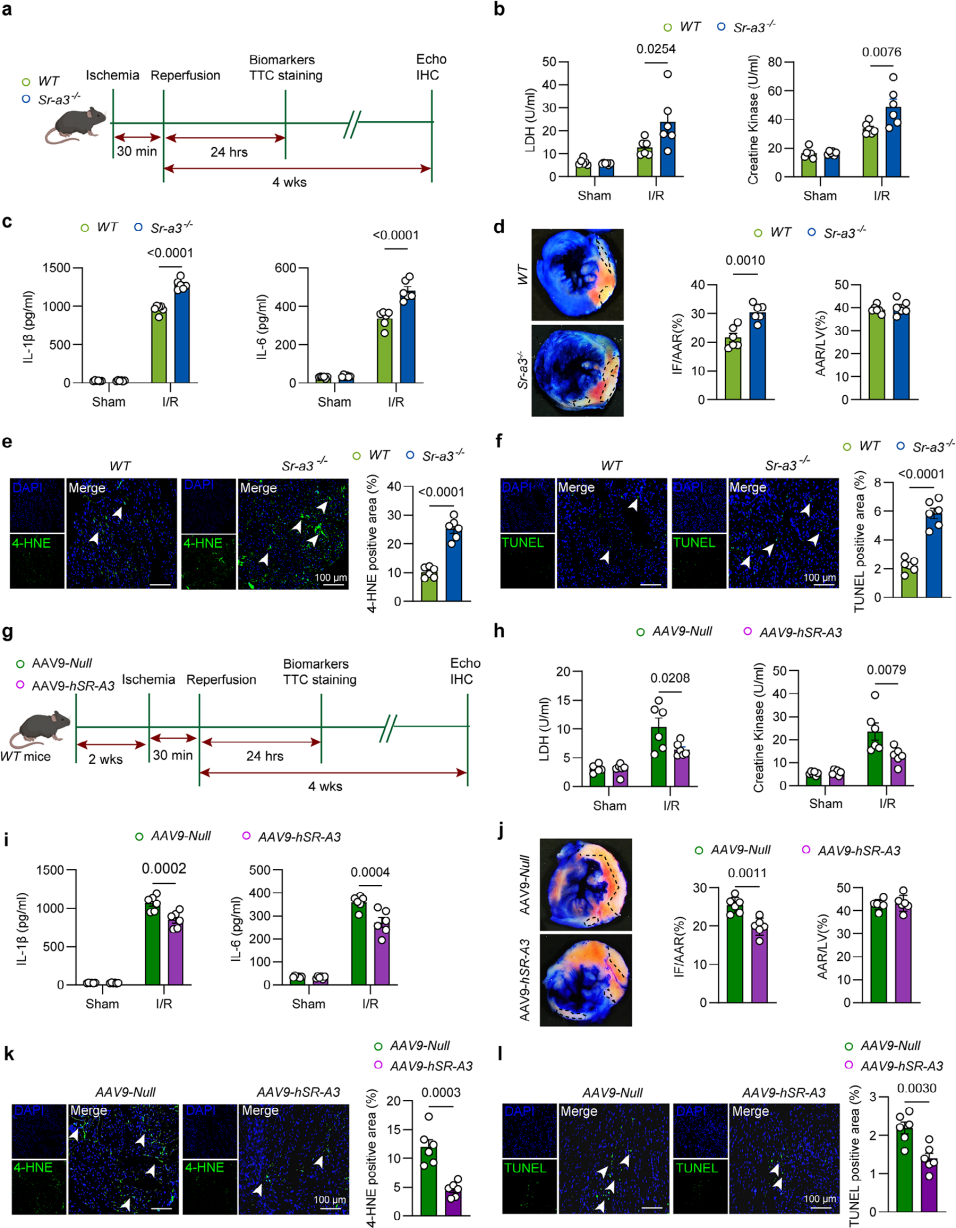
Global knockout of *Sr‐a3* exacerbates myocardial ischemia/reperfusion injury. (a) Schematic diagram of the experimental protocol for panels (b) to (f). (b) Plasma levels of lactate dehydrogenase (LDH) and creatine kinase (CK) in wild‐type (*WT*) and *Sr‐a3^−/−^* mice, with or without MI/RI (30‐min ischemia followed by 24‐h reperfusion; *n* = 6 per group). (c) Plasma levels of pro‐inflammatory cytokines (IL‐1β, IL‐6) in *WT* and *Sr‐a3^−/−^* mice, with or without MI/RI (*n* = 6 per group). (d) Representative Evans blue/TTC‐stained cardiac tissue sections and quantification of infarct size in *WT* and *Sr‐a3^−/−^* mice subjected to MI/RI. The non‐ischemic area is stained blue, the area at risk (AAR) red, and the infarct area (IF) appears white (outlined in black dash lines; *n* = 6 per group). (e) Representative myocardial sections stained for 4‐hydroxynonenal (4‐HNE; scale bar: 100 µm) and quantification of the positively stained area in *WT* and *Sr‐a3^−/−^* mice after MI/RI (*n* = 6 per group). 4‐HNE‐positive cells are labeled green, and nuclei are counterstained with DAPI (blue). White arrowheads highlight 4‐HNE‐positive staining in mouse heart tissues. (f) Representative TUNEL‐stained myocardial sections (scale bar: 100 µm) and quantification of the TUNEL‐positive area in *WT* and *Sr‐a3^−/−^* mice after MI/RI (*n* = 6 per group). TUNEL‐positive cells are labeled green, and nuclei are counterstained with DAPI (blue). White arrowheads highlight TUNEL‐positive staining in mouse heart tissues. (g) Schematic diagram of the experimental protocol for panels (h–l). (h) Plasma LDH and CK levels in mice infected with AAV*9‐Null* or AAV9*‐hSR‐A3*, with or without MI/RI (*n* = 6 per group). (i) Plasma levels of IL‐1β and IL‐6 in mice infected with AAV*9‐Null* or AAV9*‐hSR‐A3*, with or without MI/RI (n = 6 per group). (j) Representative Evans blue/TTC staining of cardiac sections and infarct size quantification in mice infected with AAV*9‐Null* or AAV9*‐hSR‐A3* after MI/RI (*n* = 6 per group). The non‐ischemic area (blue), AAR (red), and IF (white, outlined in black dash lines) are indicated. (k) Representative 4‐HNE‐stained myocardial sections (scale bar: 100 µm) and quantitative analysis of 4‐HNE‐positive area in the heart of mice infected with AAV9*‐Null* or AAV9*‐hSR‐A3* after MI/RI (*n* = 6 per group). 4‐HNE‐positive cells are labeled green, and nuclei are counterstained with DAPI (blue). White arrowheads highlight 4‐HNE‐positive staining in mouse heart tissues. (l) Representative TUNEL staining of myocardial sections (scale bar: 100 µm) and quantification of the TUNEL‐positive area in the heart of mice infected with AAV9*‐Null* or AAV9*‐hSR‐A3* after MI/RI (*n* = 6 per group). TUNEL‐positive cells are labeled green, and nuclei are counterstained with DAPI (blue). White arrowheads highlight TUNEL‐positive staining in mouse heart tissues. Data are presented as the mean ± SEM and were analyzed by two‐tailed unpaired Student's *t*‐test or two‐way ANOVA with Tukey post hoc tests.

To investigate the influence of SR‐A3 in MI/RI, we generated global *Sr‐a3* knockout (*Sr‐a3* ^−/−^) mice, and SR‐A3 deficiency was validated by quantitative PCR (qPCR) and genotyping (Figure S1). Subsequently, hearts from *Sr‐a3* ^−/−^ mice and their littermate *wild‐type* (*WT*) controls were subjected to acute injury with 30 min ischemia followed by 24 h reperfusion (Figure 2a). The extent of cardiomyocyte death, assessed by serum levels of lactate dehydrogenase (LDH) and creatine kinase (CK), was aggravated in *Sr‐a3* ^−/−^ mice (Figure 2b). Similarly, the inflammatory response, reflected by serum interleukin‐1β (IL‐1β) and IL‐6 concentrations, was also enhanced (Figure 2c). Furthermore, infarct size after MI/RI was significantly larger in *Sr‐a3* ^−/−^ hearts compared with *WT* hearts (Figure 2d). Given that SR‐A3 is a known regulator of oxidative stress and apoptosis, we evaluated reactive oxygen species (ROS) production and apoptosis in heart tissues by immunofluorescence staining for 4‐hydroxynonenal (4‐HNE) and terminal deoxynucleotidyl transferase‐mediated dUTP nick‐end labeling (TUNEL). Indeed, MI/RI‐induced oxidative stress and apoptosis were markedly intensified in the absence of SR‐A3 (Figure 2e,f).

Although timely reperfusion and adjunctive pharmacotherapies have substantially reduced acute mortality following myocardial infarction, the consequent decline in mortality has been accompanied by a rising incidence of chronic cardiac remodeling and heart failure. Therefore, we next examined the involvement of SR‐A3 in maladaptive cardiac remodeling and dysfunction using a chronic model of 30 min ischemia followed by 4 weeks of reperfusion. Echocardiographic analysis revealed that *Sr‐a3* ^−/−^ mice exhibited impaired cardiac function, as indicated by reduced ejection fraction (EF) and fractional shortening (FS), together with increased left‐ventricular end‐diastolic and end‐systolic dimensions (LVIDd and LVIDs), whereas left‐ventricular posterior‐wall dimensions (LVPWD) and systolic thickness (LVPWS) remained unchanged (Figure S2a). These functional alterations were accompanied by prominent cardiac chamber enlargement and hypertrophy in *Sr‐a3* ^−/−^ mice (Figure S2b). Consistent with these observations, the ratios of heart weight to body weight and heart weight to tibia length were elevated in *Sr‐a3* ^−/−^ mice compared with *WT* controls (Figure S2c). Pathological analyses further demonstrated a significant increase in cardiomyocyte cross‐sectional area stained with Wheat Germ Agglutinin (WGA), enhanced 4‐HNE accumulation, and elevated cardiomyocyte apoptosis in *Sr‐a3* ^−/−^ hearts (Figure S2d,e).

To further delineate the beneficial effect of SR‐A3 on both acute MI/RI and chronic remodeling, *WT* mice were intravenously injected with adeno‐associated virus serotype 9 (AAV9) expressing human *SR‐A3* (*hSR‐A3*) and then subjected to acute MI/RI or the chronic remodeling protocol (Figure 2g). Overexpression of *hSR‐A3* significantly attenuated MI/RI‐induced cardiomyocyte death with decreased serum LDH and CK levels (Figure 2h), and also mitigated the inflammatory response, reflected by lower serum IL‐1β and IL‐6 concentrations (Figure 2i). Moreover, infarct size after MI/RI was markedly reduced in hearts overexpressing *hSR‐A3* (Figure 2j). Correspondingly, hSR‐A3 overexpression substantially alleviated MI/RI‐triggered oxidative stress and apoptosis in cardiomyocytes (Figure 2k,l).

In the chronic remodeling model, echocardiography showed that mice with *hSR‐A3* overexpression displayed improved cardiac function with increased EF and FS as well as reduced LVIDd and LVIDs, while LVPWD and LVPWS remained unaltered (Figure S2f). This functional improvement was associated with attenuated cardiac chamber enlargement and hypertrophy (Figure S2g). Consistently, the ratios of heart weight to body weight and heart weight to tibia length were decreased in *hSR‐A3* overexpressing mice relative to control animals administered AAV9‐*Null* (Figure S2h). Pathological analyses further revealed that *hSR‐A3* overexpression led to smaller cardiomyocyte size, diminished 4‐HNE accumulation, and reduced cardiomyocyte apoptosis (Figure S2i,j).

Taken together, these findings demonstrate that SR‐A3 plays a critical protective role in MI/RI by attenuating cardiomyocyte death, oxidative stress, inflammation, and adverse remodeling, while its deficiency exacerbates these pathological processes.

### 2.3 Cardiomyocyte‐Derived SR‐A3 is Required for Protection Against Ischemia/Reperfusion‐Induced Cardiac Injury and Remodeling

**FIGURE 3:**
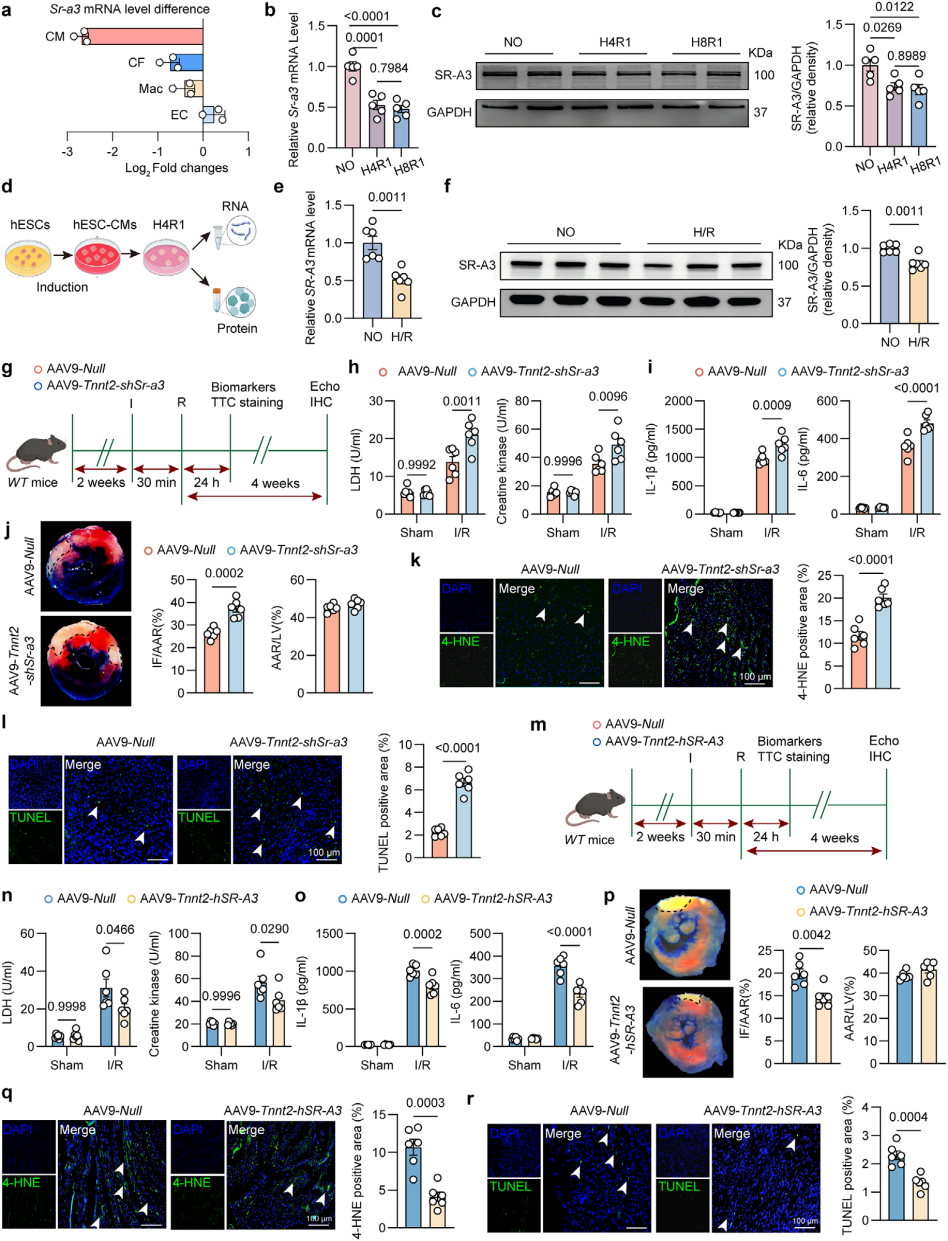
Cardiac‐specific knockdown or overexpression of *Sr‐a3* modulates myocardial ischemia/reperfusion injury. (a) QPCR analysis of *Sr‐a3* mRNA expression (log2 fold changes) in adult mouse cardiomyocytes (CM), cardiac fibroblasts (CF), macrophages (Mac, F4/80+), and endothelial cells (EC, CD31+) after MI/RI (30‐min ischemia followed by 24‐h reperfusion; *n* = 3 per group). (b) QPCR analysis of *Sr‐a3* expression in neonatal rat cardiomyocytes (NRCMs) subjected to normal oxygen (NO) or hypoxia/reoxygenation (H/R) (4 h hypoxia/1 h reoxygenation (H4/R1) or 8 h hypoxia/1 h reoxygenation (H8/R1); *n* = 5 per group, independent biological replicates). (c) Western blot analysis of SR‐A3 protein expression and corresponding quantification in NRCMs after H/R (*n* = 5 per group, independent biological replicates). (d) Schematic diagram of the experimental protocol for panels (e, f). (e) QPCR analysis of *SR‐A3* expression in human embryonic stem cell‐derived cardiomyocytes (hESC‐CMs) after H/R (4 h hypoxia/1 h reoxygenation; *n* = 6 per group, independent biological replicates). (f) Western blot analysis of SR‐A3 protein expression and quantification in hESC‐CMs after H/R (*n* = 6 per group, independent biological replicates). (g) Schematic diagram of the experimental protocol for panels (h–l). (h) Plasma LDH and CK levels in mice infected with AAV9*‐Null* or AAV9*‐Tnnt2‐shSr‐a3* (cardiac‐specific knockdown), with or without MI/RI (*n* = 6 per group). (i) Plasma levels of IL‐1β and IL‐6 in AAV9*‐Null* or AAV9*‐Tnnt2‐shSr‐a3* infected mice, with or without MI/RI (*n* = 6 per group). (j) Representative Evans blue/TTC‐stained cardiac sections and infarct size quantification in AAV9*‐Null* or AAV9*‐Tnnt2‐shSr‐a3* infected mice subjected to MI/RI (*n* = 6 per group). (k) Representative 4‐HNE‐stained myocardial sections (scale bar: 100 µm) and quantitative analysis in AAV9*‐Null* or AAV9*‐Tnnt2‐shSr‐a3* infected mice subjected to MI/RI (*n* = 6 per group). 4‐HNE‐positive cells are labeled green, and nuclei are counterstained with DAPI (blue). White arrowheads highlight 4‐HNE‐positive staining in mouse heart tissues. (l) Representative TUNEL‐stained myocardial sections (scale bar: 100 µm) and quantification of the TUNEL‐positive area in AAV9*‐Null* or AAV9*‐Tnnt2‐shSr‐a3* infected mice subjected to MI/RI (*n* = 6 per group). TUNEL‐positive cells are labeled green, and nuclei are counterstained with DAPI (blue). White arrowheads highlight TUNEL‐positive staining in mouse heart tissues. (m) Schematic diagram of the experimental protocol for panels (n–r). (n) Plasma LDH and CK levels in AAV9*‐Null* or AAV9*‐Tnnt2‐hSR‐A3* (cardiac‐specific overexpression) infected mice with or without MI/RI (*n* = 6 per group). (o) Plasma levels of IL‐1β and IL‐6 in AAV9*‐Null* or AAV9*‐Tnnt2‐hSR‐A3* infected mice with or without MI/RI (*n* = 6 per group). (p) Representative Evans blue/TTC staining and infarct size quantification in AAV9*‐Null* or AAV9*‐Tnnt2‐hSR‐A3* infected mice after MI/RI (*n* = 6 per group). (q) Representative 4‐HNE‐stained myocardial sections (scale bar: 100 µm) and quantitative analysis in AAV9*‐Null* or AAV9*‐Tnnt2‐hSR‐A3* infected mice after MI/RI (*n* = 6 per group). 4‐HNE‐positive cells are labeled green, and nuclei are counterstained with DAPI (blue). White arrowheads highlight 4‐HNE‐positive staining in mouse heart tissues. (r) Representative TUNEL staining of myocardial sections (scale bar: 100 µm) and quantification of the TUNEL‐positive area in AAV9*‐Null* or AAV9*‐Tnnt2‐hSR‐A3* infected mice after MI/RI (*n* = 6 per group). TUNEL‐positive cells are labeled green, and nuclei are counterstained with DAPI (blue). White arrowheads highlight TUNEL‐positive staining in mouse heart tissues. Black dashed lines outline the myocardial infarction area. Data are presented as the mean ± SEM and were analyzed by two‐tailed unpaired Student's *t*‐test or one‐ or two‐way ANOVA with Tukey post hoc tests.

To systematically evaluate SR‐A3 expression across major cardiac cell types, we isolated cardiomyocytes, cardiac fibroblasts, macrophages, and endothelial cells from mouse hearts subjected to MI/RI, and compared their *Sr‐a3* mRNA expression levels by qPCR with values normalized to cardiomyocytes. Upon I/R injury, *Sr‐a3* mRNA expression decreased by over 80% in cardiomyocytes, approximately 40% in fibroblasts, and approximately 25% in macrophages, whereas endothelial cells showed no significant change (Figure 3a). These results indicate that cardiomyocytes exhibit the most pronounced loss of SR‐A3 upon ischemic insult, suggesting a particularly sensitive role for cardiomyocyte‐derived SR‐A3 in response to MI/RI challenge. Consistent with these in vivo findings, hypoxia/reoxygenation (H/R) stimulation of primary neonatal rat cardiomyocytes (NRCMs) led to a marked reduction in SR‐A3 expression (Figure 3b,c). Functionally, knockdown of *Sr‐a3* in NRCMs promoted LDH release and reduced cell viability (Figure S3a–d). Conversely, overexpression of *hSR‐A3* significantly lowered LDH levels and preserved cellular viability under H/R stress (Figure S3e–h). Moreover, similar results were obtained in human embryonic stem cell‐derived cardiomyocytes under H/R conditions, where SR‐A3 expression was markedly downregulated (Figure 3d–f). These data collectively indicate that cardiomyocytes exhibit a more pronounced loss of SR‐A3 upon ischemic insult.

We therefore sought to define the specific contribution of cardiomyocyte‐expressed SR‐A3 by using cardiotropic AAV9 vectors to either knockdown or overexpress SR‐A3 selectively in cardiomyocytes under acute injury conditions (Figure 3g,m). QPCR and Western blot analyses showed that AAV9‐*Tnnt2‐shSr‐a3* markedly reduced *Sr‐a3* mRNA and protein levels in mouse heart tissues, whereas AAV9‐*Tnnt2‐hSR‐A3* significantly increased *SR‐A3* mRNA and protein expression (Figure S3i–l). Immunofluorescence staining further confirmed the cardiomyocyte‐preferential expression pattern of the AAV9‐*Tnnt2* system, as HA‐tagged hSR‐A3 showed strong co‐localization with cTnT‐positive cardiomyocytes but minimal overlap with Vimentin‐positive fibroblasts (Figure S3m). Cardiomyocyte‐specific knockdown of SR‐A3 by AAV9*‐Tnnt2‐shSr‐a3* resulted in elevated serum levels of LDH and CK (Figure 3h), heightened inflammatory markers (IL‐1β and IL‐6) (Figure 3i), enlarged infarct size (Figure 3j), increased oxidative stress (4‐HNE accumulation) and apoptosis (TUNEL‐positive cells) (Figure 3k,l). Conversely, cardiomyocyte‐specific overexpression of human SR‐A3 by AAV9‐*Tnnt2‐hSR‐A3* conferred robust protection, attenuating the rise in LDH and CK (Figure 3n), blunting the inflammatory response (Figure 3o), reducing infarct area (Figure 3p), and suppressing both oxidative damage and apoptosis (Figure 3q,r).

Furthermore, we also evaluated the role of cardiomyocyte‐derived SR‐A3 in the chronic phase of cardiac remodeling. Echocardiography revealed that cardiomyocyte‐specific SR‐A3 knockdown impaired systolic function, reflected by reduced EF, FS, and increased left‐ventricular dimensions (LVIDd, LVIDs), whereas posterior‐wall dimensions (LVPWD and LVPWS) were not significantly altered (Figure S4a). These functional deficits were accompanied by cardiac chamber dilation, hypertrophy, increased ratios of heart weight to body weight and heart weight to tibia length (Figure S4b), enlarged cardiomyocyte size, and more 4‐HNE accumulation and apoptosis (Figure S4c,d).

In contrast, cardiomyocyte‐targeted overexpression of *hSR‐A3* improved chronic post‐I/R cardiac function by enhancing EF and FS while reducing LVIDs, without affecting LVIDd, LVPWD, or LVPWS (Figure S4e). This functional improvement coincided with attenuated chamber enlargement and hypertrophy, lower heart‐weight indices (Figure S4f), and diminished cardiomyocyte size, oxidative stress, and apoptosis (Figure S4g,h).

Together, these gain‐ and loss‐of‐function studies specifically in cardiomyocytes corroborate and extend the observations from global *Sr‐a3* knockout and overexpression models, unequivocally establishing that cardiomyocyte‐autonomous SR‐A3 is a major effector that protects against MI/RI and maladaptive remodeling.

### 2.4 SR‐A3 Confers Cardioprotection Against Ischemia/Reperfusion Injury by Suppressing STING‐Mediated Inflammatory Signaling

**FIGURE 4:**
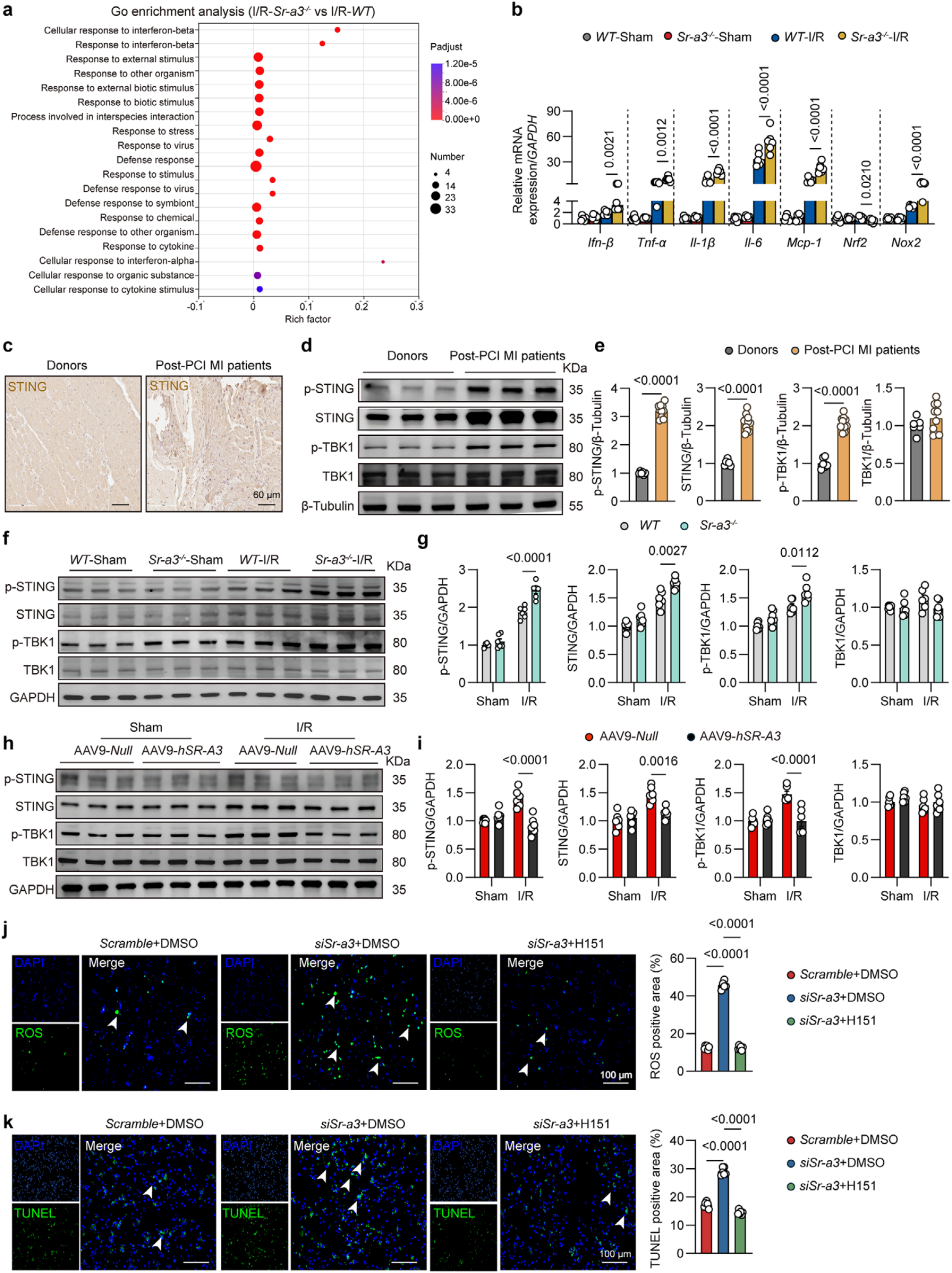
Loss of *Sr‐a3* promotes STING‐mediated inflammatory signaling in myocardial ischemia/reperfusion injury. (a) Gene Ontology (GO) enrichment analysis of cellular responses in *WT* and *Sr‐a3^−/−^* mice after MI/RI. (b) QPCR analysis of *Ifn‐β*, Tnf‐α*, Il‐1β, Il‐6, Mcp‐1*, Nrf2, and Nox2 expression in heart tissues of *WT* and *Sr‐a3^−/−^* mice after MI/RI (*n* = 6 per group). (c) Immunohistochemical staining of STING in paraffin‐embedded human heart tissue sections. (d) Western blot analysis of p‐STING, STING, p‐TBK1, and TBK1 protein expression in human heart tissues (*n* = 6 or 10). (e) Quantification of the data shown in (d). (f) Western blot analysis of p‐STING, STING, p‐TBK1, and TBK1 protein expression in heart tissues of *WT* and *Sr‐a3^−/−^* mice after MI/RI (*n* = 6 per group). (g) Quantification of the data shown in (f). (h) Western blot analysis of p‐STING, STING, p‐TBK1, and TBK1 protein expression in heart tissues of AAV9*‐Null* or AAV9*‐hSR‐A3* infected mice after MI/RI (*n* = 6 per group). (i) Quantification of the data shown in (h). (j) Representative images and quantification of intracellular reactive oxygen species (ROS) staining in NRCMs transfected with scramble+DMSO, *siSr‐a3*+DMSO, or *siSr‐a3*+H151 (a STING inhibitor, 1 µM) followed by H/R treatment (*n* = 6 independent biological replicates; scale bar: 100 µm). ROS‐positive cells are labeled green, and nuclei are counterstained with DAPI (blue). White arrowheads highlight ROS‐positive staining in NRCMs. (k) Representative images and quantification of TUNEL staining in NRCMs under the treatments described in (j) followed by H/R (*n* = 6 independent biological replicates; scale bar: 100 µm). TUNEL‐positive cells are labeled green, and nuclei are counterstained with DAPI (blue). White arrowheads highlight TUNEL‐positive staining in NRCMs. Data are presented as the mean ± SEM and were analyzed by two‐tailed unpaired Student's *t*‐test or one‐ or two‐way ANOVA with Tukey post hoc tests.

To investigate the potential mechanism by which SR‐A3 protects against MI/RI and maladaptive remodeling, we performed transcriptome sequencing on heart tissues collected from *WT* and *Sr‐a3^−/−^* mice. Enrichment analysis of differentially expressed genes demonstrated significant enrichment of pathways related to interferon signaling and responses to external stimuli (Figure 4a). Consistent with the RNA‐sequencing data, qPCR validation confirmed that under MI/RI conditions, *Sr‐a3^−/−^* hearts exhibited markedly elevated mRNA levels of *interferon‐β* (*Ifn‐β*) as well as genes involved in multiple inflammatory responses and oxidative stress compared with *WT* hearts (Figure 4b). Recent evidence implicates dysregulated activation of cyclic GMP‐AMP synthase (cGAS)/stimulator of interferon genes (STING) pathways in various sterile inflammatory conditions, including heart failure, MI, MI/RI and cardiac hypertrophy [12, 13, 14, 15, 16]. Consequently, targeting aberrant STING signaling has emerged as a promising therapeutic strategy for acute or chronic inflammatory diseases. We then conducted immunohistochemical analysis in our clinical specimens, which revealed a significant increase in STING‐positive area in heart tissues from patients who underwent PCI compared with non‐ischemic controls (Figure 4c). Correspondingly, Western blot analysis demonstrated elevated phosphorylation of STING and its downstream effector TANK‐binding kinase 1 (TBK1) in PCI patient samples (Figure 4d,e). In the mouse MI/RI model, genetic deletion of SR‐A3 further upregulated total STING, phosphorylated STING (p‐STING), and phosphorylated TBK1 (p‐TBK1) protein levels (Figure 4f,g). Conversely, cardiomyocyte‐specific overexpression of *hSR‐A3* substantially suppressed the activation of the STING/TBK1 pathway (Figure 4h,i). Additionally, in vitro experiments in NRCMs showed that *Sr‐a3* knockdown promoted intracellular ROS accumulation and increased apoptosis. Notably, these detrimental effects caused by SR‐A3 deficiency were largely reversed by treatment with the STING inhibitor H151 (Figure 4j,k), indicating that the cGAS/STING axis mediates, at least in part, the pathological consequences in the context of SR‐A3 deficiency.

### 2.5 SR‐A3 Blocks STING Signaling by Suppressing STING ISGylation

**FIGURE 5:**
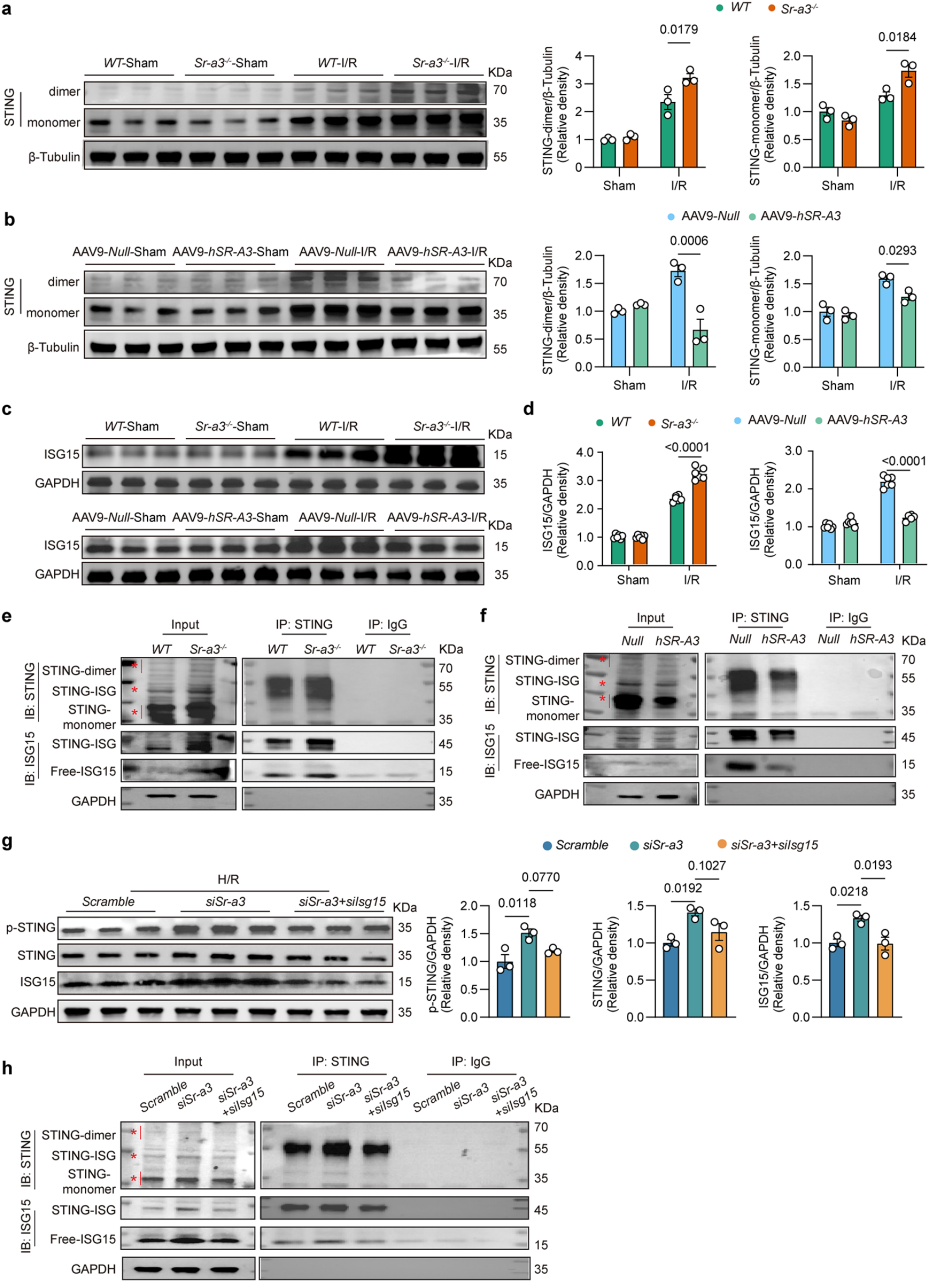
Loss of *Sr‐a3* promotes ISG15‐mediated ISGylation of STING in myocardial ischemia/reperfusion injury. (a) Western blot analysis of STING dimer and monomer levels, with quantification, in heart tissues of *WT* and *Sr‐a3^−/−^* mice after MI/RI (*n* = 3 per group). (b) Western blot analysis of STING dimer and monomer levels, with quantification, in heart tissues of AAV9*‐Null* and AAV9*‐hSR‐A3* infected mice after MI/RI (*n* = 3 per group). (c) Western blot analysis of ISG15 protein expression in heart tissues of *WT*, *Sr‐a3^−/−^*, AAV9*‐Null*, and AAV9*‐hSR‐A3* infected mice after MI/RI (*n* = 6 per group). (d) Quantification of the data shown in (c). (e) Immunoprecipitation (IP) with anti‐STING or control IgG, followed by immunoblot analysis of STING ISGylation and expression of STING, ISG15, and GAPDH in heart tissues of *WT* and *Sr‐a3^−/−^* mice after MI/RI. (f) IP and immunoblot analysis as in (e) using heart tissues from AAV9*‐Null* and AAV9*‐hSR‐A3* infected mice after MI/RI. (g) Western blot analysis of p‐STING, STING, and ISG15 protein expression, with quantification, in NRCMs transfected with *scramble*, *siSr‐a3*, or *siSr‐a3*+*siIsg15*, followed by H/R (*n* = 3 independent biological replicates). (h) IP with anti‐STING or control IgG and immunoblot analysis of STING ISGylation and expression of STING, ISG15, and GAPDH in NRCMs under the treatments described in (g), followed by H/R. Red stars indicate positive protein bands. Data are presented as the mean ± SEM and were analyzed by one‐ or two‐way ANOVA with Tukey post hoc tests.

Increased STING protein expression and its post‐translational modifications, including ISGylation, are highly associated with the activation of the STING signaling pathway [17]. To investigate whether SR‐A3 suppresses STING activation by regulating STING protein abundance, we first assessed STING expression in heart tissues from mice subjected to MI/RI under conditions of either global *Sr‐a3* deficiency or *hSR‐A3* overexpression. Using non‐reducing SDS‐PAGE, we found that both STING monomer and dimer forms were increased in *Sr‐a3*‐deficient hearts after MI/RI, whereas overexpression of *hSR‐A3* reduced both forms, indicating that SR‐A3 negatively regulates STING protein levels (Figure 5a,b).

Previous studies have established that interferon‐stimulated gene 15 (ISG15) promotes polyubiquitination of STING, thereby stabilizing its oligomeric state and amplifying downstream interferon signaling through a positive‐feedback loop [18]. Consistent with this notion, Western blot analysis demonstrated that *Sr‐a3* deficiency upregulated ISG15 expression in MI/RI hearts, while *hSR‐A3* overexpression downregulated ISG15 levels (Figure 5c,d). Co‐immunoprecipitation (Co‐IP) assays further confirmed that loss of *Sr‐a3* intensified ISGylation of STING, whereas *hSR‐A3* overexpression attenuated this modification in MI/RI hearts (Figure 5e).

To delineate the functional interplay between SR‐A3 and ISG15, we performed complementary in vitro experiments in NRCMs. Knockdown of *Sr‐a3* alone led to increased expression of total STING, p‐STING, and p‐TBK1. Notably, concurrent knockdown of *Isg15* effectively counteracted the upregulation of these proteins induced by SR‐A3 deficiency (Figure 5f,g). Moreover, silencing of *Isg15* abolished the enhancement of STING ISGylation that was otherwise observed upon *Sr‐a3* knockdown (Figure 5h).

### 2.6 SR‐A3 Inhibits the ISG15‐STING Axis by Binding and Stabilizing USP18

**FIGURE 6:**
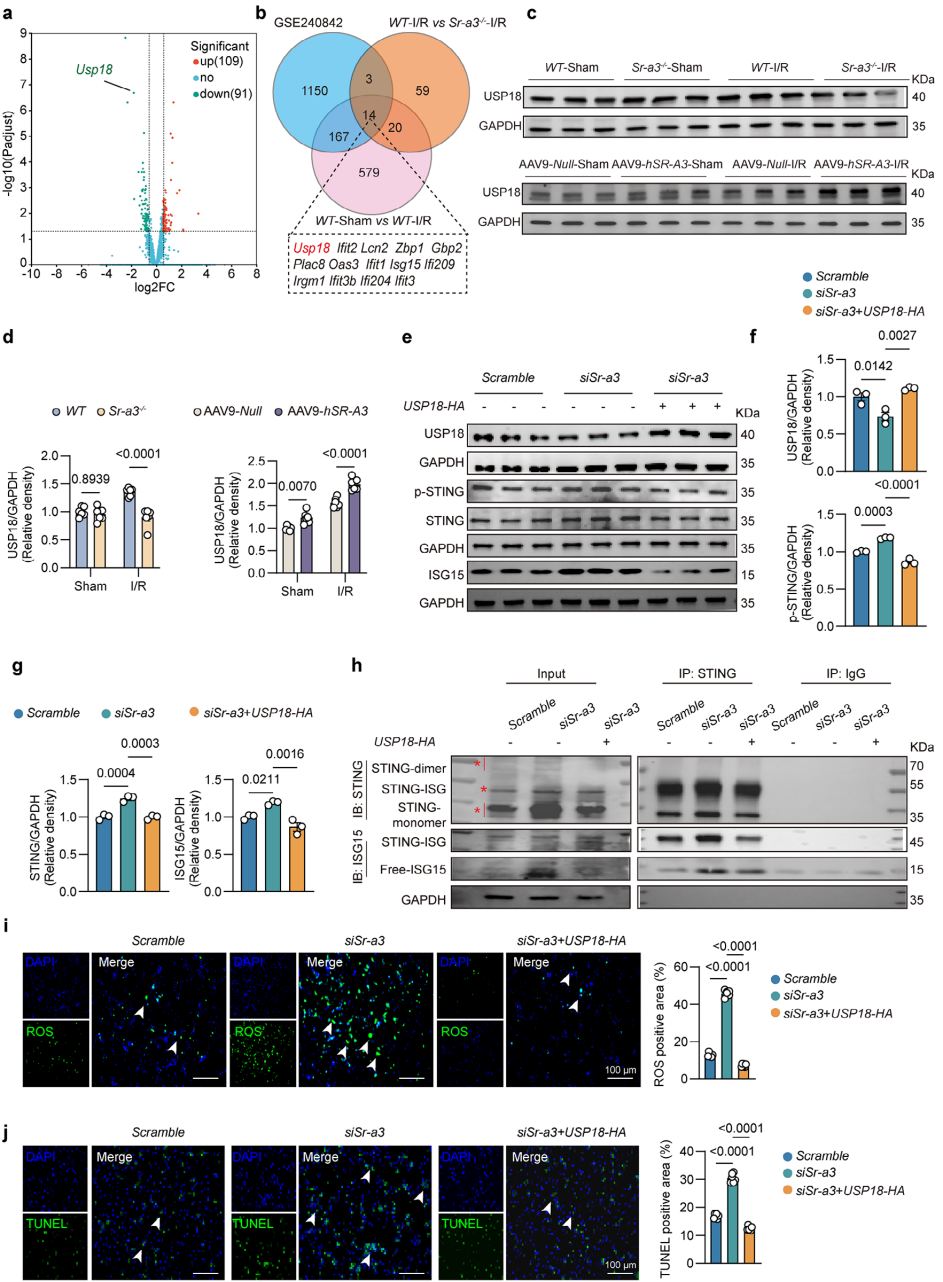
Loss of *Sr‐a3* activates STING‐mediated inflammatory signaling by downregulating USP18. (a) Volcano plots of differentially expressed genes in heart tissues of *WT* and *Sr‐a3^−/−^* mice after MI/RI (*p* value < 0.05 and |log2 fold change| > 1). The top 10 ranked genes by fold change and Usp18 are labeled. (b) Venn diagram showing the overlap of differentially expressed genes from dataset GSE240842, genes altered in *WT* vs. *Sr‐a3^−/−^* mice after MI/RI, and genes altered in sham vs. MI/RI mouse hearts. (c) Western blot analysis of USP18 protein expression in heart tissues of the mouse after MI/RI (*n* = 6 independent biological replicates). (d) Quantification of the data shown in (c). (e) Western blot analysis of USP18, ISG15, p‐STING, and STING protein expression in NRCMs transfected with scramble+empty vector, *siSr‐a3*+empty vector, or *siSr‐a3*+HA‐USP18, followed by H/R (*n* = 3 independent biological replicates). (f, g) Quantification of Western blot in (e). (h) IP with anti‐STING or control IgG and immunoblot analysis of STING ISGylation and expression of STING, ISG15, and GAPDH in NRCMs transfected with scramble+empty vector, *siSr‐a3*+empty vector, or *siSr‐a3*+HA‐USP18, followed by H/R. Red stars indicate positive protein bands. (i) Representative images and quantification of intracellular ROS staining in NRCMs under the treatments described in (e) (*n* = 6 independent biological replicates; scale bar: 100 µm). ROS‐positive cells are labeled green, and nuclei are counterstained with DAPI (blue). White arrowheads highlight ROS‐positive staining in NRCMs. (j) Representative images and quantification of TUNEL staining in NRCMs under the treatments described in (e) (*n* = 6 independent biological replicates; scale bar: 100 µm). TUNEL‐positive cells are labeled green, and nuclei are counterstained with DAPI (blue). White arrowheads highlight TUNEL‐positive staining in NRCMs. Data are presented as the mean ± SEM and were analyzed by one‐ or two‐way ANOVA with Tukey post hoc tests.

Since our preceding findings indicated that SR‐A3 suppresses ISG15 expression in cardiac tissue, to uncover how SR‐A3 regulates ISG15, we performed transcriptome sequencing of heart tissue collected from *WT* and *Sr‐a3^−/−^* mice. Compared with *WT* hearts, *Sr‐a3^−/−^* mouse hearts showed 109 significantly upregulated and 91 downregulated genes (Figure 6a). Subsequent intersection analysis of these differentially expressed genes with two independent MI/RI RNA‐sequencing datasets, including GSE240842 with 30 min ischemia/4 h reperfusion and the one generated in our laboratory under the condition of 30 min ischemia/24 h reperfusion, identified 14 overlapping genes, among which the deubiquitinating enzyme USP18 ranked highest (Figure 6b). We next examined USP18 expression in hearts from mice with either global *Sr‐a3* deletion or overexpression of *hSR‐A3*. Western blot analyses demonstrated that deletion of *Sr‐a3* reduced USP18 protein levels in MI/RI hearts, while *hSR‐A3* overexpression substantially increased USP18 expression when compared to their corresponding control animals (Figure 6c,d). In NRCMs, overexpression of *USP18* reversed the downregulation of USP18 and attenuated the upregulation of total STING and p‐STING caused by *Sr‐a3* knockdown (Figure 6e–g). Co‐IP assays further confirmed that *USP18* overexpression suppressed the enhanced ISGylation of STING observed upon *Sr‐a3* knockdown (Figure 6h). Immunofluorescence staining indicated that knockdown of *Sr‐a3* elevated intracellular ROS accumulation and increased apoptosis, and these effects were largely rescued by USP18 overexpression (Figure 6i,j).

**FIGURE 7:**
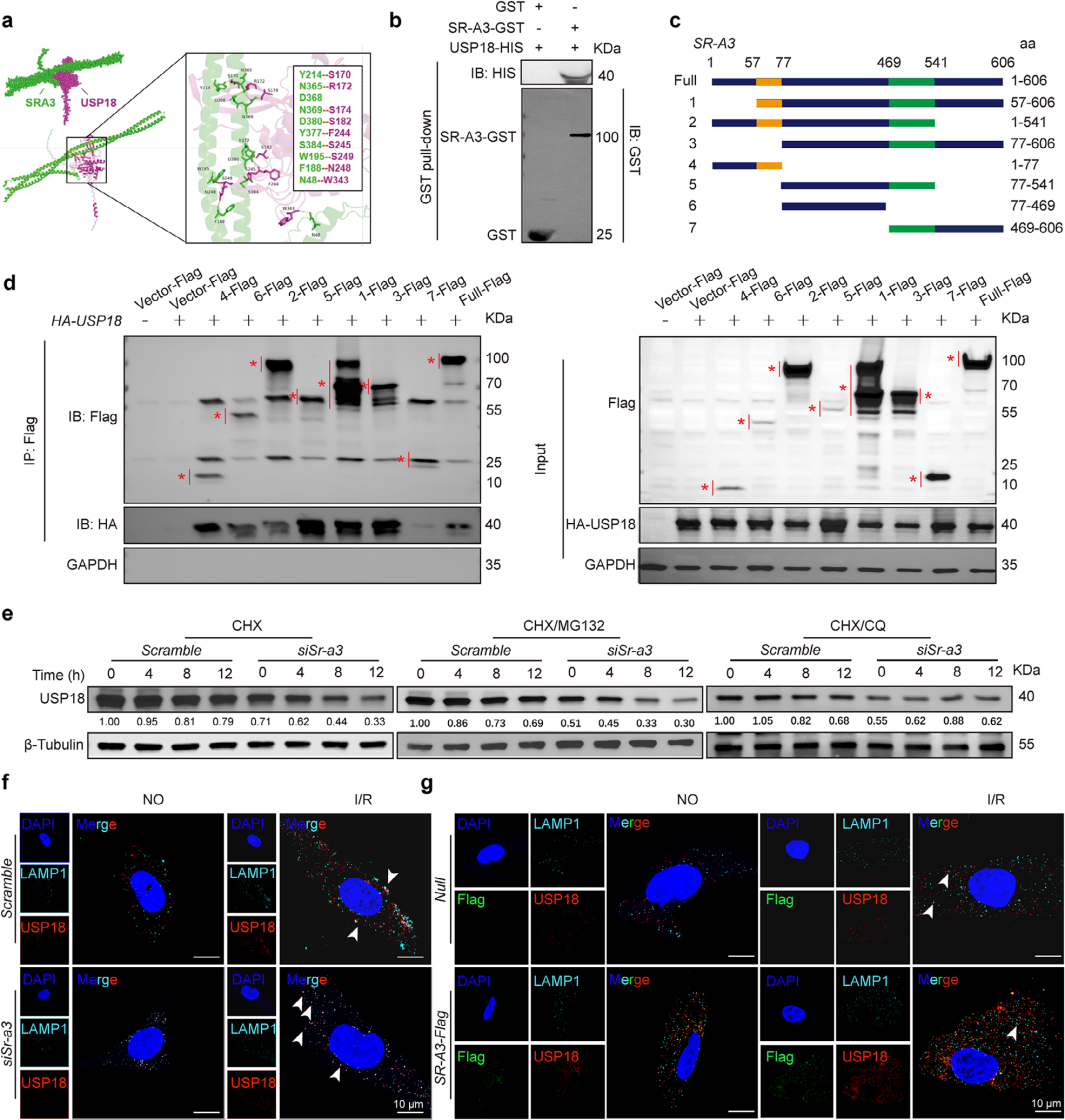
SR‐A3 stabilizes USP18 to inhibit the ISG15/STING axis. (a) Predicted molecular docking model of the SR‐A3 and USP18 interaction. (b) GST‐pull down analysis of the interaction between USP18‐HIS and SR‐A3‐GST. (c) Schematic diagram of wild‐type FLAG‐SR‐A3 and its truncated mutants. (d) Co‐IP analysis of the interaction between HA‐USP18 and FLAG‐SR‐A3 or its truncated forms in HEK293T cells. (e) Western blot analysis of USP18 protein levels in NRCMs transfected with scramble or *siSr‐a3* and treated with PBS, MG132 (proteasome inhibitor), or chloroquine (CQ, autophagy inhibitor) in the presence of cycloheximide (CHX) for the indicated durations. (f) Representative confocal images of LAMP1 (lysosomal marker) and USP18 staining in NRCMs transfected with scramble or *siSr‐a3* followed by H/R (scale bar: 100 µm). LAMP1 signal is cyan; USP18 is red; nuclei are DAPI‐stained (blue). (g) Representative confocal images of LAMP1, USP18, and FLAG‐hSR‐A3 staining in NRCMs transfected with empty vector or FLAG‐hSR‐A3 followed by H/R (scale bar: 100 µm). LAMP1 is cyan; USP18 is red; FLAG‐hSR‐A3 is green; nuclei are DAPI‐stained (blue). Red stars indicate positive protein bands. White arrowheads indicate the positive staining of SR‐A3 and LAMP1.

Our previous study demonstrated that SR‐A3 can directly bind to E3 ubiquitin ligases, thereby stabilizing their expression and modulating downstream signaling [11]. Extending this work, we used the HDOCK database to predict a direct interaction between SR‐A3 and USP18 (Figure 7a). This prediction was experimentally validated by GST‐pull down assay, confirming a physical association between SR‐A3 and USP18 (Figure 7b). As SR‐A3 is a multi‐domain transmembrane glycoprotein, to map the regions responsible for its interaction with USP18, we constructed a series of truncated SR‐A3 mutants. Our results from co‐transfection of each mutant with USP18‐HA into HEK293T cells, followed by immunoprecipitation, revealed that both the 1–77 and 77–469 domains of SR‐A3 could bind USP18 (Figure 7c,d).

To investigate how SR‐A3 influences USP18 stability, we inhibited protein synthesis with cycloheximide (CHX) and concurrently blocked either lysosomal degradation with chloroquine (CQ) or proteasomal degradation with MG132. USP18 degradation was attenuated only when CHX was combined with CQ (Figure 7e), indicating that SR‐A3 stabilizes USP18 primarily via the lysosomal pathway. Co‐staining of USP18 with the lysosomal marker lysosomal‐associated membrane protein 1 (LAMP1) showed that *Sr‐a3* knockdown reduced USP18 abundance and enhanced its colocalization with LAMP1 (Figure 7f). Conversely, overexpression of *hSR‐A3* increased USP18 levels and diminished its colocalization with LAMP1 (Figure 7g). Together, these data establish that SR‐A3 binds directly to USP18, protects it from lysosomal degradation, and consequently enhances USP18 protein stability.

### 2.7 Pharmacological Activation of USP18 With Selonsertib Mitigates Myocardial Ischemia/Reperfusion Injury

**FIGURE 8:**
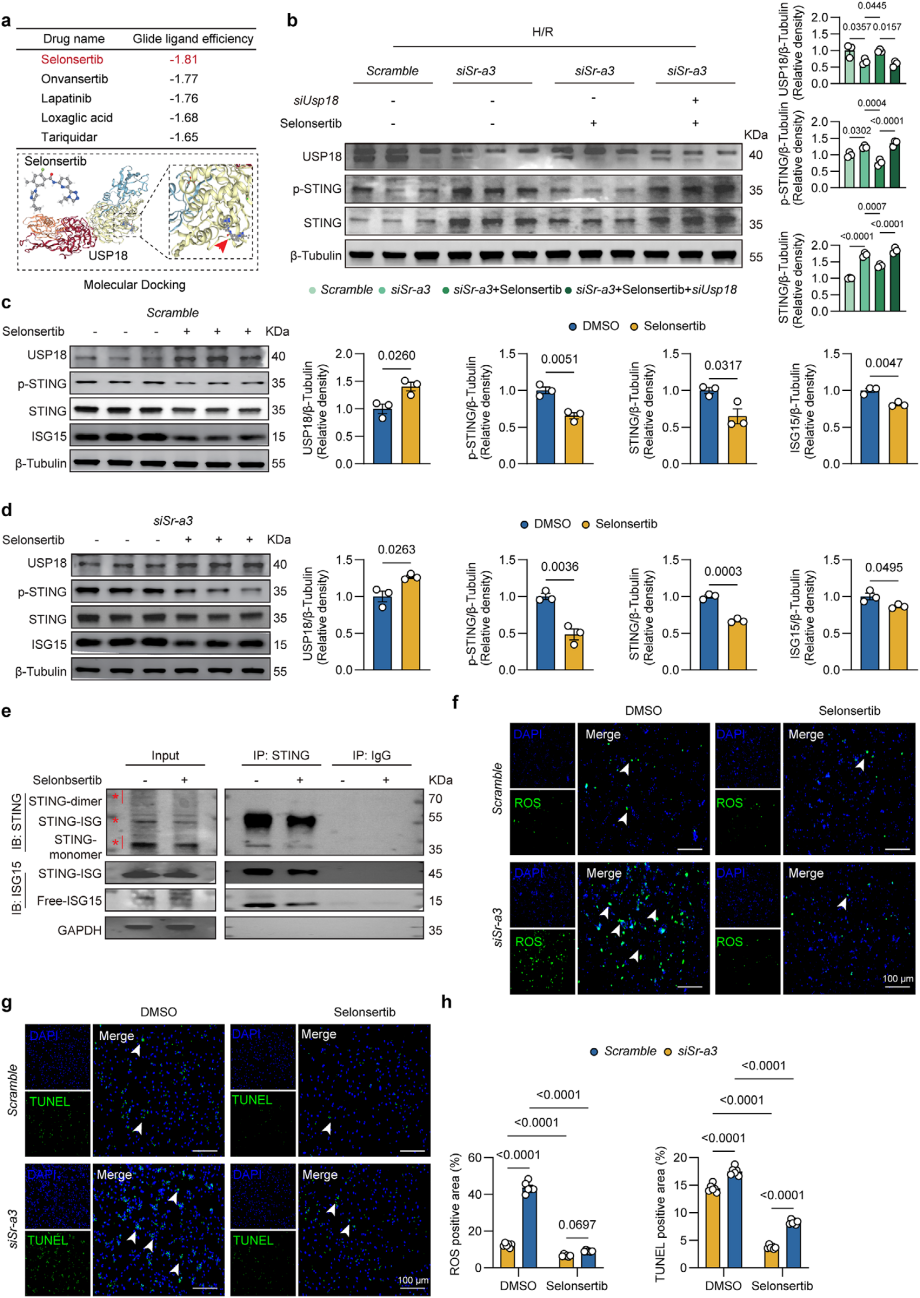
Pharmacological activation of USP18 by Selonsertib ameliorates H/R injury in vitro by suppressing STING signaling. (a) Chemical structure of Selonsertib and a schematic of its proposed mechanism of action. The red arrowhead indicates the binding site of Selonsertib on USP18. (b) Western blot analysis of USP18, p‐STING, and STING protein expression, with quantification, in NRCMs treated as indicated (scramble+DMSO, *siSr‐a3*+DMSO, *siSr‐a3*+Selonsertib, or *siSr‐a3*+Selonsertib+*siUsp18*) followed by H/R (*n* = 3 per group, independent biological replicates). (c) Western blot analysis of USP18, p‐STING, STING, and ISG15 protein expression, with quantification, in NRCMs treated with DMSO or Selonsertib followed by H/R (*n* = 3 per group, independent biological replicates). (d) Western blot analysis as in (c) for NRCMs transfected with *siSr‐a3* and treated with DMSO or Selonsertib followed by H/R (*n* = 3 per group, independent biological replicates). (e) Co‐IP with anti‐STING or control IgG and immunoblot analysis of STING ISGylation and expression of STING, ISG15, and GAPDH in NRCMs transfected with *siSr‐a3* or *siSr‐a3*+Selonsertib followed by H/R. Red stars indicate positive protein bands. (f) Representative images and quantification of intracellular ROS staining in NRCMs under the indicated treatments followed by H/R (*n* = 6 per group; scale bar: 100 µm). ROS‐positive cells are labeled green, and nuclei are counterstained with DAPI (blue). White arrowheads highlight ROS‐positive staining in NRCMs. (g) Representative images and quantification of TUNEL staining in NRCMs under the indicated treatments followed by H/R (*n* = 6 per group; scale bar: 100 µm). TUNEL‐positive cells are labeled green, and nuclei are counterstained with DAPI (blue). White arrowheads highlight TUNEL‐positive staining in NRCMs. (h) Statistical summary of the data shown in (f, g). Data are presented as the mean ± SEM and were analyzed by two‐tailed unpaired Student's *t*‐test or one‐ or two‐way ANOVA with Tukey post hoc tests.

To identify potential therapeutic interventions for MI/RI associated with *Sr‐a3* deficiency, we conducted large‐scale drug screening using molecular docking with FDA‐approved or investigational small molecules, evaluating their binding affinity and free energy toward USP18 (Figure 8a). Among the five compounds tested, Selonsertib, an ASK1 inhibitor, significantly upregulated USP18 expression under *Sr‐a3* knockdown conditions (Figure 8b). After assessing the concentration‐dependent effects on cytotoxicity and activation of the USP18downstream signaling pathway, we selected 10 µm Selonsertib to treat NRCMs for 12 h (Figure S5). In NRCMs transfected with either scramble control or *siSr‐a3*, Selonsertib markedly reduced the protein levels of ISG15, total STING, and p‐STING by upregulating USP18 expression (Figure 8c,d). Furthermore, Co‐IP assays confirmed that Selonsertib treatment inhibited STING ISGylation (Figure 8e). Consistent with these molecular alterations, immunofluorescence analysis showed that Selonsertib decreased intracellular ROS accumulation and attenuated NRCM apoptosis upon hypoxia/reoxygenation (H/R) stimulation, both under control and *Sr‐a3* knockdown conditions (Figure 8f–h).

**FIGURE 9:**
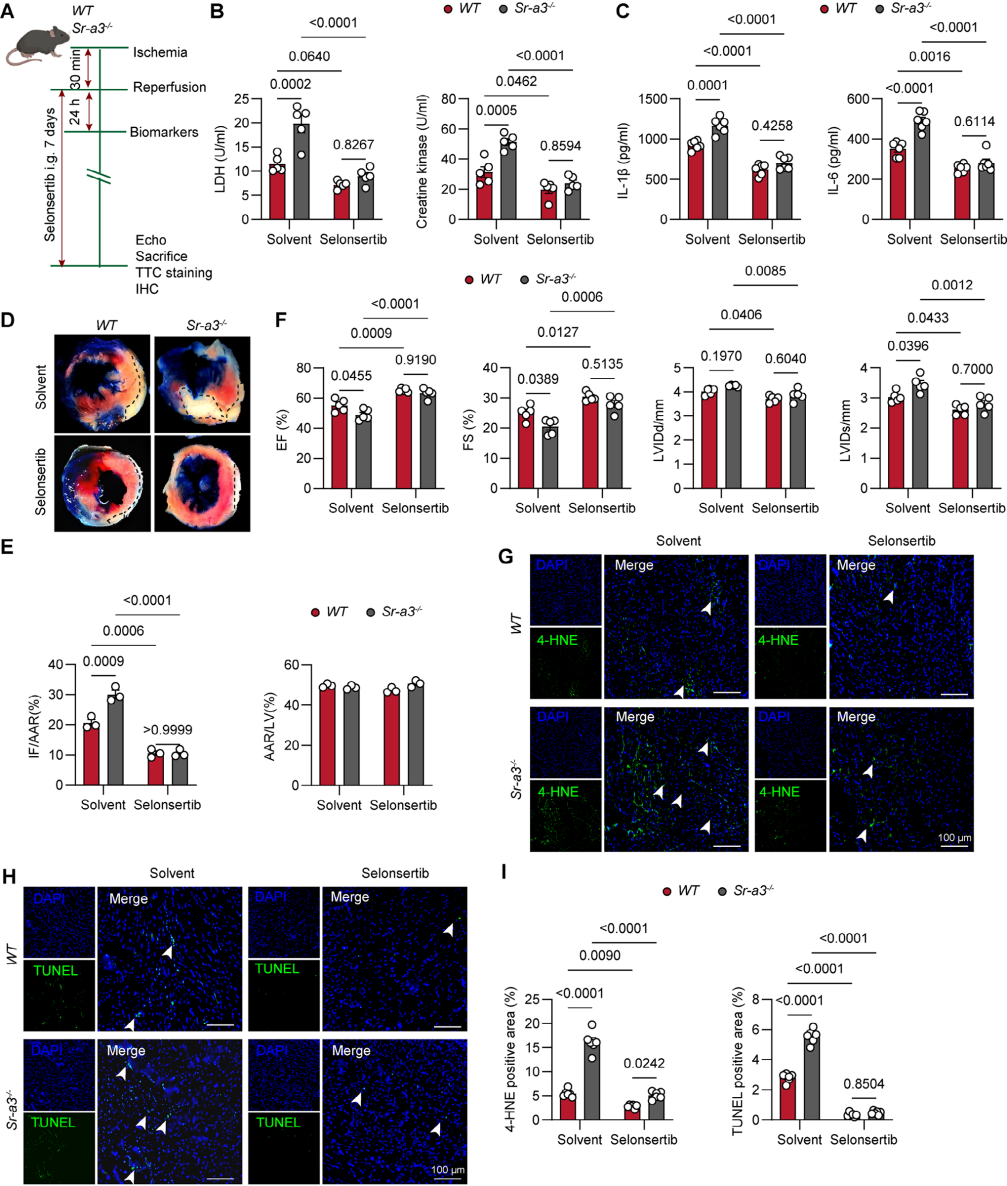
Selonsertib effectively ameliorates MI/RI in vivo. (a) Schematic diagram of the experimental protocol. Selonsertib (10 mg/kg) or vehicle was administered by oral gavage daily from the day of MI/RI surgery until day 7. Blood samples were collected at 24 h after reperfusion for LDH and CK measurement. Echocardiography and tissue collection were performed at day 7 post‐surgery. (b) Plasma LDH and CK levels in *WT* and *Sr‐a3^−/−^* mice treated with or without Selonsertib after MI/RI (30 min ischemia/7 d reperfusion; *n* = 5 per group). (c) Plasma levels of IL‐1β and IL‐6 in the mouse groups described in (b). (d) Representative Evans blue/TTC‐stained cardiac sections from the mouse groups described in (b). Black dashed lines outline the myocardial infarction area. (e) Quantification of infarct size from the data shown in (d). (f) Echocardiographic assessment of cardiac function. EF, ejection fraction; FS, fractional shortening; LVIDd, left ventricular internal diameter at diastole; LVIDs, left ventricular internal diameter at systole. (*n* = 6 independent biological replicates). (g) Representative 4‐HNE‐stained cardiac sections from the mouse groups described in (b) (scale bar: 100 µm). 4‐HNE‐positive cells are labeled green, and nuclei are counterstained with DAPI (blue). White arrowheads highlight 4‐HNE‐positive staining in mouse heart tissues. (h) Representative TUNEL‐stained cardiac sections from the mouse groups described in (b) (scale bar: 100 µm). TUNEL‐positive cells are labeled green, and nuclei are counterstained with DAPI (blue). White arrowheads highlight TUNEL‐positive staining in mouse heart tissues. (i) Quantification of the 4‐HNE‐positive and TUNEL‐positive areas from the data shown in (g, h). (*n* = 6 independent biological replicates) Data are presented as the mean ± SEM and were analyzed by two‐way ANOVA with Tukey post hoc tests.

We next evaluated the therapeutic potential of Selonsertib in vivo using a mouse model of MI/RI by oral administration (10 mg/kg/day) (Figure 9a). Notably, Selonsertib administration effectively mitigated cardiac damage with decreased serum levels of LDH and CK (Figure 9b), reduced inflammatory markers (Figure 9c), and smaller infarct sizes (Figure 9d,e). Furthermore, Selonsertib improved cardiac function after MI/RI by increasing EF and FS while reducing LVIDd and LVIDs (Figure 9f). Pathological assessment confirmed that Selonsertib also suppressed oxidative stress and apoptosis in heart tissues from both *WT* and *Sr‐a3^−/−^* mice (Figure 9g,h). Collectively, these data indicate that Selonsertib confers substantial cardioprotection against MI/RI irrespective of SR‐A3 expression status.

### 2.8 Cardiomyocyte‐Specific USP18 Knockdown Attenuates Selonsertib‐Mediated Cardioprotection in Myocardial Ischemia/Reperfusion Injury

**FIGURE 10:**
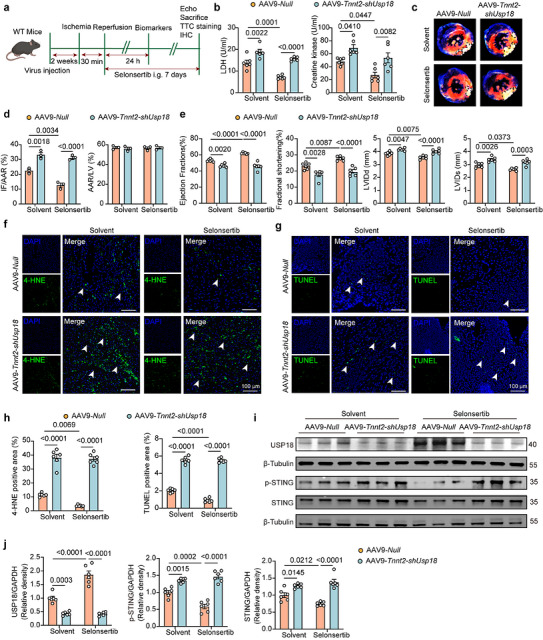
Cardiomyocyte‐specific USP18 knockdown abolishes the cardioprotective effects of Selonsertib during myocardial ischemia/reperfusion injury. (a) Schematic illustration of the experimental design. WT mice were injected with AAV9‐Null or AAV9‐Tnnt2‐shUsp18. Two weeks after viral injection, mice were subjected to 30 min myocardial ischemia followed by reperfusion. Selonsertib (10 mg/kg) or vehicle was administered by oral gavage once daily from the day of MI/RI surgery until day 7. Blood samples were collected at 24 h after reperfusion for serum LDH and CK measurement. Echocardiography was performed at day 7 post‐surgery, followed by tissue harvest for TTC staining, Western blot, and histological analyses. (b) Serum LDH and creatine kinase levels in AAV9‐*Null* or AAV9‐*Tnnt2‐shUsp18* mice treated with solvent or Selonsertib after MI/RI. (*n* = 6 independent biological replicates) (c) Representative TTC staining images of hearts from the indicated groups after MI/RI. (d) Quantification of infarct size normalized to the area at risk (IF/AAR) and area at risk normalized to the left ventricle (AAR/LV). (*n* = 3 independent biological replicates) (e) Echocardiographic assessment of cardiac function, including ejection fraction, fractional shortening, LVIDd, and LVIDs, in the indicated groups after MI/RI. (*n* = 6 independent biological replicates) (f) Representative immunofluorescence images of 4‐HNE staining in heart sections from the indicated groups. 4‐HNE‐positive signals are shown in green, and nuclei are counterstained with DAPI. Scale bar, 100 µm. (g) Representative TUNEL staining images in heart sections from the indicated groups. TUNEL‐positive signals are shown in green, and nuclei are counterstained with DAPI. Scale bar, 100 µm. (h) Quantification of 4‐HNE‐positive area and TUNEL‐positive area in the indicated groups. (*n* = 6 independent biological replicates) (i) Western blot analysis of USP18, p‐STING, and STING protein levels in heart tissues from the indicated groups after MI/RI. (j) Densitometric quantification of USP18, p‐STING, and STING protein levels shown in (i). (*n* = 6 independent biological replicates) Data are presented as the mean ± SEM and were analyzed by two‐way ANOVA with Tukey post hoc tests.

To further determine whether USP18 is required for the cardioprotective action of Selonsertib in vivo, we performed cardiomyocyte‐targeted USP18 knockdown using AAV9‐*Tnnt2‐shUsp18* before MI/RI and then treated mice with Selonsertib or solvent (Figure 10a). Compared with AAV9‐*Null* controls, AAV9‐*Tnnt2‐shUsp18* mice displayed more severe myocardial injury after MI/RI, as evidenced by elevated serum LDH and CK levels, larger infarct size normalized to the area at risk, impaired systolic function, increased ventricular dilation, and enhanced 4‐HNE and TUNEL‐positive areas (Figure 10b–h). In AAV9‐*Null* mice, Selonsertib markedly reduced serum LDH and CK release, decreased infarct size, improved echocardiographic parameters, and suppressed myocardial oxidative stress and apoptosis (Figure 10b–h). Notably, these protective effects were largely abolished by cardiomyocyte‐specific USP18 knockdown. Although the area at risk relative to the left ventricle was comparable among groups, AAV9‐*Tnnt2‐shUsp18* mice remained highly susceptible to MI/RI despite Selonsertib treatment, showing persistently increased infarct size, reduced EF and FS, enlarged LVIDd and LVIDs, and sustained 4‐HNE and TUNEL signals (Figure 10c–h).

Consistent with these phenotypic findings, Western blot analysis showed that Selonsertib increased myocardial USP18 expression and suppressed STING activation in AAV9‐*Null* mice, whereas AAV9‐*Tnnt2‐shUsp18* markedly reduced USP18 abundance and prevented Selonsertib‐mediated inhibition of p‐STING and total STING (Figure 10i,j). These in vivo loss‐of‐function data demonstrate that USP18 is indispensable for the major cardioprotective effects of Selonsertib during MI/RI, supporting the conclusion that Selonsertib acts predominantly through activation of the USP18/STING regulatory axis rather than solely through ASK1 inhibition.

## 3 Discussion

MI/RI remains a major unresolved clinical challenge despite timely revascularization strategies. In the present study, we identify SR‐A3 as a previously unrecognized cardioprotective factor in the heart. Using both loss‐ and gain‐of‐function approaches, we demonstrate that *Sr‐a3* deficiency exacerbates MI/RI, whereas cardiomyocyte‐specific overexpression conferred robust protection against acute injury and chronic remodeling. Mechanistically, SR‐A3 restrains ischemia/reperfusion‐induced innate immune activation by interacting with and stabilizing USP18, thereby suppressing ISG15‐dependent STING ISGylation, STING activation, and then inflammatory response. These findings uncover a previously unappreciated intracellular regulatory axis governing innate immune activation in cardiomyocytes.

A key conceptual advance of this study lies in expanding the functional paradigm of scavenger receptors in cardiovascular disease. Classical scavenger receptors, such as SR‐A1, are generally viewed as plasma membrane pattern‐recognition receptors that recognize modified lipoproteins and danger‐associated ligands, thereby linking extracellular danger sensing to inflammatory responses [19, 20]. In contrast, SR‐A3 is predominantly localized to intracellular compartments, including the endoplasmic reticulum and Golgi apparatus, suggesting that it may regulate intracellular stress responses and protein homeostasis rather than simply functioning as a cell‐surface ligand‐recognition receptor [6, 10]. This intracellular feature is highly consistent with our mechanistic finding that SR‐A3 stabilizes USP18 and limits ISG15‐dependent STING activation. Moreover, comparative transcriptomic analysis of scavenger receptor family members in human and mouse MI/RI‐related datasets showed that SCARA3/SR‐A3 was consistently downregulated after ischemia/reperfusion injury. Together with the known role of SR‐A3 in protecting against oxidative stress‐induced cellular damage and our previous finding that SR‐A3 regulates metabolic and inflammatory homeostasis, these observations provide a strong rationale for focusing on SR‐A3 in the context of MI/RI.

Although cardiomyocyte‐targeted gain‐ and loss‐of‐function experiments support a major cardiomyocyte‐autonomous role for SR‐A3, we do not interpret this as evidence that cardiomyocytes are the only relevant cell type. By comparing SR‐A3 expression across four major cardiac cell populations after MI/RI, we found that macrophages retained relatively higher SR‐A3 expression, whereas cardiomyocytes exhibited the most profound reduction (over 80%), followed by fibroblasts (∼ 40%) and macrophages (∼ 25%), with endothelial cells showing no significant change. This differential vulnerability, combined with the observation that cardiomyocyte‐specific AAV9‐Tnnt2‐mediated manipulation of SR‐A3 was sufficient to recapitulate the major phenotypes observed in global knockout mice, supports cardiomyocyte‐derived SR‐A3 as a critical contributor to protection against MI/RI. Nevertheless, we acknowledge that SR‐A3 in fibroblasts, macrophages, endothelial cells, or other cardiac stromal and immune cells may also participate in shaping the myocardial injury microenvironment, and their relative contributions warrant further investigation using cell‐type‐specific genetic models.

Innate immune activation has emerged as a central driver of cardiomyocyte death and adverse remodeling following ischemic injury [21, 22, 23]. Among these pathways, cGAS‐STING signaling is particularly relevant because damaged mitochondria can release cytosolic DNA, triggering STING activation and type I interferon responses [13, 24]. Our data show that SR‐A3 deficiency markedly enhances STING phosphorylation, ISGylation, and downstream TBK1 activation, whereas SR‐A3 overexpression suppresses these events. Importantly, these protein‐level changes are central to our interpretation because STING activation is primarily controlled by post‐translational and trafficking events, including ER‐to‐Golgi translocation, oligomerization, phosphorylation, and modification by ubiquitin‐like proteins. Therefore, transcriptional enrichment of interferon‐related pathways alone should not be interpreted as definitive evidence of STING activation. In this study, direct biochemical evidence supports activation of the STING pathway under SR‐A3‐deficient conditions.

Our study further clarifies the relationship between ISG15 and STING in the SR‐A3‐deficient setting. ISG15 was selected through an omics‐based intersection analysis and was subsequently validated in human post‐PCI cardiac samples, SR‐A3‐deficient mouse hearts, and SR‐A3‐overexpressing hearts. Functionally, Isg15 knockdown reduced STING oligomerization, STING ISGylation, and STING/TBK1 activation induced by SR‐A3 deficiency, supporting a model in which ISG15 acts upstream of STING activation in this context. Conversely, pharmacological inhibition of STING with H‐151 suppressed STING/TBK1 activation but did not fully abolish the increase in ISG15 induced by SR‐A3 deficiency. These findings suggest that ISG15 upregulation is not merely a secondary consequence of STING activation, although a positive‐feedback contribution from STING‐dependent interferon signaling cannot be completely excluded.

Recent studies also suggest that STING may contribute to MI/RI progression by promoting ferroptosis. For example, STING activation has been reported to aggravate ferroptosis‐dependent myocardial ischemia/reperfusion injury, at least in part by promoting GPX4 degradation, lipid peroxidation, and iron‐dependent cardiomyocyte injury [13]. These findings suggest that STING may act as a key stress‐signaling hub linking mitochondrial DNA sensing, inflammatory cytokine production, oxidative lipid damage, and regulated cardiomyocyte death during MI/RI. In the present study, we did not directly assess ferroptosis‐related indicators, such as GPX4 activity or iron accumulation. Nevertheless, given that SR‐A3 suppresses ISG15‐dependent STING activation, we speculate that the SR‐A3/USP18/ISG15 axis may indirectly influence cardiomyocyte susceptibility to ferroptosis by limiting excessive STING signaling. Future studies are warranted to determine whether SR‐A3‐mediated STING inhibition attenuates ferroptosis and whether ferroptosis contributes to the protective effects observed in SR‐A3‐overexpressing hearts.

USP18 is a key interferon‐stimulated gene product with dual functions as a deISGylating enzyme and a negative regulator of type I interferon signaling, which has been implicated in several disease conditions, including viral infection, cancer, and neurodegeneration [25, 26, 27]. Prior studies have increasingly recognized USP18‐mediated deISGylation as a mechanism for restraining innate immune signaling [28, 29, 30]. Thus, the novelty of the present study does not simply lie in identifying USP18 as a negative regulator of STING. Instead, our data define SR‐A3 as an upstream regulator that maintains USP18 abundance in cardiomyocytes during MI/RI. Co‐immunoprecipitation and domain‐mapping experiments support an association between SR‐A3 and USP18, and GST pull‐down assays further support a direct physical interaction. Functionally, SR‐A3 protects USP18 from lysosomal degradation, thereby enhancing USP18 stability and preserving its ability to suppress ISG15‐dependent STING activation. This mechanism distinguishes SR‐A3 from classical plasma membrane scavenger receptors and reveals a previously unrecognized intracellular role for SR‐A3 in controlling post‐translational immune signaling. From a translational perspective, our study identifies pharmacological modulation of the USP18/ISG15/STING axis as a potential strategy for limiting MI/RI. Through compound screening, we found that the ASK1 inhibitor Selonsertib increased USP18 abundance in our MI/RI model and suppressed ISG15‐STING signaling. Importantly, since Selonsertib is a well‐established ASK1 inhibitor [31, 32], it should not be described simply as a pharmacological activator of USP18. We therefore interpret the Selonsertib data cautiously. Under our experimental conditions, MI/RI and SR‐A3 deficiency did not induce a consistent pattern of ASK1 activation, and Selonsertib‐mediated cardioprotection was not accompanied by a corresponding suppression of ASK1 phosphorylation. Moreover, cardiomyocyte‐targeted Usp18 knockdown markedly weakened the ability of Selonsertib to reduce serum LDH and CK release, inflammatory cytokine induction, infarct size, oxidative stress, apoptosis, and post‐MI/RI cardiac dysfunction. These findings support the conclusion that in this MI/RI setting, Selonsertib confers cardioprotection at least in large part through USP18‐dependent suppression of ISG15‐mediated STING activation. Nevertheless, potential contributions from canonical ASK1‐related signaling or other off‐target pathways cannot be completely excluded.

Several limitations and open questions warrant attention. First, the present study focused primarily on cardiomyocyte‐derived SR‐A3. The roles of SR‐A3 in cardiac fibroblasts, macrophages, endothelial cells, and other stromal or immune cell populations remain to be investigated using cell type‐specific genetic models. Second, although our data support the SR‐A3/USP18/ISG15/STING axis as a major mechanism, SR‐A3 and USP18 may also influence other stress‐related pathways, including ER stress, mitochondrial homeostasis, metabolic regulation, cell death programs, and broader inflammatory signaling. Finally, further studies in larger animal models and human cohorts are needed before this pathway can be advanced toward clinical translation.

In conclusion, we identify SR‐A3 as a novel endogenous protector against MI/RI. By stabilizing USP18 and suppressing ISG15‐dependent STING activation, SR‐A3 functions as a critical brake on inflammation in cardiomyocytes, with its stress‐induced depletion in these cells representing a key determinant of I/R injury severity. The identification of this signaling axis, together with the therapeutic potential of Selonsertib, provides new mechanistic insights and opens avenues for the development of targeted interventions to limit reperfusion injury and improve clinical outcomes in ischemic heart disease.

## 4 Methods

### 4.1 Sex as a Biological Variable

For animal models, only male mice were used. For clinical samples, both sexes were involved. Sex was not considered a biological variable.

### 4.2 Human Studies

Tissue specimens from the ischemic border zone were obtained from a subset of post‐percutaneous coronary intervention (PCI) myocardial infarction (MI) patients who progressed to heart failure or ventricular aneurysm and subsequently required surgical management (left ventricular assist device implantation or aneurysm resection) at Beijing Anzhen Hospital. After written informed consent was secured, samples were collected intraoperatively and either immersed in fixative or snap‐frozen within 60 min of excision. Non‐ischemic control myocardial specimens, free from any cardiovascular pathology, were obtained during autopsies from donors at the Affiliated Hospital of Chifeng University, with a postmortem interval restricted to ≤ 6 h and a warm ischemic time limited to ≤ 30 min. All donors had previously provided written informed consent for the use of their tissues in research. All tissues were harvested from the left ventricular anterior wall. The experimental protocols involving human biological materials received ethical approval from the ethics committee of both the Affiliated Hospital of Chifeng University (approval No. 2025060) and Beijing Anzhen Hospital (approval No. KS2024104). Detailed clinical characteristics of all enrolled participants are summarized in Table S1.

### 4.3 Animal Model of Myocardial Ischemia/Reperfusion Injury (MI/RI)

All animal experiments were approved by the Animal Ethics Committee of Peking University Third Hospital (SA2024425) and conducted in accordance with the National Institutes of Health Guide for the Care and Use of Laboratory Animals (NIH Publications, eighth edition, 2011).

An MI/RI model was established using 8‑ to 12‑week‑old male C57BL/6J mice. Mice were anesthetized via intraperitoneal injection of 1% sodium pentobarbital (70 mg/kg) and placed on a temperature‐controlled platform maintained at 37°C. The precordial area was disinfected with iodophor. An approximately 2‑cm incision was made between the third and fourth ribs on the left sternal border using sterile scissors, and a reversible snare occluder was placed around the left anterior descending coronary artery. Myocardial ischemia was induced by tightening the snare for 30 min, followed by reperfusion for 24 h (acute injury model) or 4 weeks (chronic model). After reperfusion, mice were euthanized for tissue collection. Prior to euthanasia, animals received sodium pentobarbital (100 mg/kg) and heparin (400 USP U/kg) intraperitoneally. Blood samples were collected and centrifuged at 4000 rpm for 10 min to obtain serum. Serum lactate dehydrogenase (LDH) activity was measured spectrophotometrically using a commercial kit (Shanghai Gensource, Cat# LDH0360). Creatine kinase (CK) levels were determined with a corresponding kit (Shanghai Gensource, Cat# CPK0300). The ischemic left ventricular region was harvested for biochemical analysis.

For the in vivo Selonsertib treatment experiments (Figures 9 and 10), Selonsertib (10 mg/kg) or vehicle control was administered by oral gavage once daily starting immediately after MI/RI surgery and continued for 7 consecutive days. At 24 h after reperfusion, blood samples were collected to measure serum LDH and CK levels. At the end of the 7‐day treatment period, cardiac function was assessed by echocardiography, and heart tissues were harvested for Evans blue/TTC staining, Western blot analysis, and histological examinations. The experimental timeline is illustrated in the schematic diagrams in Figures 9a and 10a.

### 4.4 Statistical Analysis

Data were analyzed using GraphPad Prism 9.0. Continuous variables are presented as mean ± SEM. Two‐group comparisons were made with 2‐sided unpaired Student's *t*‐test (normally distributed, equal variance). Multiple comparisons were performed with one‐way ANOVA (single variable) or two‐way ANOVA (multiple variables), followed by Tukey's test. Nonparametric data were analyzed with the Mann–Whitney U‐test or Kruskal–Wallis test. *p* < 0.05 was considered statistically significant.

## Author Contributions

Conceptualization: X.X., Y.H.; Methodology: Y.H., G.M., Y.Z., K.L., A.X., J.D., Z.Z, J.C. (**Jinxuan Chen**), L.Z. (**Lianxin Zhang**), C.Z., Y.X., Z.L., X.S., S.S., J.C., S.M., Y.F., L.Z. (**Liwen Zheng**), L.Z. (**Ling Zhang**); Collection of heart samples from clinical patients: Z.D.; Investigation: Y.H., G.M., Y.Z., K.L., C.X.; Bioinformatics analysis: K.L.; Visualization: Y.H., G.M.; Funding acquisition: X.X., E.D., G.M., K.L.; Project administration: X.X., E.D.; Supervision: X.X., W.H., Y.W.; Writing – original draft: Y.H., G.M.; Writing – review and editing: X.X., Y.H., G.M., A.X. All authors have read and approved the article.

## Funding

This work was supported by the National Natural Science Foundation of China (NSFC) 82270479, U25C2004, and HY2021‐1 to Xunde Xian; the Beijing Natural Science Foundation 7242084 to Xunde Xian; Innovative Drug Research and Development National Science and Technology Major Project to Xunde Xian; Peking University Medicine plus X Pilot Program‐Platform Construction Project 2024YXXLHPT010 to Xunde Xian; the CAMS Innovation Fund for Medical Sciences 2021‐I2M‐5‐003 to Erdan Dong; the NSFC 8240033347 to Guolin Miao; China Postdoctoral Science Foundation 2025M771442 and NSFC 82501040 to Kaikai Lu.

## Conflicts of Interest

The authors declare no conflicts of interest.

## Supporting information




**Supporting File 1**: advs77196‐sup‐0001‐SuppMat.docx.

**Supporting File 2**: advs77196‐sup‐0002‐SuppMat.pdf.

## Data Availability

The RNA sequencing data were deposited in the NCBI's Gene Expression Omnibus database (PRJNA1404915). Values for all data points are available in the Supporting Data Values file. Additional requests for resources or reagents should be directed to Prof. Xunde Xian at xianxunde@bjmu.edu.cn.
