# Supplementary material for: SR‐A3 Promotes USP18‐Mediated deISGylation of STING to Protect Against Myocardial Ischemia‐Reperfusion Injury
Source: Adv Sci (Weinh). 2026 Aug 17;13(62):e77196. doi: 10.1002/advs.77196 (PMC13479855; doi:10.1002/advs.77196)
Supplement: Supplementary file 1 [file ADVS-13-e77196-s001.pdf]

Fig 1d

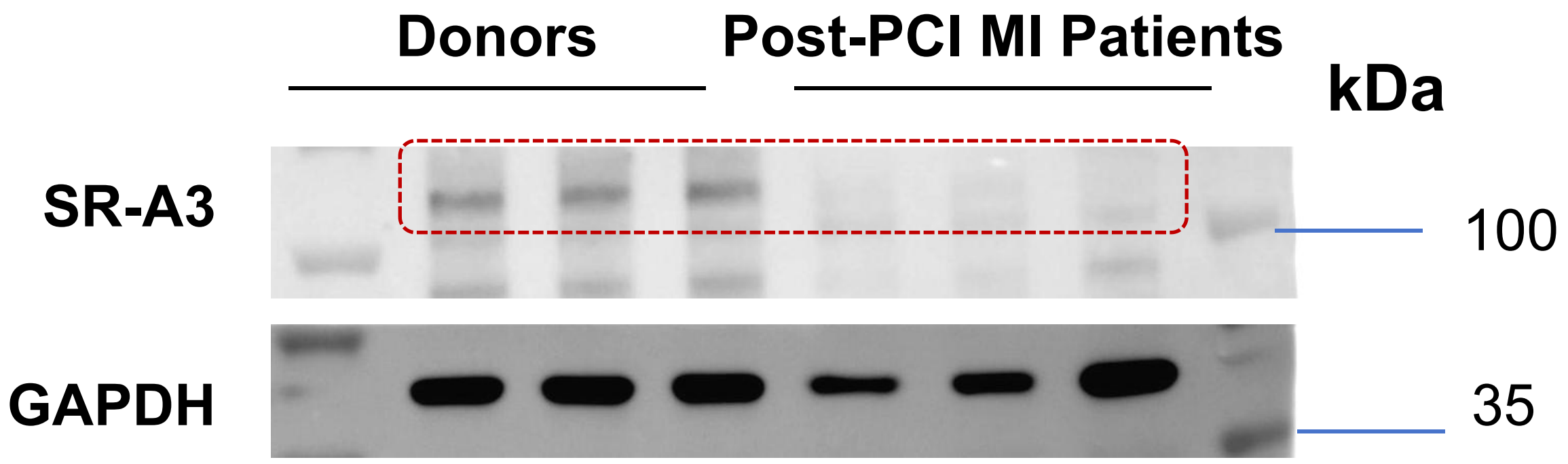

Donors
Post-PCI MI Patients
kDa
SR-A3
100
GAPDH
35

Fig 1i

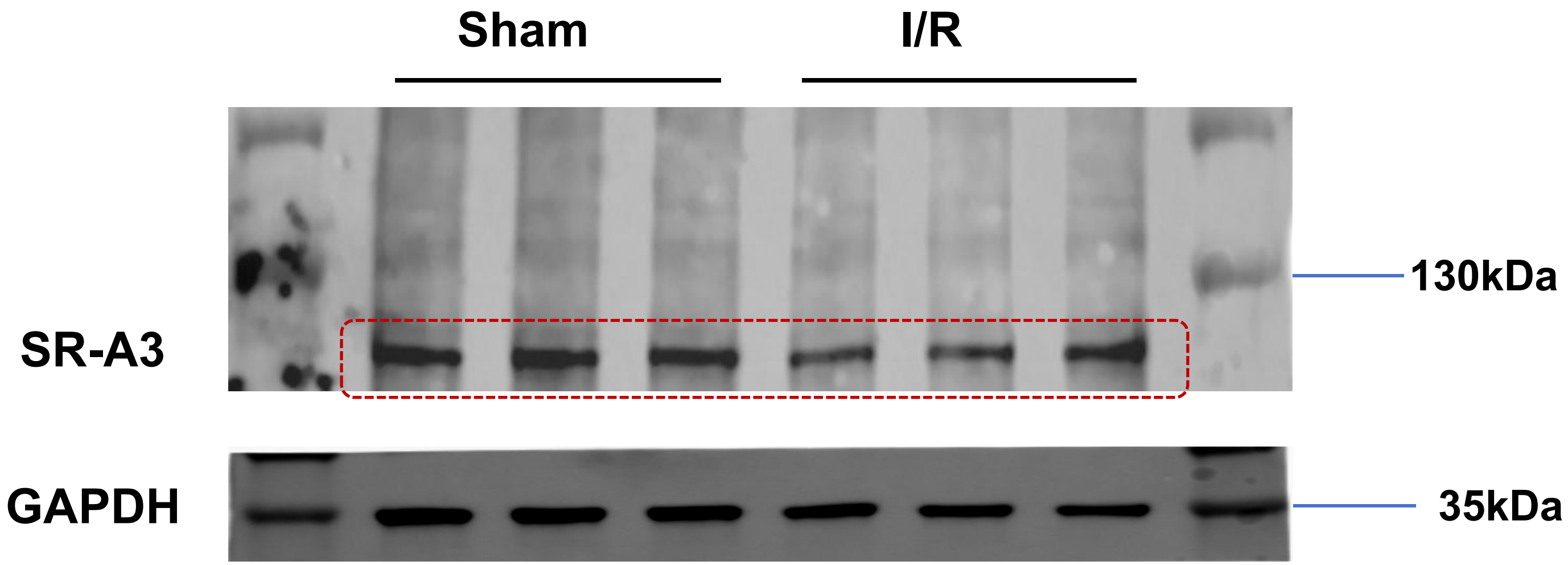

Sham
I/R
SR-A3
130kDa
GAPDH
35kDa

Fig 3c

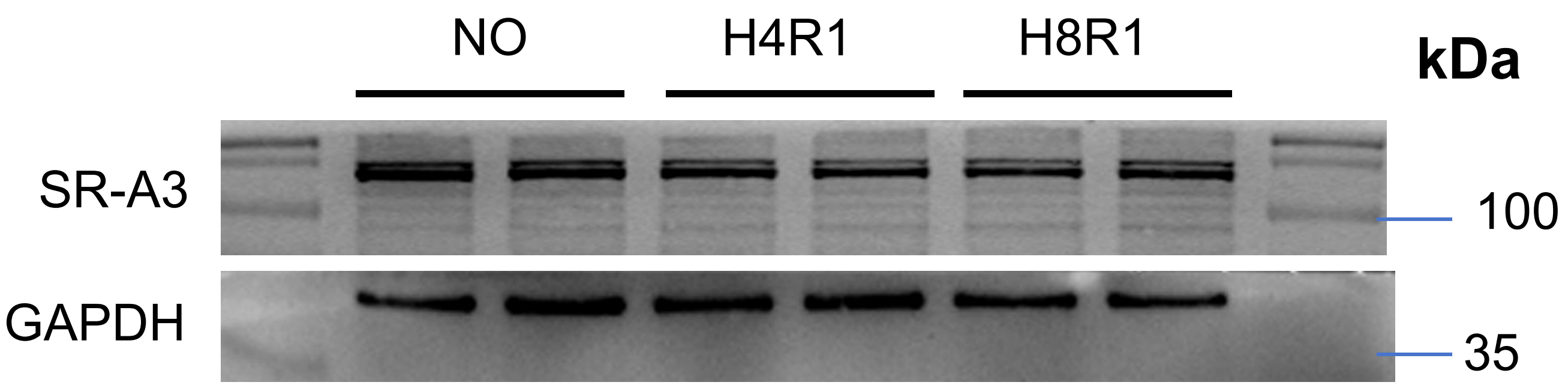

Fig 3f

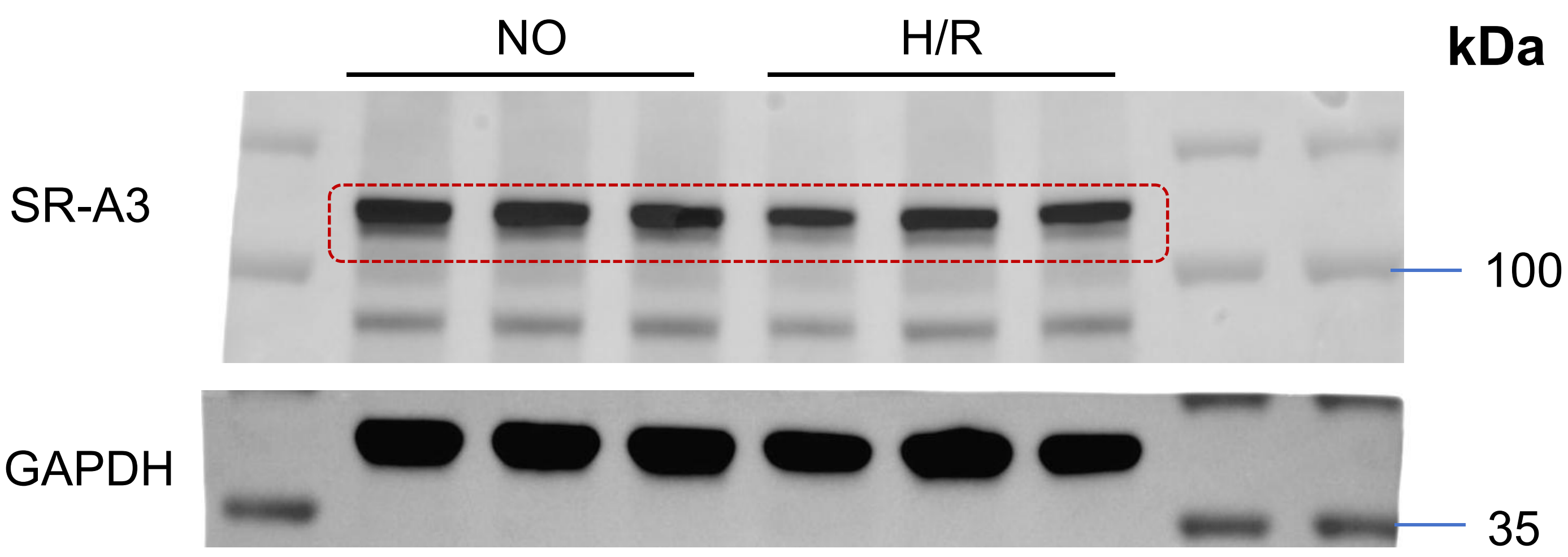

Fig 4d

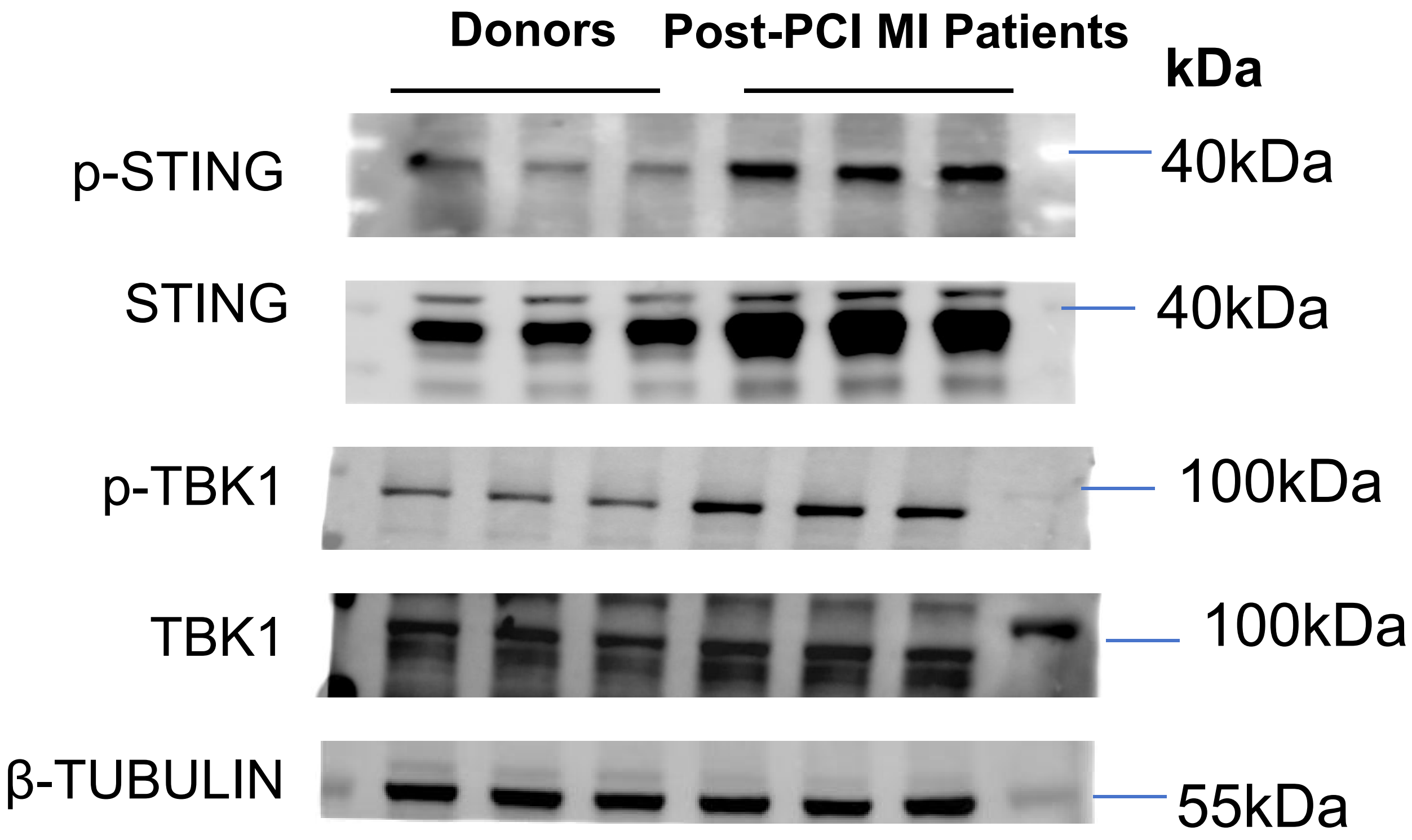
Donors
Post-PCI MI Patients
kDa
p-STING
40kDa
STING
40kDa
p-TBK1
100kDa
TBK1
100kDa
β-TUBULIN
55kDa

Fig 4f

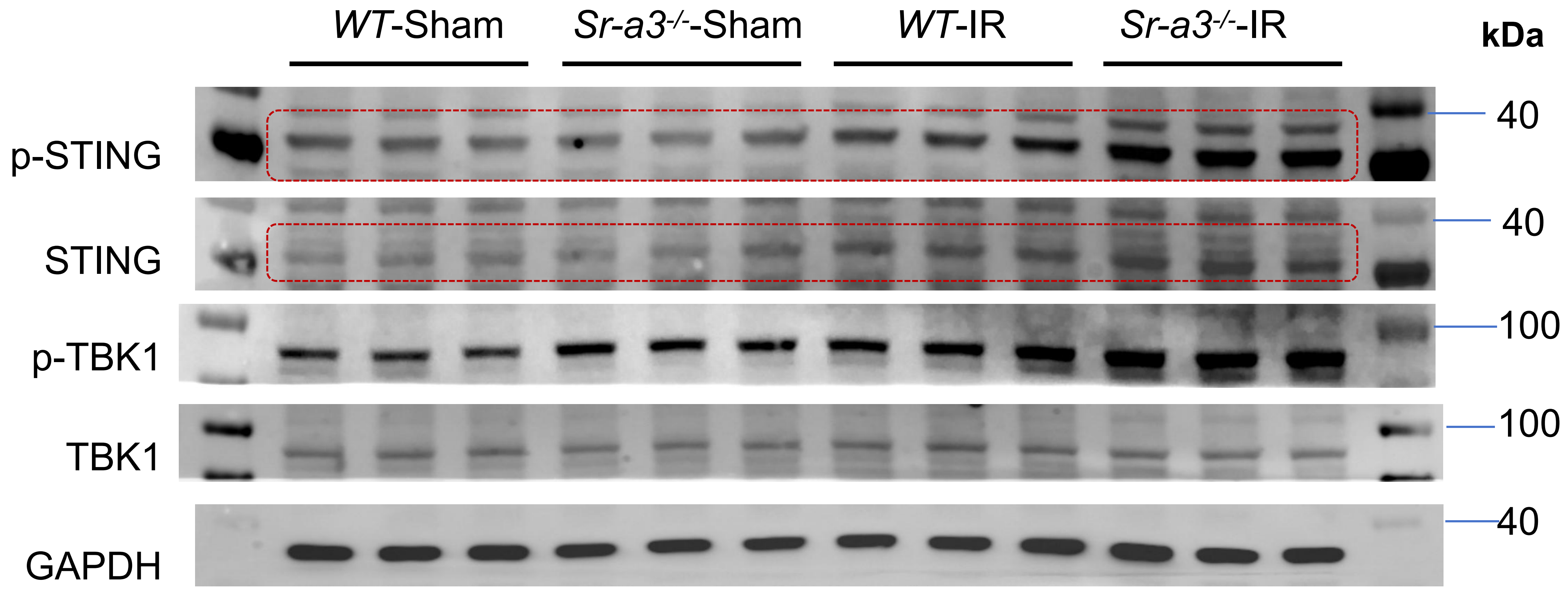

WT-Sham
Sr-a3-/--Sham
WT-IR
Sr-a3-/--IR
kDa
p-STING
40
STING
40
p-TBK1
100
TBK1
100
GAPDH
40

Fig 4h

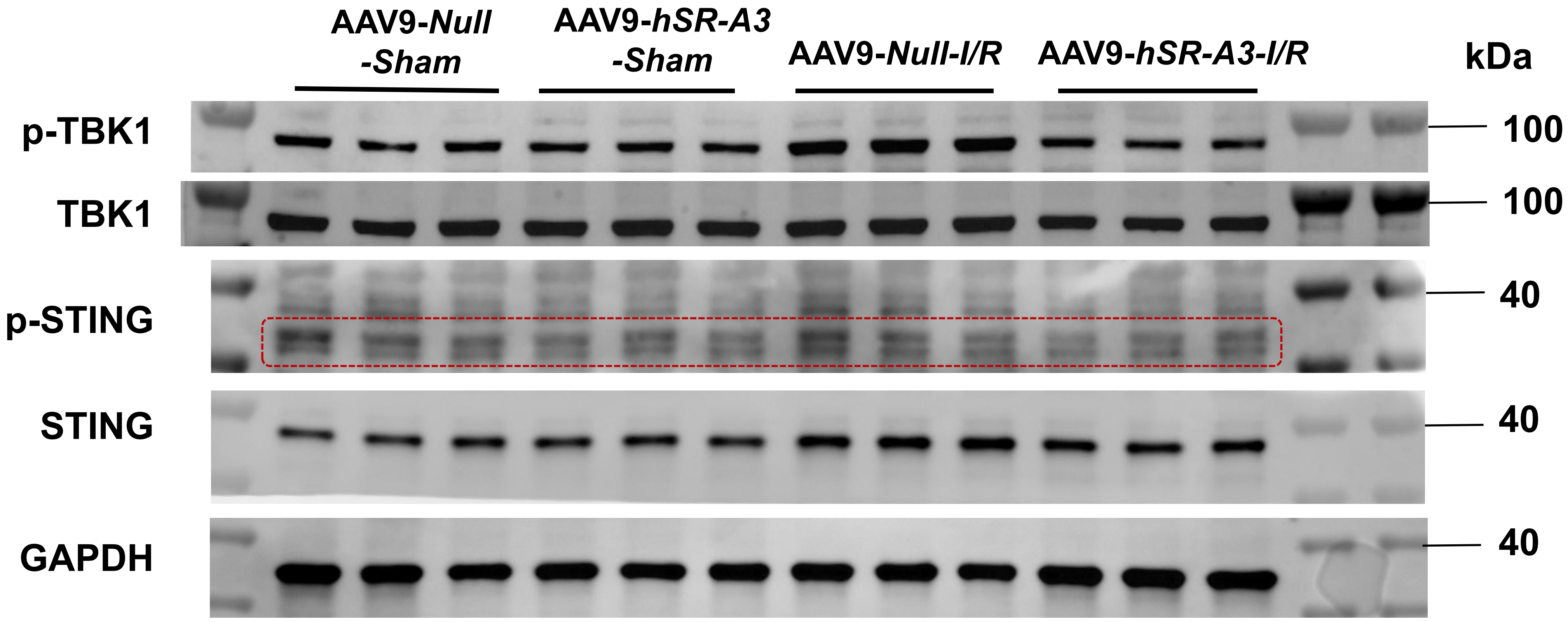

AAV9-Null
-Sham
AAV9-hSR-A3
-Sham
AAV9-Null-I/R
AAV9-hSR-A3-I/R
kDa
p-TBK1
100
TBK1
100
p-STING
40
STING
40
GAPDH
40

Fig 5a

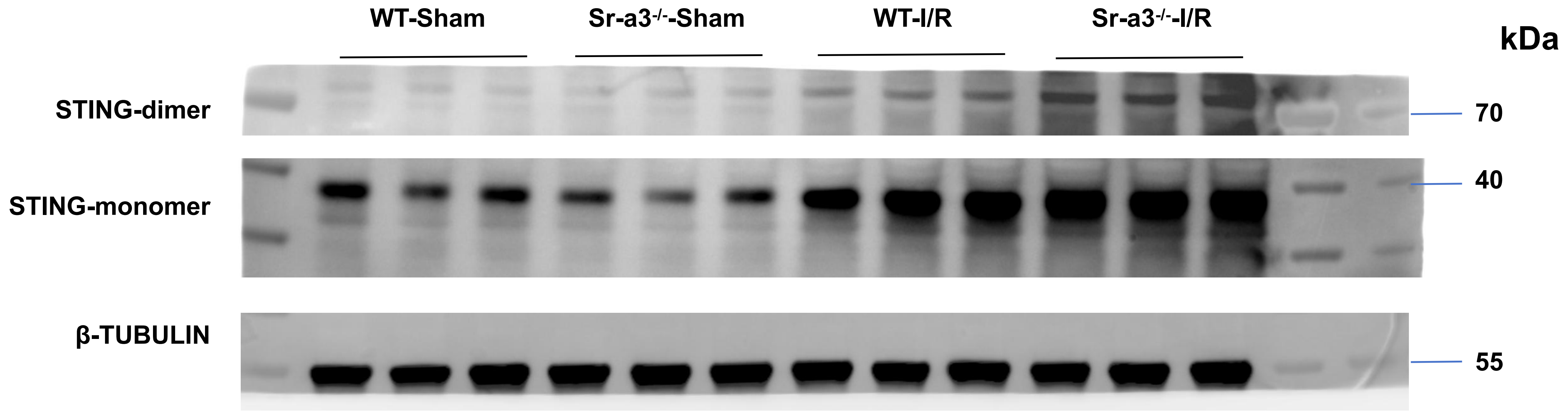
WT-Sham
Sr-a3-/--Sham
WT-I/R
Sr-a3-/--I/R
kDa
STING-dimer
70
STING-monomer
40
β-TUBULIN
55

Fig 5b

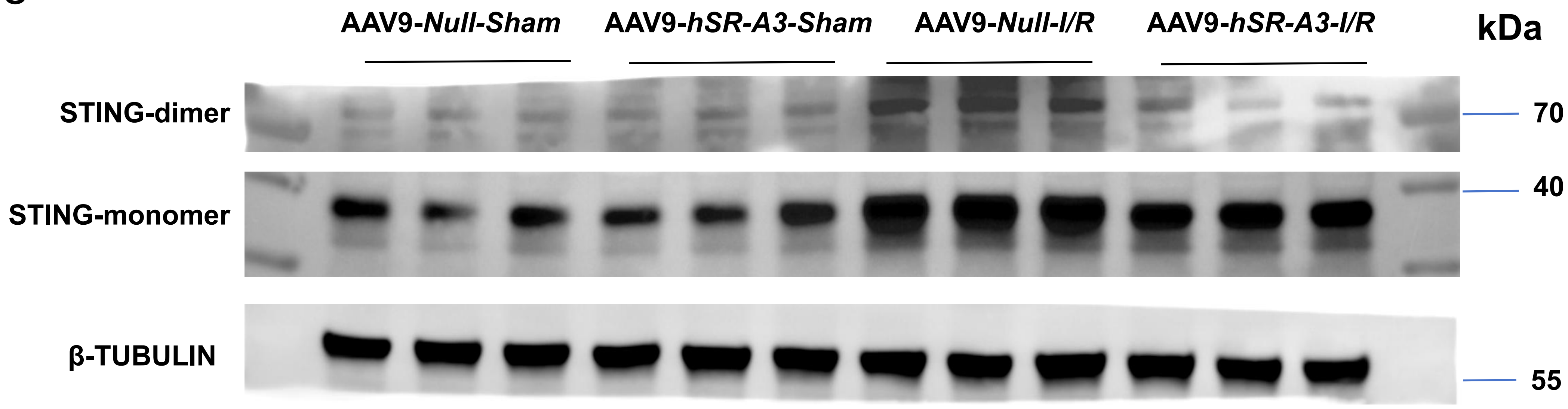
AAV9-Null-Sham
AAV9-hSR-A3-Sham
AAV9-Null-I/R
AAV9-hSR-A3-I/R
kDa
STING-dimer
70
STING-monomer
40
β-TUBULIN
55

Fig 5c

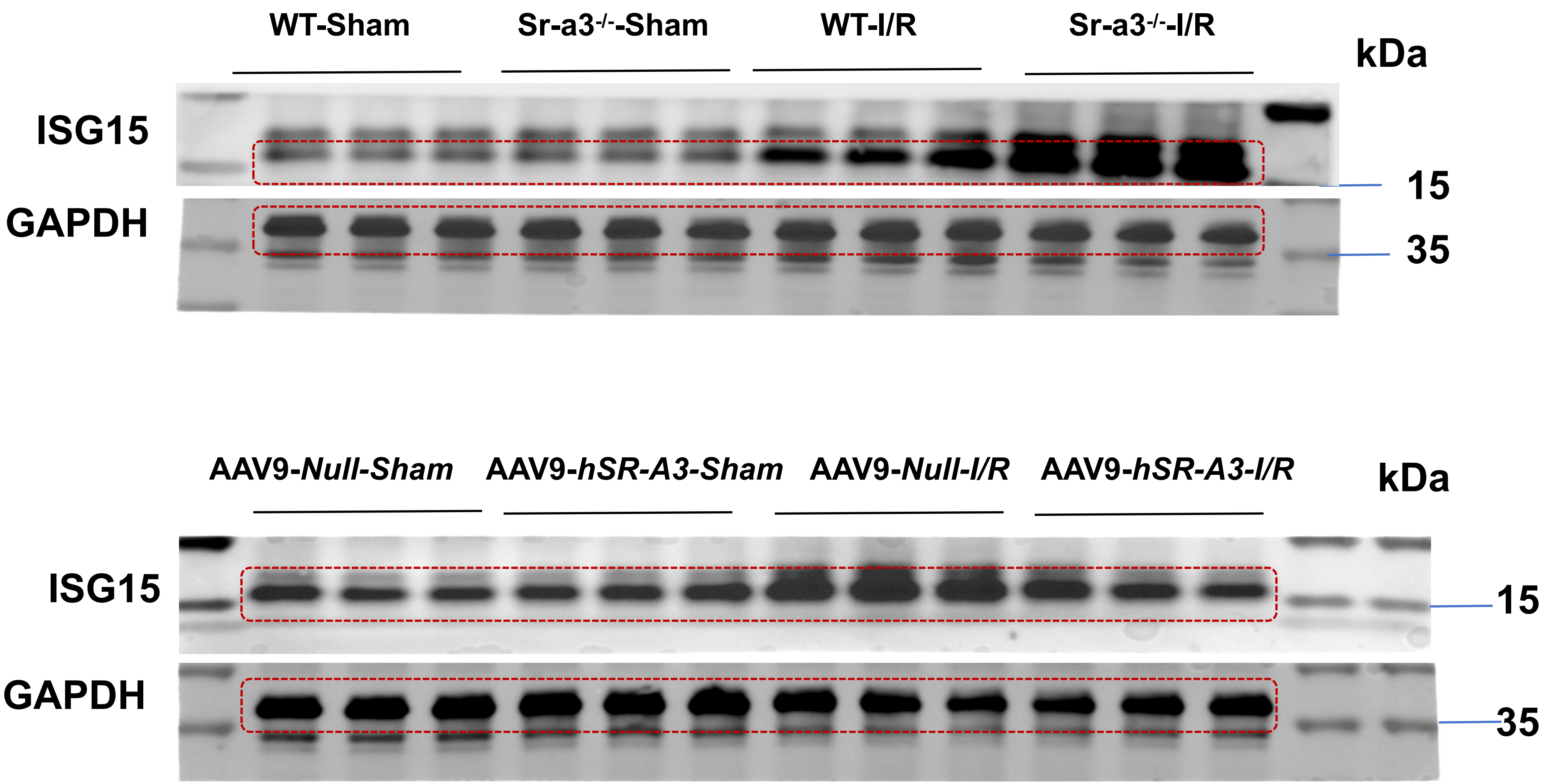
WT-Sham
Sr-a3-/--Sham
WT-I/R
Sr-a3-/--I/R
kDa
ISG15
GAPDH
15
35
AAV9-Null-Sham
AAV9-hSR-A3-Sham
AAV9-Null-I/R
AAV9-hSR-A3-I/R
kDa
ISG15
GAPDH
15
35

Fig 5e

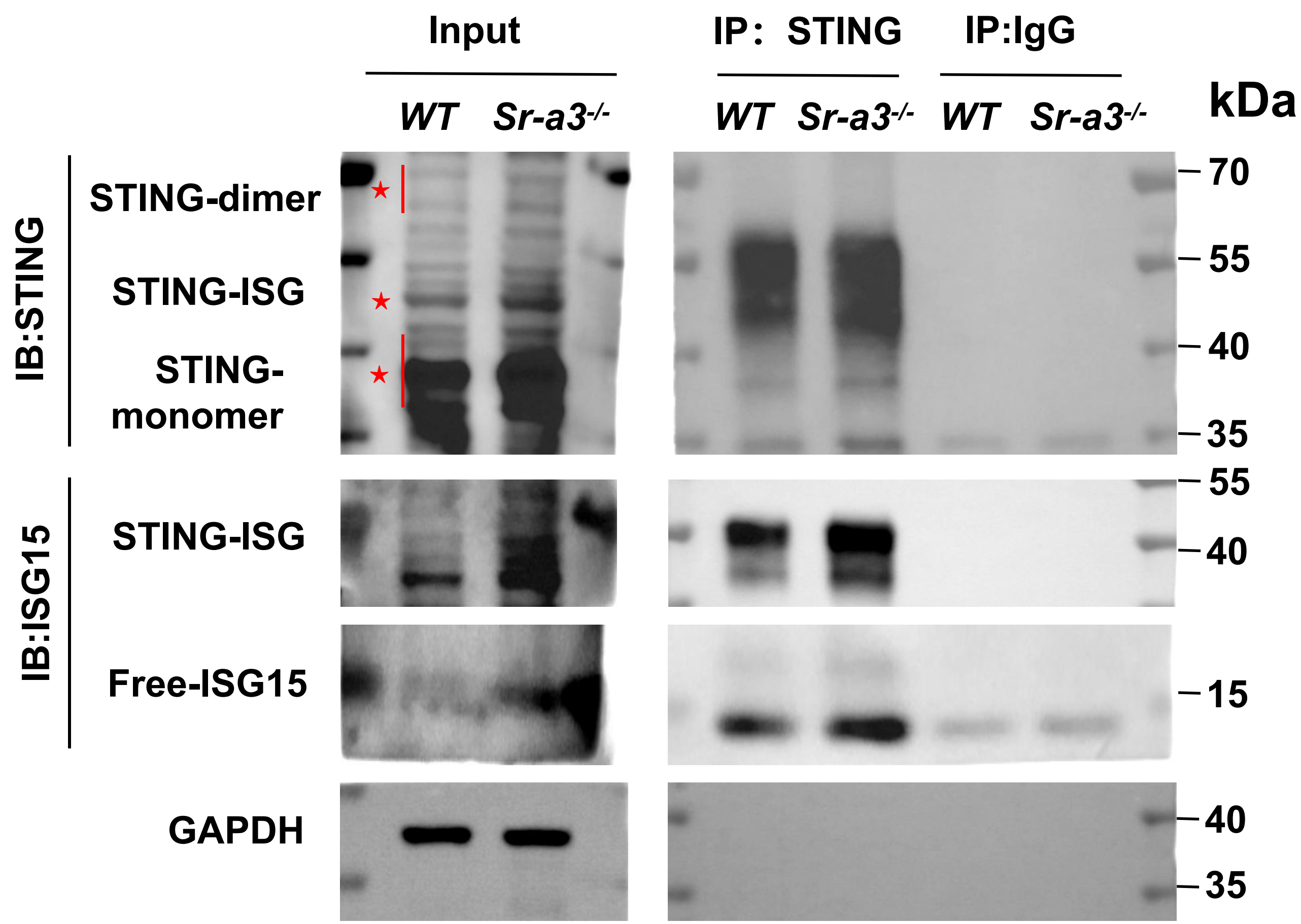
Input
IP： STING
IP:IgG
WT
Sr-a3-/-
WT
Sr-a3-/-
WT
Sr-a3-/-
kDa
IB:STING
STING-dimer
STING-ISG
STING-
monomer
70
55
40
35
IB:ISG15
STING-ISG
Free-ISG15
55
40
15
GAPDH
40
35

Fig 5f

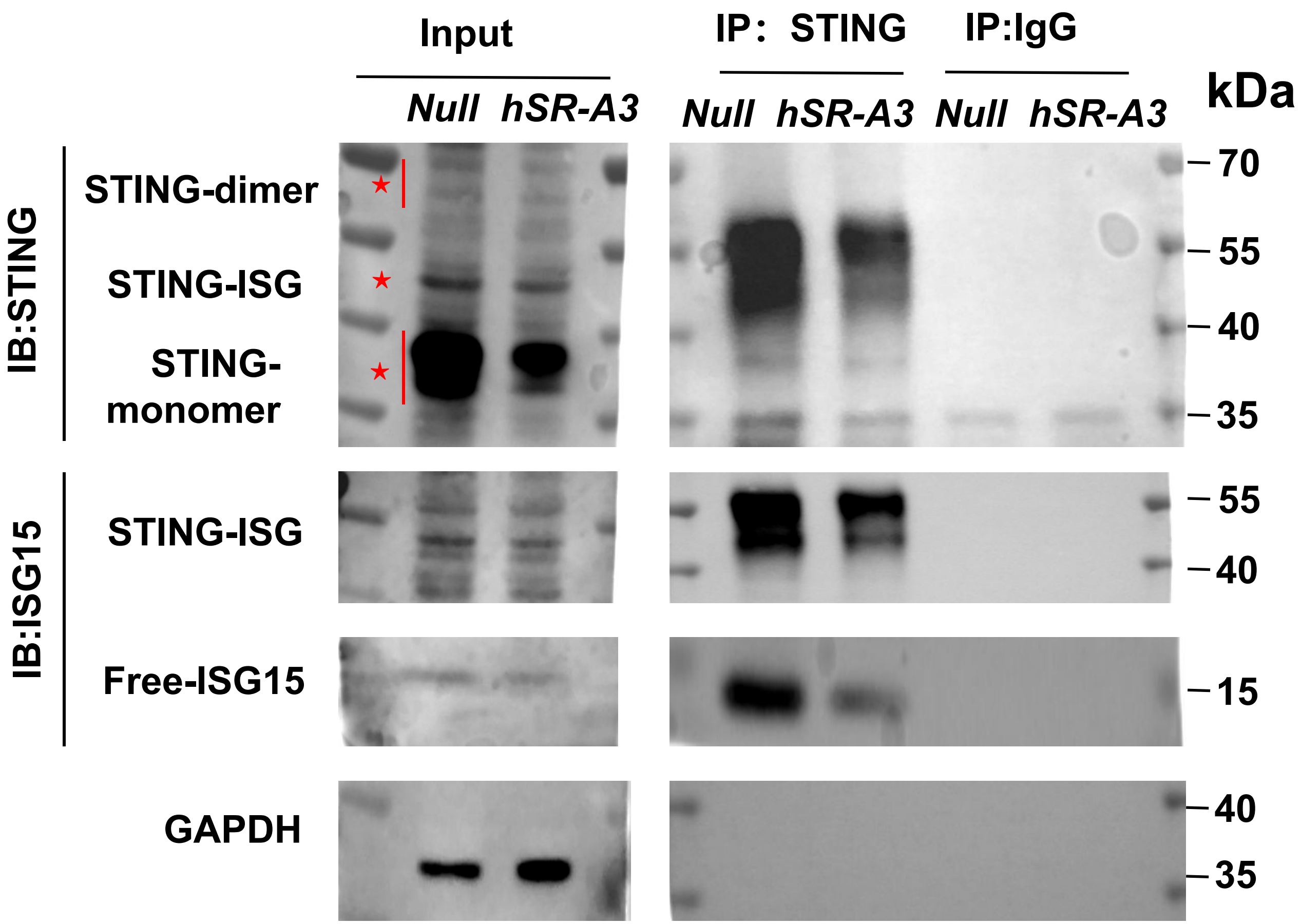

Input
IP: STING
IP:IgG
Null
hSR-A3
Null
hSR-A3
Null
hSR-A3
kDa
IB:STING
STING-dimer
STING-ISG
STING-monomer
70
55
40
35
IB:ISG15
STING-ISG
55
40
Free-ISG15
15
GAPDH
40
35

Fig 5g

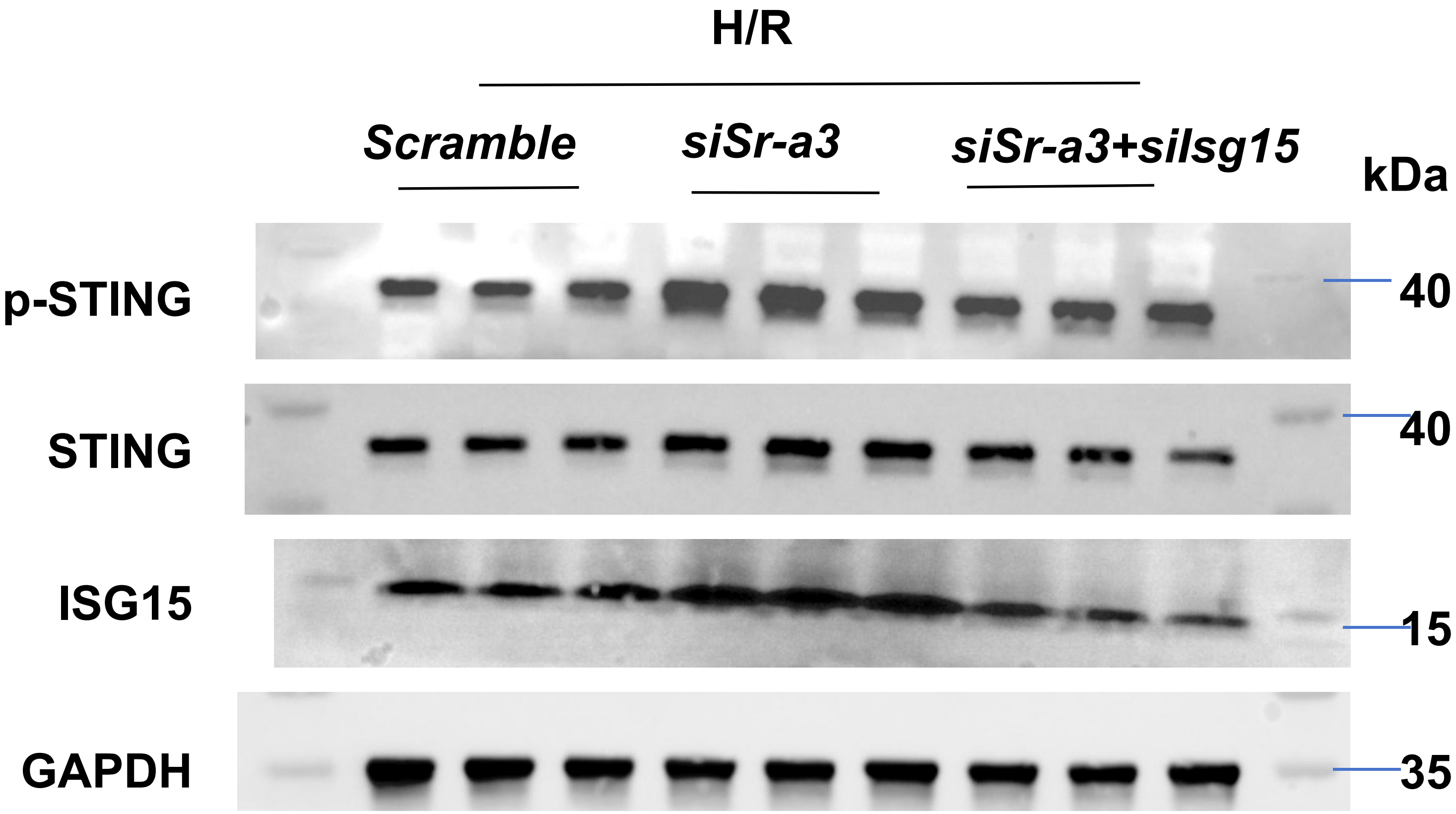

H/R
Scramble
siSr-a3
siSr-a3+siIsg15
kDa
p-STING
STING
ISG15
GAPDH
40
40
15
35

Fig 5h

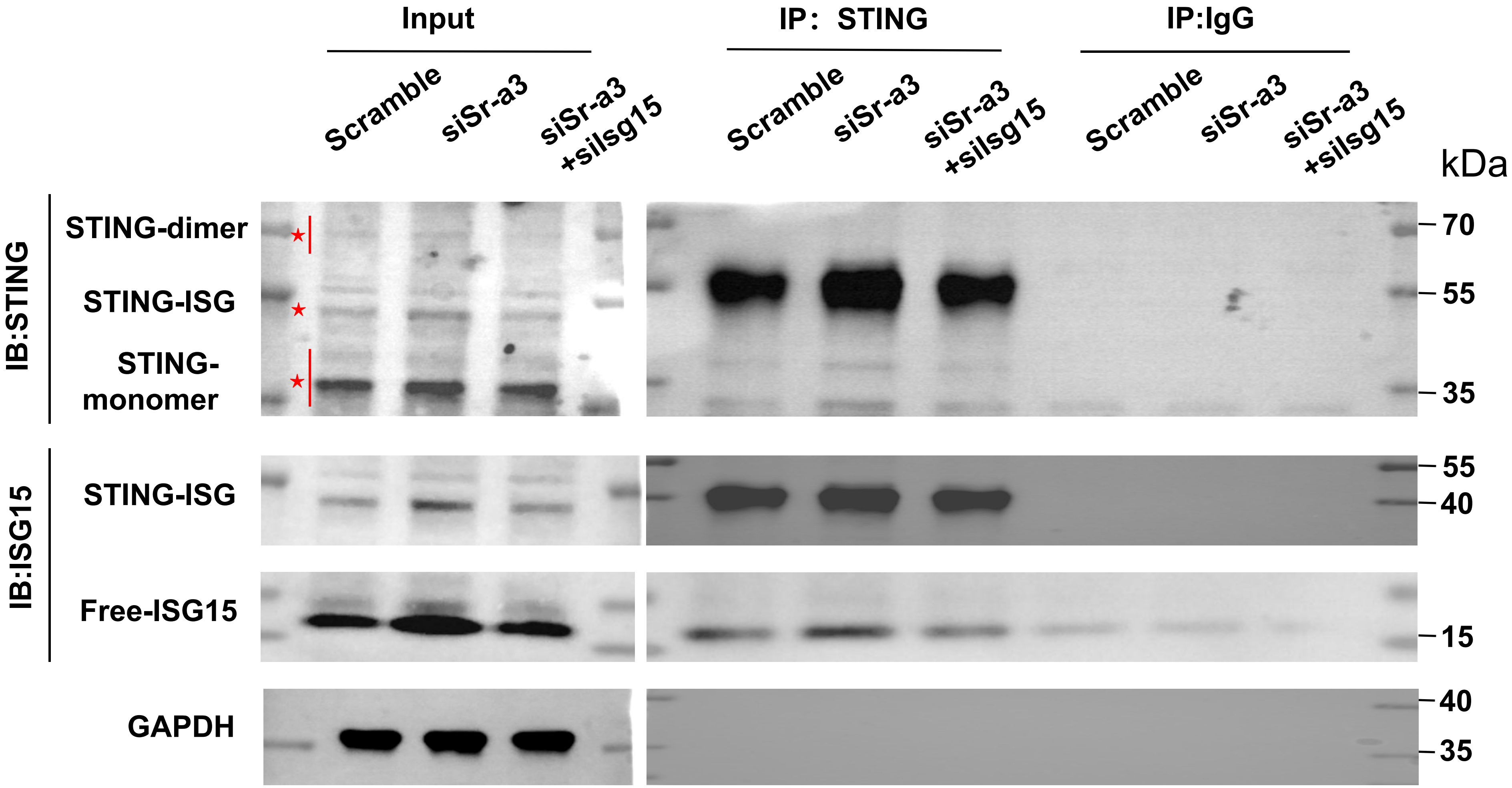
Input
IP: STING
IP:IgG
Scramble
siSr-a3
siSr-a3 +siIsg15
Scramble
siSr-a3
siSr-a3 +siIsg15
Scramble
siSr-a3
siSr-a3 +siIsg15
kDa
IB:STING
STING-dimer
STING-ISG
STING-monomer
70
55
35
IB:ISG15
STING-ISG
Free-ISG15
55
40
15
GAPDH
40
35

Fig 6c

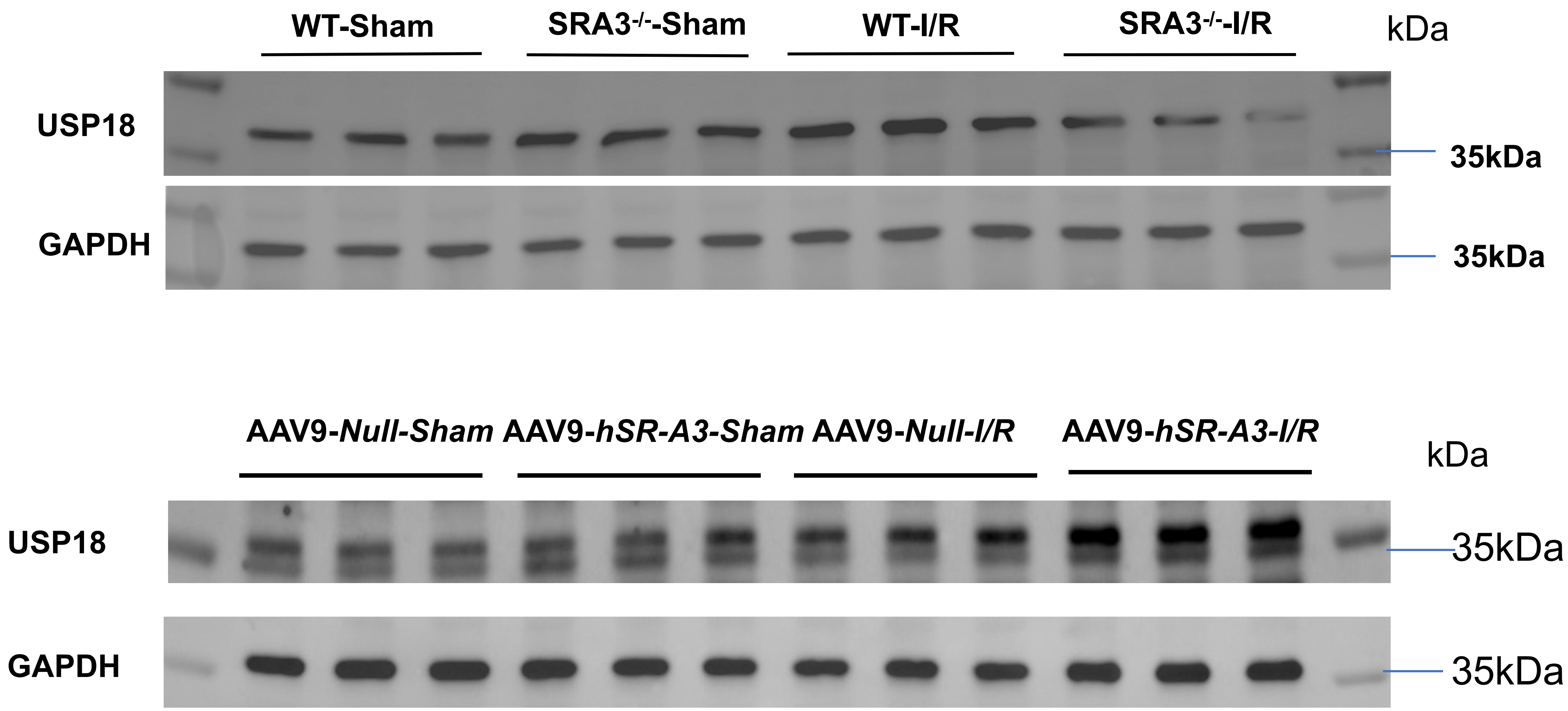
WT-Sham
$SRA3^{-/-}$-Sham
WT-I/R
$SRA3^{-/-}$-I/R
kDa
USP18
35kDa
GAPDH
35kDa
AAV9-*Null-Sham*
AAV9-*hSR-A3-Sham*
AAV9-*Null-I/R*
AAV9-*hSR-A3-I/R*
kDa
USP18
35kDa
GAPDH
35kDa

Fig 6e

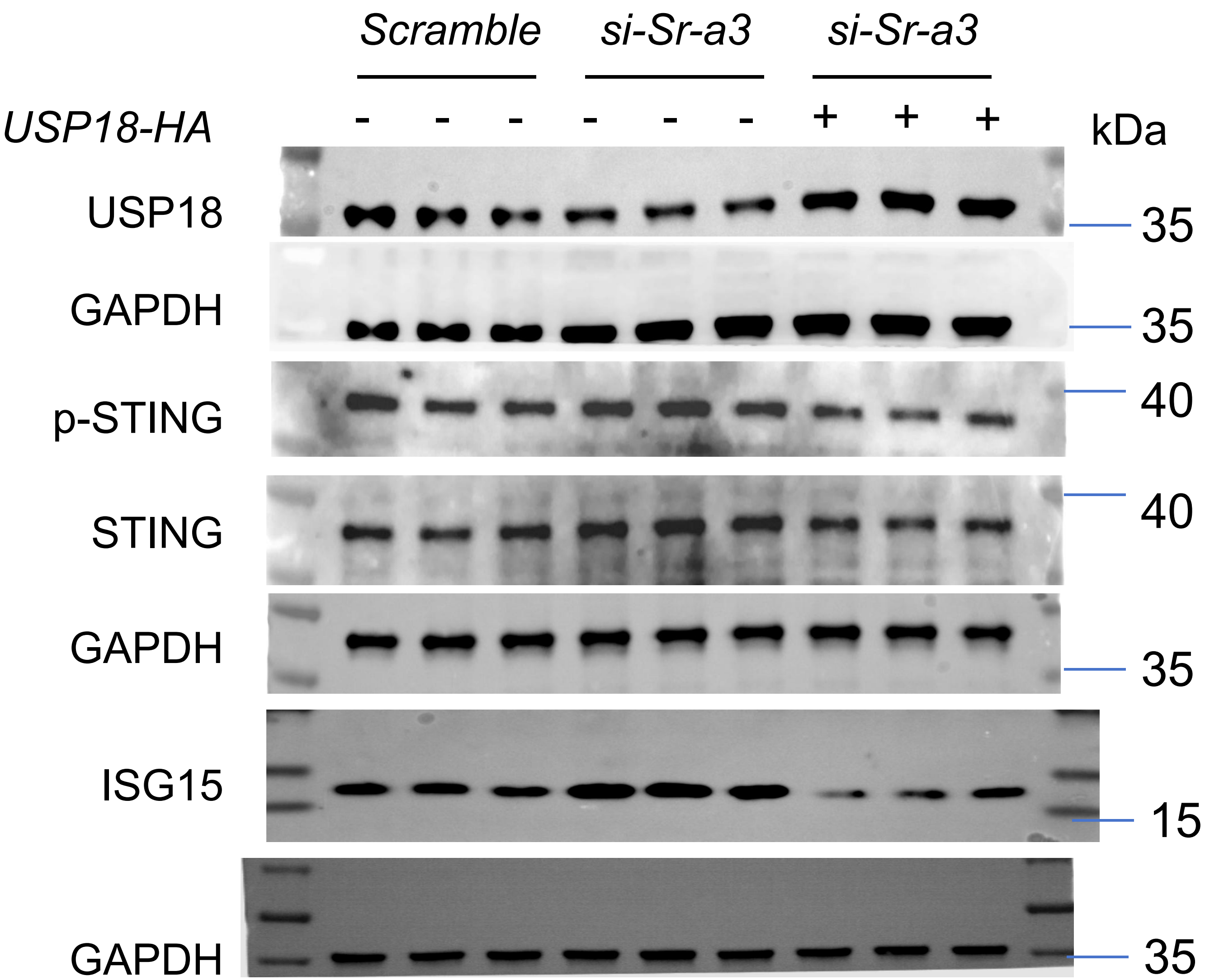
Scramble
si-Sr-a3
si-Sr-a3
USP18-HA
- - - - - - + + +
kDa
USP18
35
GAPDH
35
p-STING
40
STING
40
GAPDH
35
ISG15
15
GAPDH
35

Fig 6h

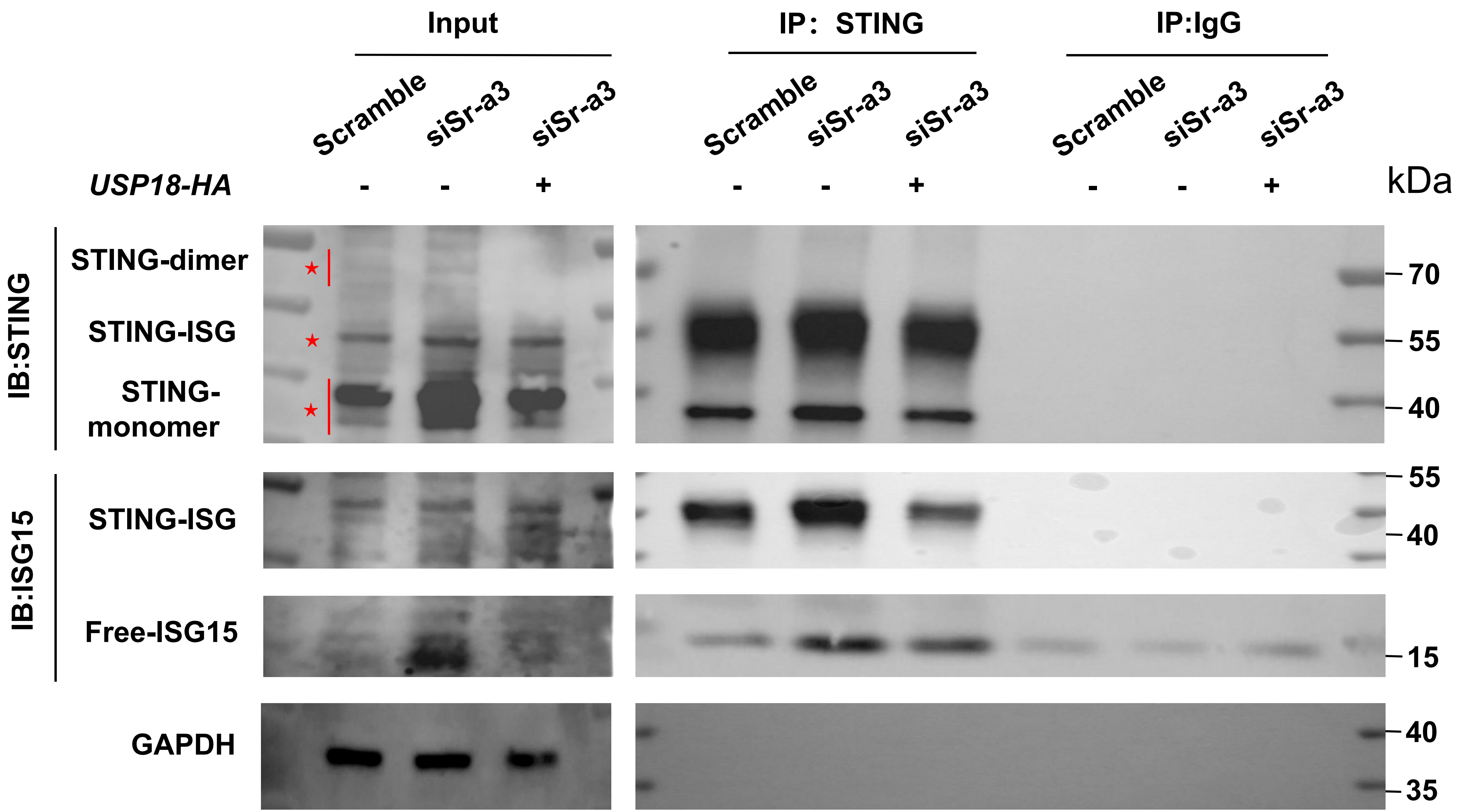

Input
IP: STING
IP:IgG
Scramble
siSr-a3
siSr-a3
Scramble
siSr-a3
siSr-a3
Scramble
siSr-a3
siSr-a3
USP18-HA
- - +
- - +
- - +
kDa
IB:STING
STING-dimer
STING-ISG
STING-monomer
70
55
40
IB:ISG15
STING-ISG
Free-ISG15
55
40
15
GAPDH
40
35

Fig 7b

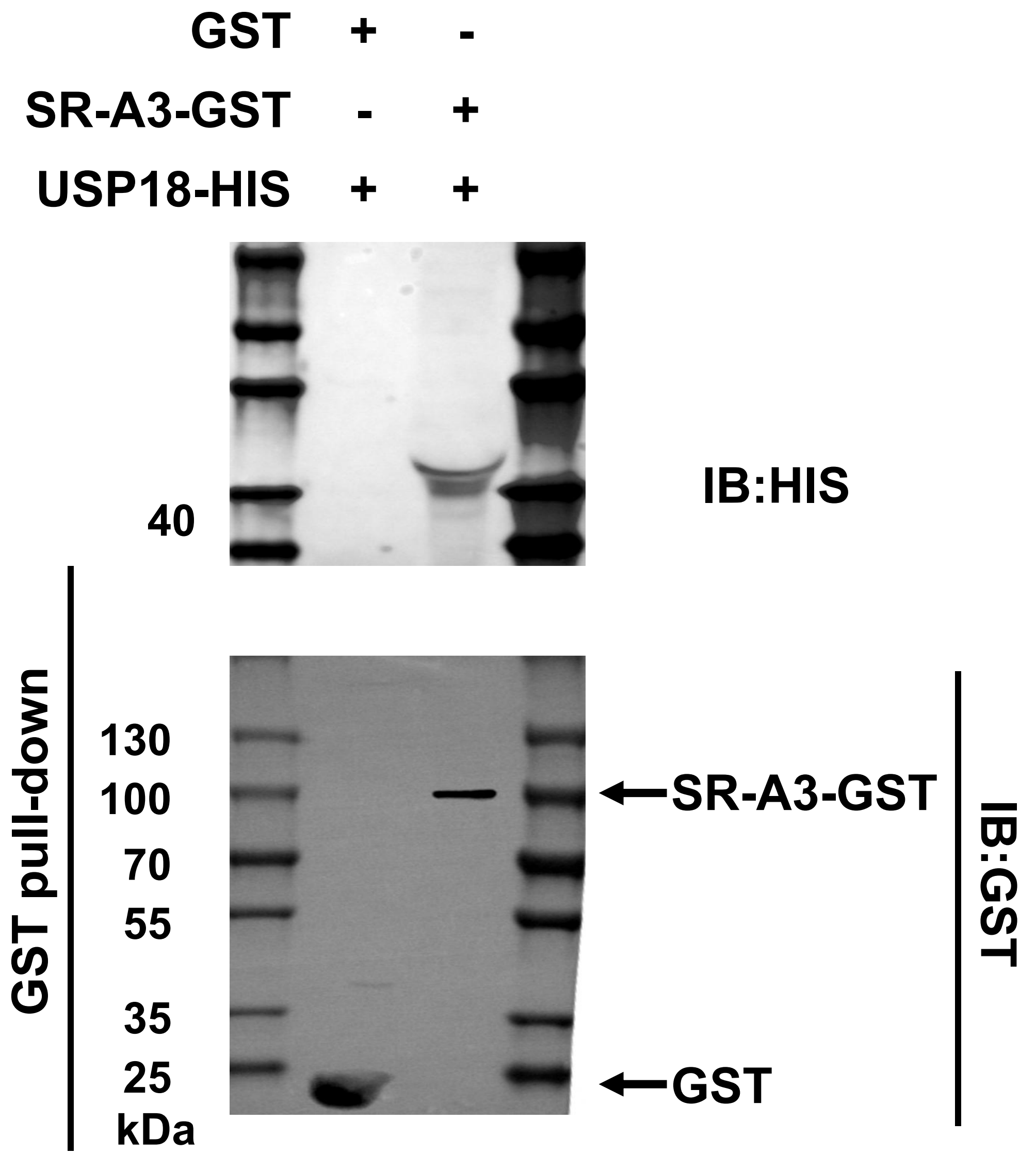

GST + -
SR-A3-GST - +
USP18-HIS + +
40
IB:HIS
GST pull-down
130
100
70
55
35
25
kDa
SR-A3-GST
GST
IB:GST

Fig 7d

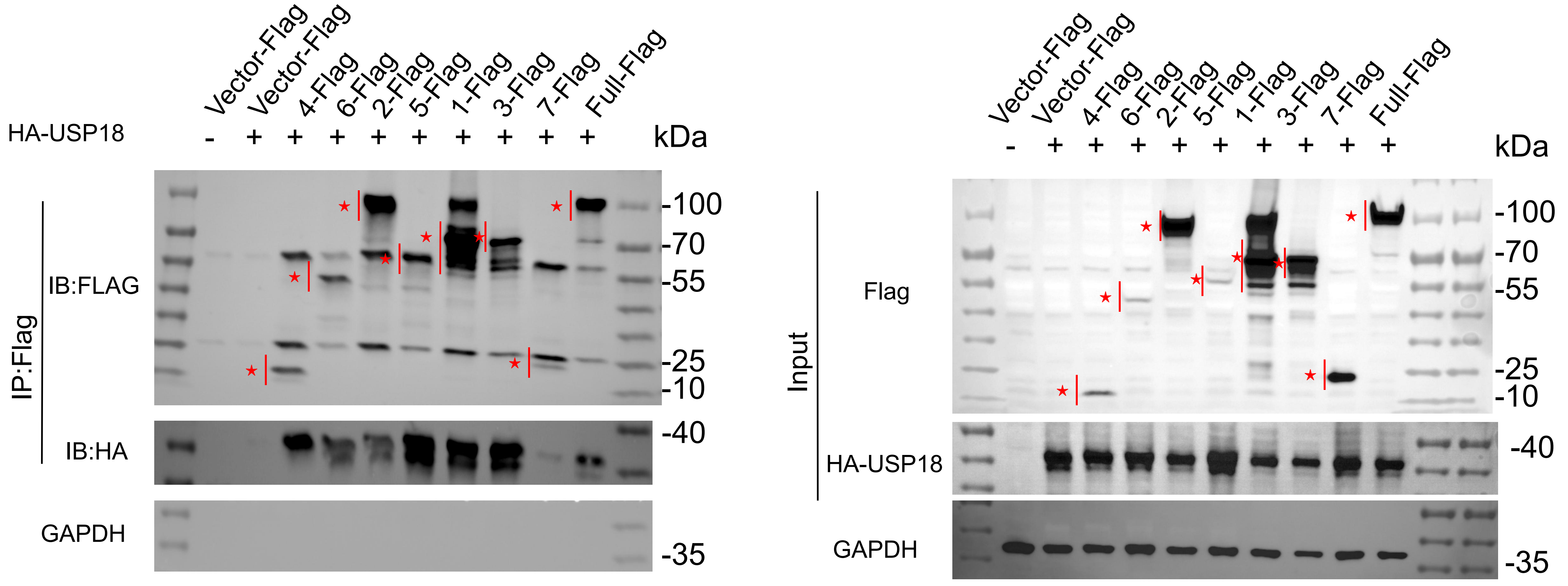
Vector-Flag
Vector-Flag
4-Flag
6-Flag
2-Flag
5-Flag
1-Flag
3-Flag
7-Flag
Full-Flag
HA-USP18
- + + + + + + + + +
kDa
-100
-70
-55
-25
-10
-40
-35
IP:Flag
IB:FLAG
IB:HA
GAPDH
Input
Flag
HA-USP18
GAPDH

Fig 7e

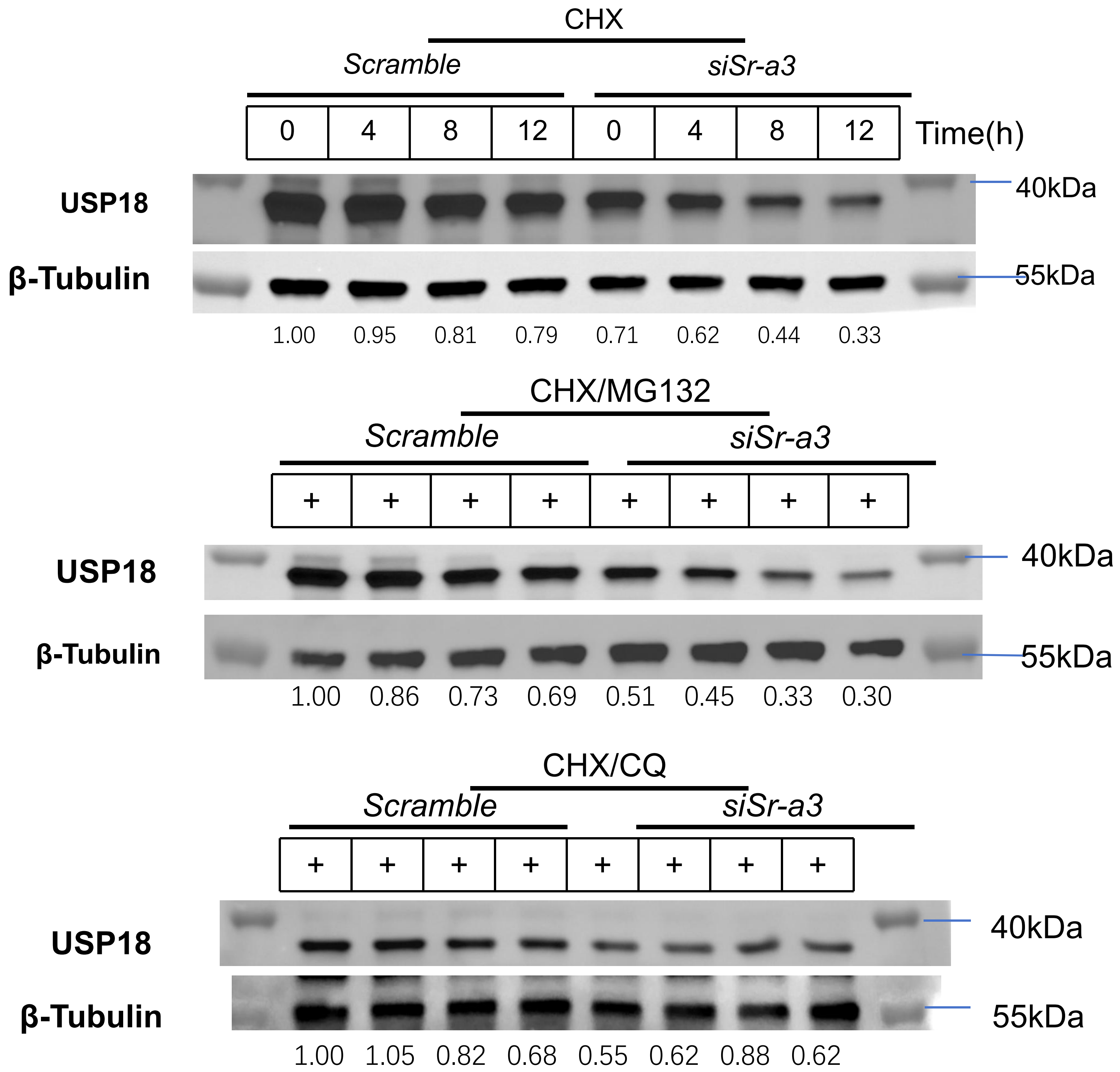
CHX
Scramble
siSr-a3
0
4
8
12
0
4
8
12
Time(h)
USP18
β-Tubulin
40kDa
55kDa
1.00
0.95
0.81
0.79
0.71
0.62
0.44
0.33
CHX/MG132
Scramble
siSr-a3
+ + + + + + + +
USP18
β-Tubulin
40kDa
55kDa
1.00
0.86
0.73
0.69
0.51
0.45
0.33
0.30
CHX/CQ
Scramble
siSr-a3
+ + + + + + + +
USP18
β-Tubulin
40kDa
55kDa
1.00
1.05
0.82
0.68
0.55
0.62
0.88
0.62

Fig 8b

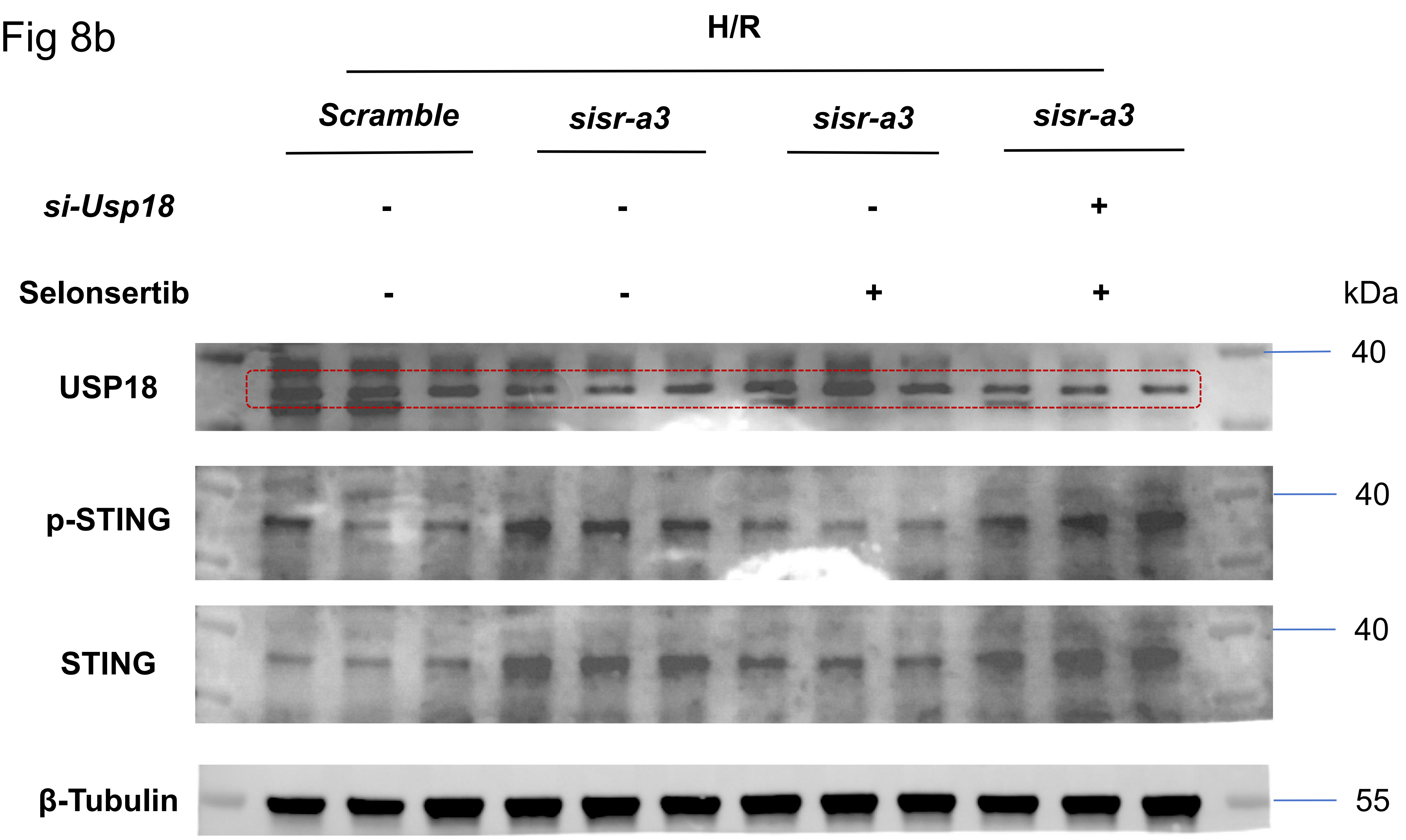
H/R
Scramble
sisr-a3
sisr-a3
sisr-a3
si-Usp18
-
-
-
+
Selonsertib
-
-
+
+
kDa
USP18
40
p-STING
40
STING
40
β-Tubulin
55

Fig 8c

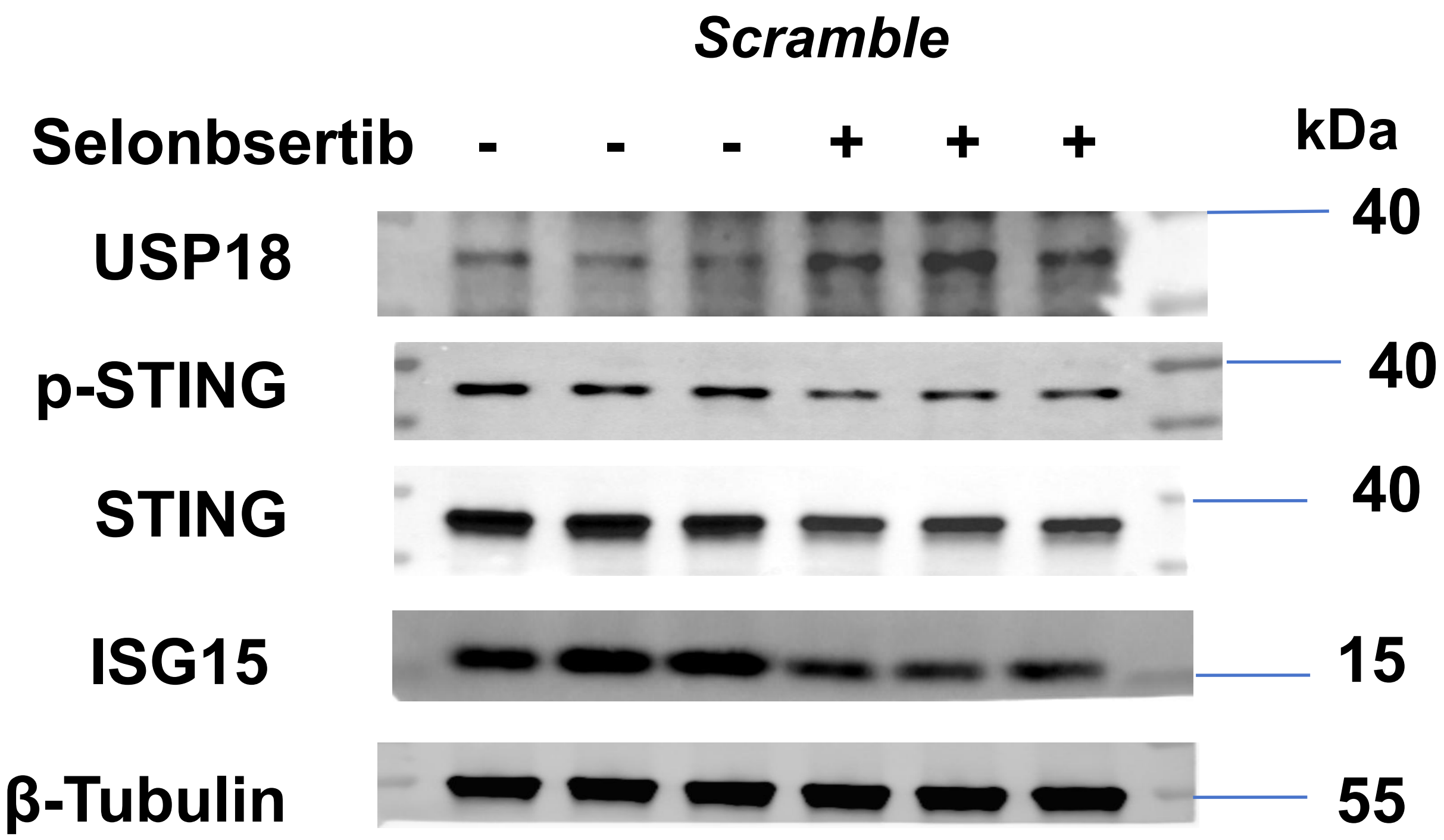
Scramble
Selonbsertib - - - + + +
kDa
USP18
40
p-STING
40
STING
40
ISG15
15
β-Tubulin
55

Fig 8d

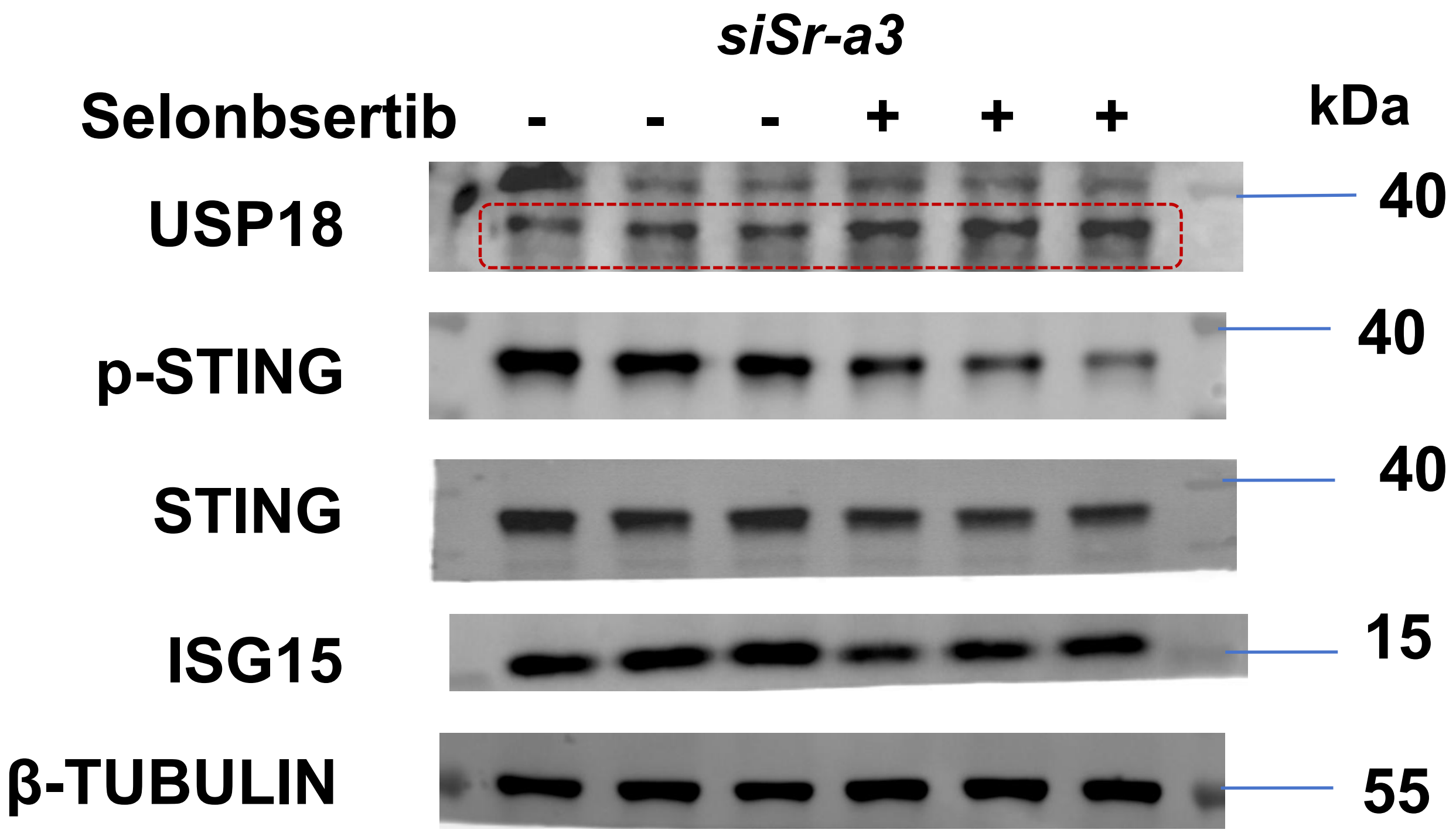
siSr-a3
Selonbsertib - - - + + +
kDa
USP18
40
p-STING
40
STING
40
ISG15
15
β-TUBULIN
55

Fig 8e

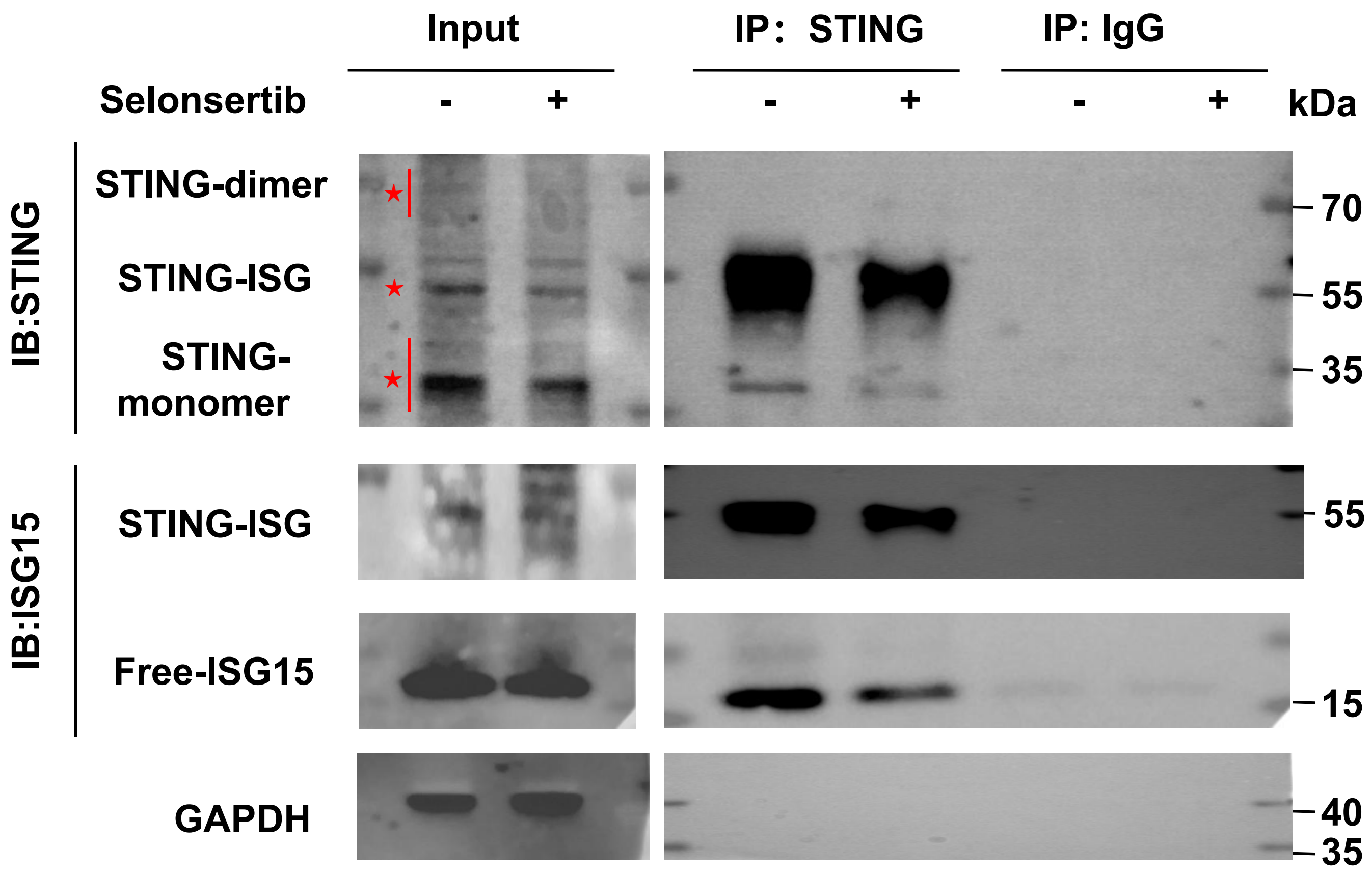
Input
IP: STING
IP: IgG
Selonsertib
-
+
-
+
-
+
kDa
IB:STING
STING-dimer
STING-ISG
STING-
monomer
70
55
35
IB:ISG15
STING-ISG
55
Free-ISG15
15
GAPDH
40
35

Fig 10i

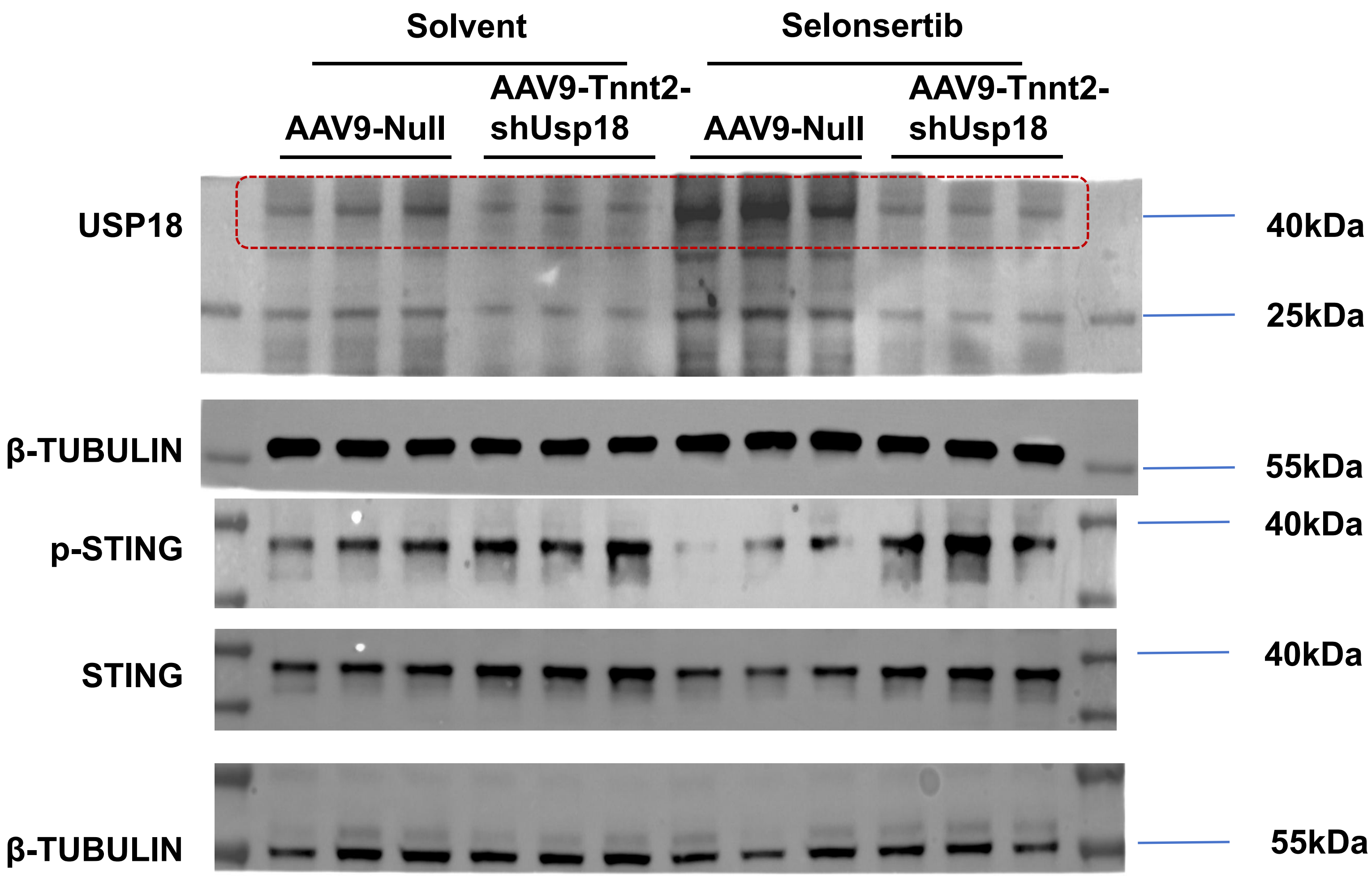

Solvent
Selonsertib
AAV9-Null
AAV9-Tnnt2-shUsp18
AAV9-Null
AAV9-Tnnt2-shUsp18
USP18
40kDa
25kDa
β-TUBULIN
55kDa
p-STING
40kDa
STING
40kDa
β-TUBULIN
55kDa

Fig S1b

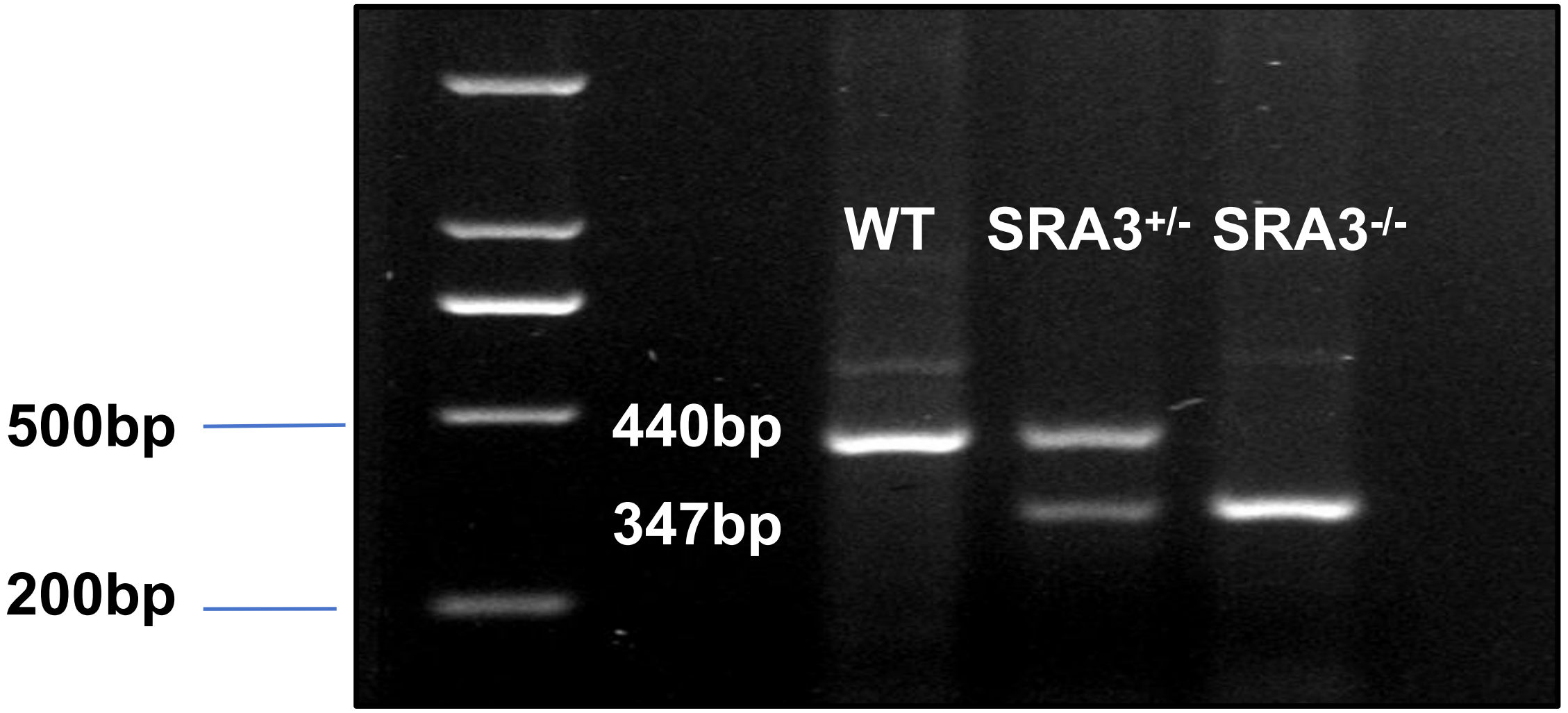

Fig S3b

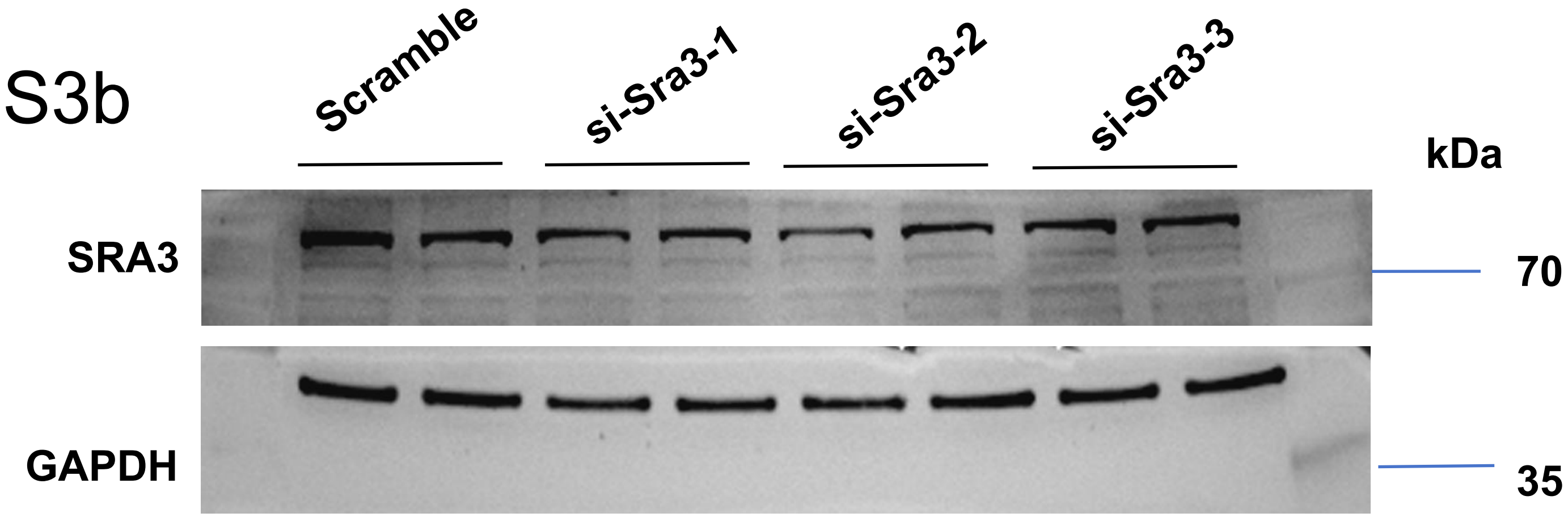

Fig S3j

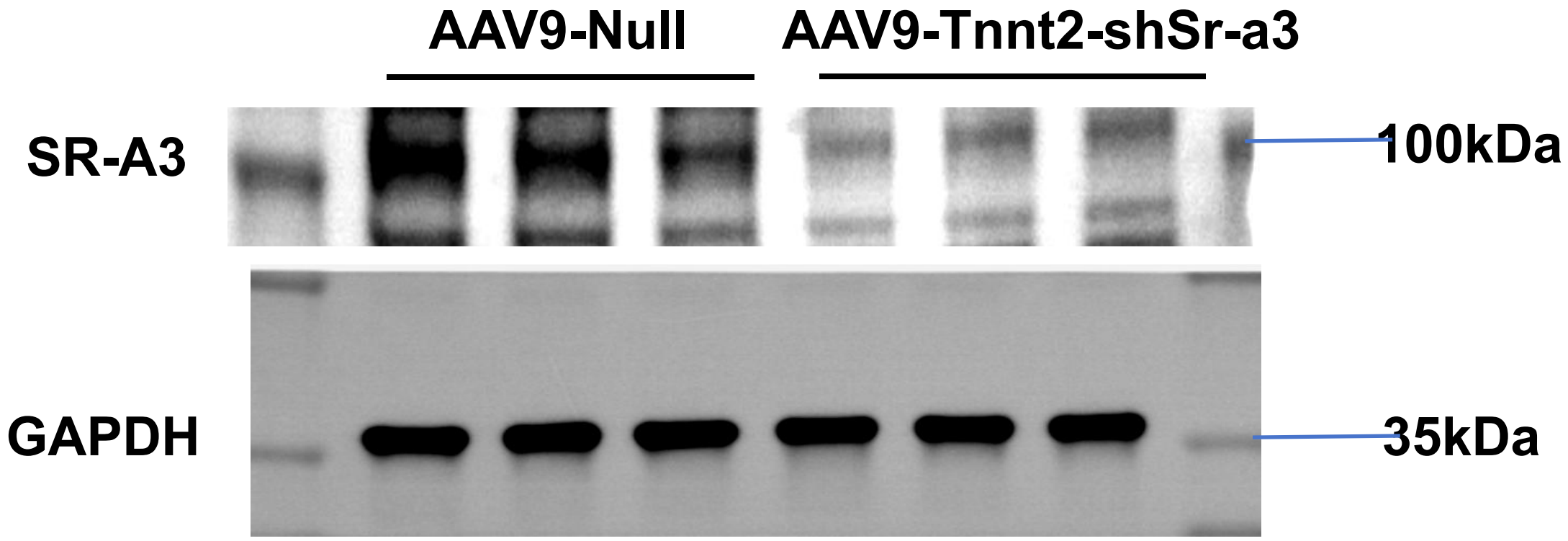

Fig S3f

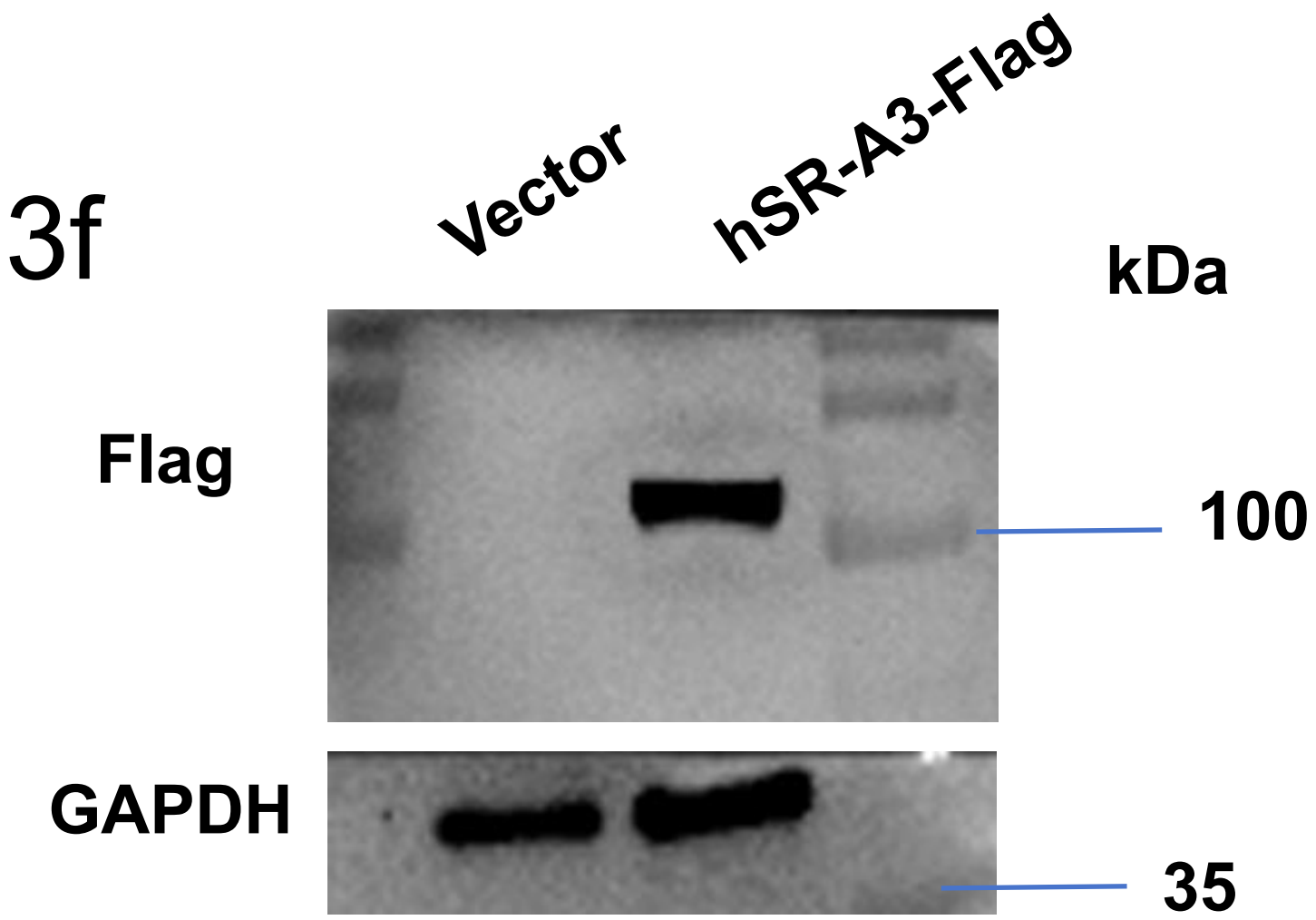

Fig S3l

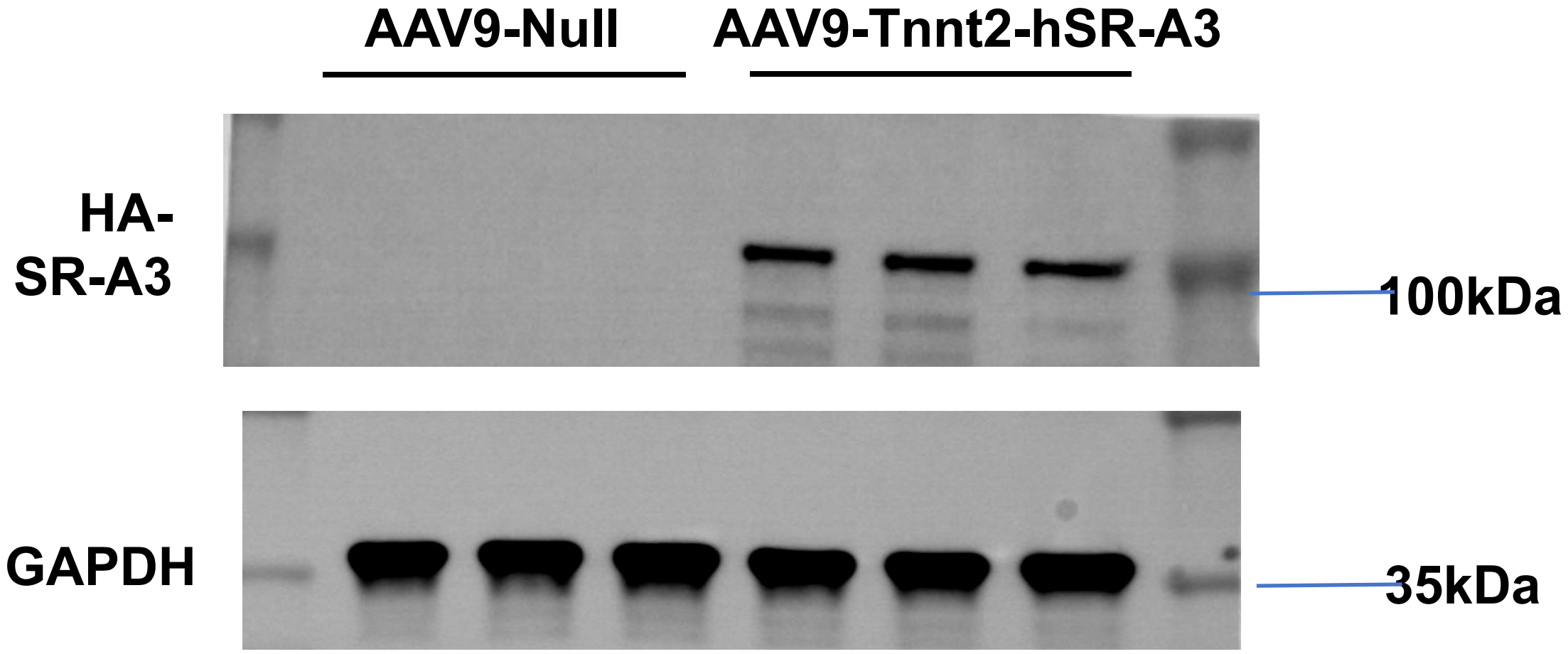

Fig S5b

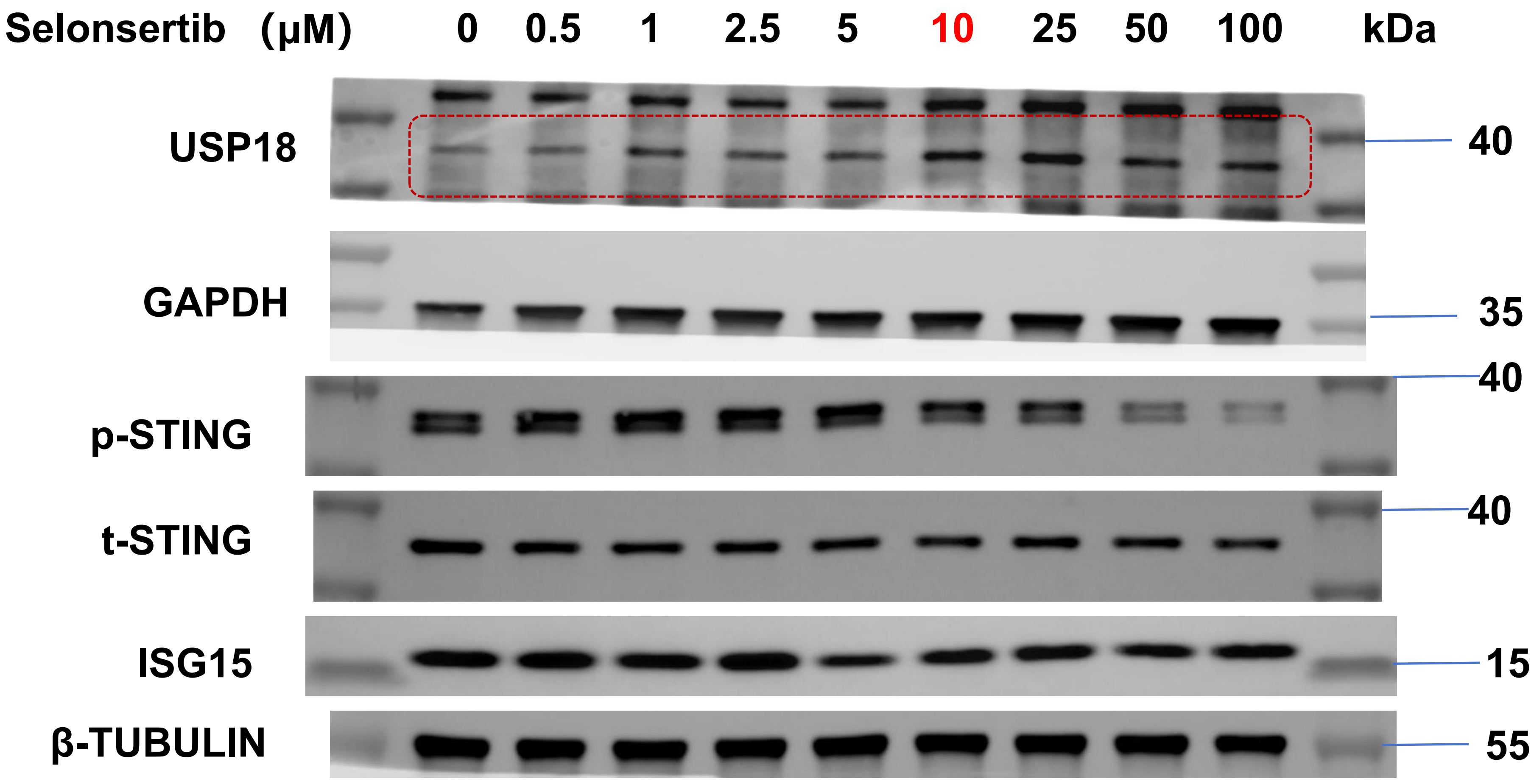

Selonsertib (μM)
0
0.5
1
2.5
5
10
25
50
100
kDa
USP18
40
GAPDH
35
p-STING
40
t-STING
40
ISG15
15
β-TUBULIN
55

Fig S5c

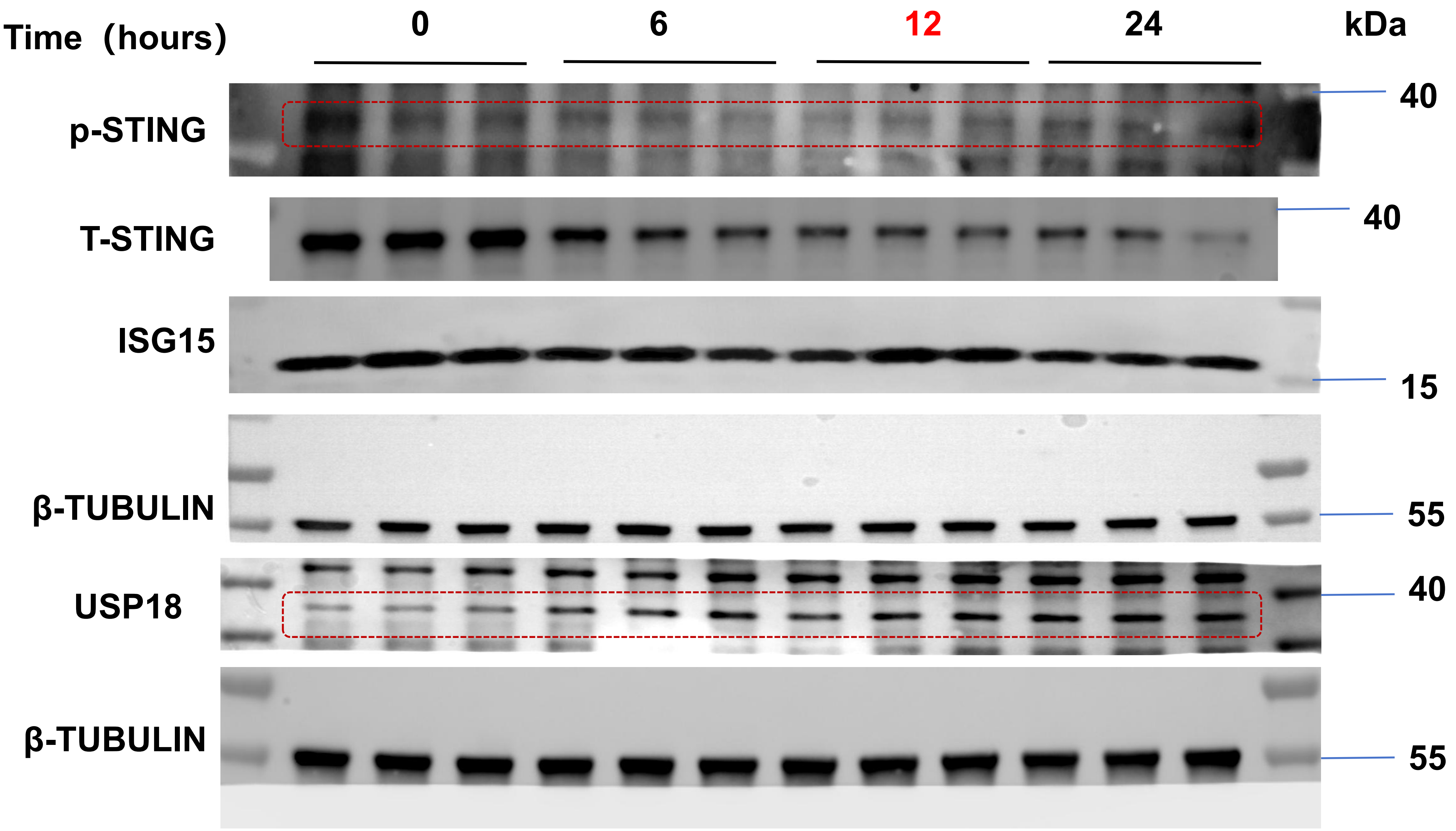

Time (hours)
0
6
12
24
kDa
p-STING
40
T-STING
40
ISG15
15
β-TUBULIN
55
USP18
40
β-TUBULIN
55
