# Supplementary material for: SR‐A3 Promotes USP18‐Mediated deISGylation of STING to Protect Against Myocardial Ischemia‐Reperfusion Injury
Source: Adv Sci (Weinh). 2026 Aug 17;13(62):e77196. doi: 10.1002/advs.77196 (PMC13479855; doi:10.1002/advs.77196)
Supplement: Supplementary file 2 [file ADVS-13-e77196-s002.docx]

**[Supplemental Material](https://www.ahajournals.org/doi/suppl/10.1161/CIRCULATIONAHA.124.069737)**

**Enzyme-Linked Immunosorbent Assay**

Cytokine concentrations in plasma were measured using ELISA kits for mouse IL-1β (RUIXIN BIOTECH, RXW203063M) and IL-6 (RUIXIN BIOTECH, RXW203049M) according to the manufacturer’s instructions.

**Echocardiography**

Cardiac function was assessed using a Vinno 6VET echocardiography system (Vinno Technology Co., Ltd, China). Mice were anesthetized with 3% isoflurane (Baxter Healthcare Corp, USA) and placed supine on a warm pad. Anesthesia was maintained with 1.0–1.5% isoflurane to stabilize heart rate at 400–500 beats per minute. Two-dimensional parasternal long-axis and short-axis views at the papillary muscle level were obtained. M-mode tracings were used to measure left ventricular posterior wall thickness in diastole (LVPWd) and systole (LVPWs), left ventricular end-diastolic volume (LVEDV), and end-systolic volume (LVESV). Ejection fraction (EF) and fractional shortening (FS) were calculated from these parameters.

**Generation of Cardiac-Specific *SR-A3* Overexpression and Knockout Mice**

Cardiac-specific *SR-A3* overexpression was achieved using an adeno-associated virus serotype 9 (AAV9) vector carrying human *SR-A3* (NM_016240.3) under the control of the cardiomyocyte-specific troponin T (*Tnnt2*) promoter (AAV9-*Tnnt2-hSR-A3*, Genomeditech). AAV-null was used as control. For knockdown, AAV9 carrying shRNA targeting *Sr-a3* (AAV9-*shSr-a3*, PackGene) was used. Six-week-old male mice received intravenous tail-vein injections of 2×10^11 vector genomes of the respective AAV9 vectors. Two weeks post-injection, mice underwent I/R surgery, echocardiography, and tissue collection.

**Cell Culture**

Neonatal rat cardiomyocytes (NRCMs) were isolated from 2-day-old Sprague-Dawley rats as previously described^1^. Hearts were dissected and subjected to sequential enzymatic digestion cycles (6 minutes each) in buffer containing 0.6 mg/mL Collagenase II (Worthington, LS004176) and 1 mg/mL pancreatin (Solarbio, T8150). NRCMs were cultured in DMEM with 10% FBS (TransGen, FS301-02). For hypoxia/reoxygenation (H/R), cells were maintained in DMEM/10% FBS for 48–72 hours, then switched to serum-free RPMI 1640 (Gibco, 11879020) and placed in a sealed chamber with 95% N₂/5% CO₂ at 37°C for 4 hours (O₂ < 0.1%). Reoxygenation was performed by replacing medium with DMEM and incubating in a 37°C/5% CO₂ incubator for 1 hour.

After H/R, cell viability was assessed using CCK-8 (Solarbio, CA1210), and LDH release was measured with a commercial kit (Nanjing Jiancheng, A020-2-2).

**Cell Isolation from Adult Mouse Hearts**

To evaluate *Sr-a3* expression across distinct cardiac cell populations, adult mouse cardiomyocytes, cardiac fibroblasts, macrophages, and endothelial cells were isolated from mouse hearts at 24 h after MI/RI or sham operation.

Cardiomyocytes were obtained via Langendorff-based retrograde perfusion. Briefly, mice received heparin (400 USP U/kg, intraperitoneally) prior to euthanasia, and the hearts were rapidly excised and cannulated onto a Langendorff apparatus. Perfusion buffer pre-warmed to 37 °C was delivered through the aorta to flush residual blood and equilibrate the tissue, followed by recirculating perfusion with collagenase II (LS004177, Worthington) at 37 °C for 8 min to dissociate the myocardium. After enzymatic digestion, the ventricles were minced, gently triturated into a single-cell suspension in perfusion buffer supplemented with 10% fetal bovine serum, and filtered through a 100-μm cell strainer to remove undigested debris. The remaining non-cardiomyocyte fraction from the digestion was further cultured to obtain cardiac fibroblasts using differential plating as previously described.

For macrophage and endothelial cell isolation, mouse hearts were perfused with normal saline to remove residual blood, and the left ventricle was collected and minced into small pieces. Tissue fragments were digested in 2 mL of single-cell digestion buffer containing collagenase I (1 mg/mL), hyaluronidase (0.2 mg/mL), and DNase I (60 U/mL) in DMEM at 37 °C for 1 h with intermittent agitation to obtain a single-cell suspension. Digestion was terminated by adding 6 mL of HBSS supplemented with 2% FBS and 0.2% BSA. The cell suspension was sequentially filtered through a 40-μm cell strainer, centrifuged at 800 × g for 5 min, and subjected to erythrocyte lysis using ACK lysis buffer for 10 min at room temperature. After neutralization with 9 mL of HBSS and centrifugation at 900 × g for 5 min, cells were resuspended in MACS buffer (PBS containing 2% FBS and 2 mM EDTA). Cardiac macrophages and endothelial cells were then positively selected using anti-F4/80 microbeads (Starter Biotec, S0K0012) and anti-CD31 microbeads (Miltenyi Biotec, 130-097-418), respectively, following the manufacturer's instructions.

**Human Embryonic Stem Cell–Derived Cardiomyocyte Differentiation**

H9 human embryonic stem cells (hESCs) were differentiated into cardiomyocytes using a defined, xeno-free, small-molecule protocol. hESCs were maintained in PSCeasy medium (Cellapy, CA1014500) until 80–90% confluent, then differentiated using the CardioEasy cardiac differentiation kit (Cellapy, CA2004500). Purification was performed with the CardioEasy purification kit (Cellapy, CA2005100), and cells were maintained in RPMI 1640 (Corning, 10-040-CVR) supplemented with B-27 (Thermo Fisher, 17504044). H/R was induced as described for NRCMs.

**HEK 293T Cell Culture and Plasmid Transfection**

HEK 293T cells were cultured in DMEM with 10% FBS. Transfection was performed using Lipofectamine™ 3000 (Thermo Fisher, L3000001). At ~70% confluence, two mixtures were prepared: (1) 30 μL Lipofectamine™ 3000 in 1.5 mL Opti-MEM, and (2) 20 μL P3000 reagent plus 10 μg plasmid (or 5 μg each of two plasmids) in 1.5 mL Opti-MEM. After 5 minutes at RT, the mixtures were combined, incubated 20 minutes at RT, and added to cells in a 10 cm dish. After 6 hours, medium was replaced with DMEM containing serum. Cells were harvested 48 hours post-transfection. The coding sequences of human SR-A3 (NM_016240.3) and USP18 (NC_000022.11) were PCR-amplified and cloned into the pcDNA3.1 vector (Sangon Biotech). Cells in 12-well plates were transfected with 1 μg plasmid per well using Lipo3000 (Thermo Fisher, L3000015). Overexpression was confirmed by western blot 48 hours post-transfection.

**RNA Interference–Mediated Gene Silencing**

Control and target siRNAs (sequences in Supplemental Table 2) were transfected into NRCMs using Lipofectamine RNAiMAX (Invitrogen, 13778030). Knockdown efficiency was evaluated by western blot 48 hours after transfection.

**Immunofluorescence Staining**

For tissue sections: Frozen heart sections (7 μm) were fixed with 4% paraformaldehyde for 15 minutes at room temperature (RT), permeabilized with 0.3% Triton X-100 for 10 minutes at RT, and blocked with 10% donkey serum in PBS. Sections were incubated overnight at 4°C with primary antibodies against SR-A3 (Bioss, China, bs-17270R; 1:200) and 4-HNE (Abcam, UK, ab48506; 1:200). Normal isotype IgG (Cell Signaling Technology, USA, #3900 and #5415) served as negative controls. After three PBS washes, sections were incubated for 1 hour at 37°C in the dark with Alexa Fluor 488-, 555-, or 647-conjugated secondary antibodies (Abcam, UK, ab150062, ab150107, ab150105, ab150155). Nuclei were stained with anti-fade mounting medium containing DAPI (ZSGB-BIO, China, ZLI-9557).

For cultured cells: Cells grown on coverslips were fixed with 4% paraformaldehyde for 10 minutes, permeabilized with 0.02% Triton X-100 for 10 minutes, and blocked with 10% donkey serum. Cells were incubated overnight at 4°C with primary antibodies against USP18 (ABclonal, China, A16739; 1:200), LAMP1 (BD Biosciences, USA, 553792), and FLAG (Beyotime, China, AF519-1). Normal isotype IgG (Cell Signaling Technology, USA, #5415; 1:200) was used as a negative control. After washing, cells were incubated with Alexa Fluor-conjugated secondary antibodies (1:1000) for 1 hour at 37°C in the dark. Nuclei were counterstained with DAPI (Beyotime, China, C1005).

**Assessment of Apoptosis**

Apoptosis was evaluated using a terminal deoxynucleotidyl transferase dUTP nick-end labeling (TUNEL) assay kit (Roche, Germany, #11684817910). Nuclei were counterstained with DAPI (Beyotime, China, #C1005). Images were acquired using a confocal microscope (FV3000, Olympus, Japan).

**Reactive Oxygen Species (ROS) Detection**

Cells were washed with PBS and incubated for 30 minutes at 37°C with 10 μM DCFH-DA (Beyotime, China, S0033S) diluted in Opti-MEM. Fluorescence was captured using the BZ-X810 microscope and analyzed with ImageJ.

**WGA Staining**

Cardiac cryosections were stained with wheat germ agglutinin (WGA, Sigma, L4895; 1:100 dilution) to assess cardiomyocyte hypertrophy. Nuclei were counterstained with DAPI (Beyotime, China, C1005). For each sample, at least 10 fields and 50 cells per section were analyzed.

**Imaging and Analysis**

Fluorescence images were acquired using an integrated fluorescence microscope (BZ-X810, KEYENCE, USA) or a confocal microscope (FV3000, Olympus, Japan). Signal intensity was quantified with ImageJ software.

**Immunohistochemical Analysis**

Masson’s trichrome staining and immunohistochemistry were performed as previously described^2^. Cryosections were fixed in cold 4% paraformaldehyde for 10 minutes and treated with 3% hydrogen peroxide in PBS (0.1 M, pH 7.4) for 10 minutes to quench endogenous peroxidase. After blocking with 10% donkey serum for 30 minutes, sections were incubated overnight at 4°C with rabbit anti-SR-A3 (Bioss, China, bs-17270R; 1:200) or rabbit anti-STING (Proteintech, China, 19851-1-AP; 1:200) diluted in blocking solution. Following PBS washes, sections were incubated with HRP-conjugated secondary antibody for 1 hour at 37°C. Staining was developed using diaminobenzidine (DAB) and counterstained with hematoxylin where indicated. Slides were examined under a light microscope.

**2,3,5-Triphenyltetrazolium Chloride (TTC)–Evans Blue Dual Staining**

Twenty-four hours after I/R surgery, mice were euthanized. Following re-occlusion of the left anterior descending coronary artery, 1 mL of 1% Evans blue dye was perfused through the thoracic aorta. Hearts were excised, rinsed gently with PBS, and frozen at –80°C for 30 minutes. Each heart was sectioned transversely from the ligation site toward the apex into 1–2 mm slices. Sections were incubated with 1% TTC at 37°C for 15–20 minutes. With this dual-staining approach, non-ischemic myocardium appeared blue, the area at risk (AAR) stained brick red, and the infarcted area remained unstained (pale/white). Infarct size was quantified as the ratio of infarct volume to left ventricular volume.

**Quantitative Real-Time PCR**

Total RNA was extracted with TRIzol (TransGen Biotech, ET111-01-V2). cDNA was synthesized from 1 μg RNA and analyzed by qRT-PCR using SYBR Green mix (TransGen Biotech, AQ131-03). Primer sequences are listed in Supplemental Table 3. mRNA levels were normalized to GAPDH and expressed relative to control.

**Protein Extraction and Western Blot**

Proteins were extracted from tissues or cells using ice-cold RIPA buffer (Di-Ning, DE301-1) with protease inhibitors (Roche, 04693116001). Concentration was determined by BCA assay (Thermo Fisher, 23225). Lysates were mixed with 5× SDS loading buffer (Beyotime, P0015L), denatured at 95°C for 10 minutes, separated by SDS-PAGE, and transferred to nitrocellulose membranes.

Membranes were blocked with 5% BSA in TBST for 1 hour at RT, then incubated overnight at 4°C with the following primary antibodies: anti-SR-A3 (Bioss, bs-17270R), anti-STING (Proteintech, 19851-1-AP), anti–phospho-STING (CST, 72971), anti-TBK1 (Proteintech, 67211-1-Ig), anti–phospho-TBK1 (CST, 5483), anti-ISG15 (UpingBio, 15981-1-AP), anti-FLAG (Beyotime, AF519-1), anti–β-Tubulin (Proteintech, 10094-1-AP), anti-HA (CST, 3724), and anti-GAPDH (Proteintech, 60004-1-Ig). After TBST washes, membranes were incubated with HRP-conjugated secondary antibody (ZSGB-BIO) for 1 hour at RT. Signals were detected with ECL substrate (Yeasen, 36208ES) on an iBright system (Invitrogen, CL1500).

**ISGylation analysi**s

For immunoprecipitation, mouse heart tissues or rat primary cardiomyocytes after MI/RI or H/R treatment were lysed in lysis buffer supplemented with protease and phosphatase inhibitors. Lysates (400 μL) were incubated with 25 μL of protein A/G magnetic beads (MedChemExpress) and 5 μl of anti-STING antibody (Proteintech) at 4°C with rotation overnight. The immunoprecipitates were washed three times with 1 mL of lysis buffer, and bound proteins were eluted by boiling in SDS sample buffer containing reducing reagent at 95°C for 5 min. The eluates and equal amounts of total cell lysates (Input) were subjected to SDS-PAGE and immunoblotted with the indicated antibodies. To avoid interference from immunoglobulin heavy chains, an HRP-conjugated mouse anti-rabbit IgG heavy-chain-specific secondary antibody (Abmart) was used for immunoprecipitation-derived blots.

**Analyses of STING dimerization**

To analyze STING dimerization, mouse heart tissues were collected at 24 h after MI/RI and lysed in mild lysis buffer (50 mM Tris-HCl [pH 8.0], 150 mM NaCl, 0.8% NP-40, 10% glycerol, protease and phosphatase inhibitors) on ice for 30 min. Tissue lysates were mixed with SDS sample buffer without reducing reagents, and samples were not boiled before electrophoresis. Proteins were separated by SDS-PAGE and immunoblotted with anti-STING antibody to detect monomeric and disulfide‑linked dimeric forms of STING.

**GST pull-down assays**

GST, GST-SR-A3 and His-USP18 constructs were used in this research. These constructs were expressed in Escherichia coli strain BL21 (Tiangen Biotechnology, Beijing, China) and GST fusion proteins were purified using glutathione Sepharose 4B beads (Pharmacia Biotech; Pfizer, New York, NY, USA).

For GST pull-down assays, lysates containing GST alone (negative control) or GST-SR-A3 were incubated with glutathione Sepharose 4B beads separately at 4 °C for 1 h with rocking. The beads were then washed three times with TEN buffer (20 mM Tris-HCl, pH 7.4, 0.1 mM EDTA, and 100 mM NaCl). Lysate containing His-USP18 was then added to each batch of beads and incubated at 4 °C overnight with rocking. The beads were then washed three times with TENT buffer (0.5% NP40, 20 mM Tris-HCl, pH 7.4, 0.1 mM EDTA, and 300 mM NaCl), centrifuged at 3000 × g for 1 min, and resuspended in 2× SDS loading buffer. The solutions were then boiled for 5 min at 100 °C and centrifuged at 12,000 × g for 1 min. Finally, the supernatants were analyzed via western blotting.

**Coimmunoprecipitation (Co-IP) Assay**

Cells were lysed in RIPA buffer with protease inhibitors. Cleared lysates were incubated overnight at 4°C with anti-FLAG (Beyotime, AF519-1), anti-STING (Proteintech, 19851-1-AP), or control IgG (ABclonal, AC005) together with protein A/G agarose beads (Solarbio, R8281). Beads were washed three times with RIPA buffer, and bound proteins were eluted by boiling in 2× SDS loading buffer for 10 minutes. Eluates were analyzed by SDS-PAGE.

**Molecular Docking**

Molecular docking of SR-A3 and USP18 was performed using the HDOCK server (http://hdock.phys.hust.edu.cn/). The crystal structure of USP18 (PDB: 5CHT) was obtained from the RCSB Protein Data Bank (http://www.rcsb.org), and the SR-A3 model (AF-Q6AZY7-F1-v6) was retrieved from the AlphaFold Protein Structure Database (https://alphafold.ebi.ac.uk/). Rigid protein–protein docking was conducted, and resulting complexes were visualized and analyzed in PyMOL (Version 2.4).

**Virtual Screening of USP18 Agonists via Molecular Docking**

The three-dimensional crystal structure of USP18 was obtained from the Protein Data Bank (PDB). Molecular dynamics (MD) simulations were employed to optimize the energy of the protein structure, thereby enhancing the conformational stability and biological relevance of the target. Subsequently, the DoGSiteScorer tool was used to identify and evaluate potential ligand-binding sites on the protein surface. High-scoring pockets were selected as docking regions and further refined through geometric and energetic optimization.

Using the optimized active site as the target, virtual screening of small-molecule compounds from the DrugBank database was performed with the Schrödinger software suite. During the docking process, key parameters such as binding energy, docking score, number of hydrogen bonds, and hydrophobic interactions between each candidate compound and USP18 were recorded. Based on the docking results, compounds forming stable interactions with critical residues were identified. By comparing binding energies, docking scores, and binding modes, potential USP18 agonists with favorable interaction profiles were selected as candidates for subsequent experimental validation.

**RNA-Seq Data Analysis**

Total RNA was extracted from hearts of *WT* and *Sr-a3*^–/–^ mice after I/R. RNA sequencing analysis of heart tissues was performed by Shanghai Majorbio Bio-Pharm Technology Co., Ltd. (Shanghai, China). All RNA sequencing data were analyzed using the free online platform of Majorbio Cloud Platform (www.majorbio.com). Differentially expressed genes (DEGs) were identified based on the criteria of Padjust <0.05 and |log2FC| > 1.

**References**

1.	Zhang L, Miao G, Mei S, Han Y, Xu Y, Zhang W, Chen J, Lu K, Zhao Y, Zhou Z, Chen J, Guo J, Lai P, Lam SM, Shui G, Zhang L, Zhang W, Huang W, Wang Y and Xian X. KIF13B Attenuates Sepsis-Induced Myocardial Dysfunction through the Stabilization of PLIN5. *Research (Wash D C)*. 2026;9:1033.

2.	Wang H, Huang X, Xu P, Liu X, Zhou Z, Wang F, Li J, Wang Y, Xian X, Liu G and Huang W. Apolipoprotein C3 aggravates diabetic nephropathy in type 1 diabetes by activating the renal TLR2/NF-kappaB pathway. *Metabolism*. 2021;119:154740.

**Supplemental Figures and legends:**

**Figure. S1. Generation of *Sr-a3* knockout mouse model using CRISPR/Cas9.**
**(a)** Schematic of the CRISPR/Cas9 strategy targeting exons 2-6 of the mouse *Sr-a3* gene.
**(b)** Genotyping results for wild-type (*WT*), heterozygous (*Sr-a3*^+/−^), and homozygous (*Sr-a3*^−/−^) mice.
**(c)** QPCR analysis of *Sr-a3* mRNA levels in heart tissues of *WT* and *Sr-a3^-/-^* mice (n = 6 per group).

Data are presented as the mean ± SEM and were analyzed by two-tailed unpaired Student's t-test.

**Figure. S2. Global knockout or overexpression of *Sr-a3* modulates long-term myocardial remodeling and heart failure after MI/RI.**
**(a)** Echocardiographic parameters of *WT* and *Sr-a3^-/-^* mice 4 weeks after MI/R injury (n = 6 per group).
**(b)** Gross cardiac morphology of *WT* and *Sr-a3^-/-^* mice 4 weeks after MI/R.
**(c)** Heart weight/body weight (HW/BW) and heart weight/tibia length (HW/TL) ratios for the mice in (**a**) (n = 6 per group).
**(d)** Representative images of wheat germ agglutinin (WGA; scale bar: 50 μm), TUNEL, and 4-HNE staining in heart sections from the mice in (A) (scale bar: 100 μm).
**(e)** Quantification of cardiomyocyte cross-sectional area (from WGA, n=50 cells), and TUNEL- and 4-HNE-positive areas from (**d**).
**(f)** Echocardiographic parameters of mice infected with AAV9*-Null* or AAV9*-hSR-A3* 4 weeks after MI/R (n=6 per group).
**(g)** Gross cardiac morphology of the mice in (**f**).
**(h)** HW/BW and HW/TL ratios for the mice in (**f**) (n = 6 per group).
**(i)** Representative images of WGA, TUNEL, and 4-HNE staining in heart sections from the mice in (**f**) (scale bars: 50 μm for WGA, 100 μm for others).
**(j)** Quantification of the data shown in (**i**).

Data are presented as the mean ± SEM and were analyzed by two-tailed unpaired Student's t-test or two-way ANOVA with Tukey post hoc tests.

**Figure. S3. Validation of Sr‑a3 knockdown and overexpression in NRCMs in vitro and in mouse hearts in vivo.**
**(a)** QPCR analysis of *Sr-a3* mRNA levels in NRCMs transfected with *scramble* or *siSr-a3* mix (n = 3, independent biological replicates).
**(b)** Western blot analysis of SR-A3 protein expression in NRCMs transfected with *scramble* or *siSr-a3-*1 or *siSr-a3-*2 or *siSr-a3-*3.
**(c)** LDH release in the culture medium of NRCMs transfected as indicated, with or without H/R (n = 3 per group, independent biological replicates).
**(d)** Cell viability assessed by CCK-8 assay in NRCMs under the conditions described in (**c**).
**(e)** QPCR analysis of *SR-A3* expression in NRCMs transfected with empty vector or FLAG-hSR-A3 (n = 3 independent biological replicates).
**(f)** Western blot analysis of SR-A3 protein expression in NRCMs from (**e**).
**(g)** LDH release in the culture medium of NRCMs from (**e**), with or without H/R (n = 3 per group, independent biological replicates).
**(h)** Cell viability (CCK-8) of NRCMs from (**e**) under the conditions described in (**g**).

**(i)** qPCR analysis of cardiac Sr-a3 mRNA levels in mice injected with AAV9-*Null* or cardiomyocyte-targeted AAV9-*Tnnt2-shSr-a3*.
**(j)** Western blot analysis of cardiac SR-A3 protein expression in mice injected with AAV9-*Null* or AAV9-*Tnnt2-shSr-a3*.
**(k)** qPCR analysis of cardiac Sr-a3 mRNA levels in mice injected with AAV9-*Null* or cardiomyocyte-targeted AAV9-*Tnnt2-hSR-A3*.
**(l)** Western blot analysis of SR-A3-HA protein expression in cardiac tissues from mice injected with AAV9-*Null* or AAV9-*Tnnt2-hSR-A3*.
**(m)** Representative immunofluorescence images showing SR-A3 expression and its colocalization with the cardiomyocyte marker cardiac troponin T (CTNT) in cardiac tissues from mice injected with AAV9*-Tnnt2-hSR-A3*. DAPI was used to stain nuclei, VIMENTIN was used to label non-cardiomyocyte mesenchymal cells, and negative control staining was included to assess background signal.

Data are presented as the mean ± SEM and were analyzed by two-tailed unpaired Student's t-test or two-way ANOVA with Tukey post hoc tests.

**Figure. S4. Cardiac-specific modulation of *Sr-a3* affects long-term myocardial remodeling after MI/RI.**
**(a)** Echocardiographic parameters of mice infected with AAV9*-Null* or AAV9*-Tnnt2-shSr-a3* (cardiac-specific knockdown) 4 weeks after MI/R (n = 6 per group).
**(b)** Gross cardiac morphology of the mice in (**a**), and HW/BW and HW/TL ratios for the mice in (**a**) (n = 6 per group).
**(c)** Representative images of WGA, TUNEL, and 4-HNE staining in heart sections from the mice in (**a**) (scale bars: 50 μm for WGA, 100 μm for others).
**(d)** Quantification of the data shown in (**c**).
**(e)** Echocardiographic parameters of mice infected with AAV9*- Null* or AAV9*-Tnnt2-hSR-A3* (cardiac-specific overexpression) 4 weeks after MI/R (n = 6 per group).
**(f)** Gross cardiac morphology of the mice in (**e**) , and HW/BW and HW/TL ratios for the mice in (**e**) (n = 6 per group).
**(g)** Representative images of WGA, TUNEL, and 4-HNE staining in heart sections from the mice in (**f**).
**(h)** Quantification of the data shown in (**g**).

Data are presented as the mean ± SEM and were analyzed by two-tailed unpaired Student's t-test or two-way ANOVA with Tukey post hoc tests.

**Figure. S5. Determination of optimal Selonsertib treatment conditions.**
**(a)** Cell viability (CCK-8 assay) of NRCMs treated with increasing concentrations of Selonsertib.
**(b)** Western blot analysis of USP18, p-STING, STING, and ISG15 in NRCMs treated with different concentrations of Selonsertib.
**(c)** Western blot analysis of USP18, p-STING, STING,and ISG15 in NRCMs treated with Selonsertib for different durations.
**(d)** Quantification of the data shown in (**c**).

Data are presented as the mean ± SEM and were analyzed by one-way ANOVA with Tukey post hoc tests.

**Supplemental Table 1.** Clinical characteristics of all enrolled participants

| Variable | MI post-PCI (n=10) | Donor (n=6) | P value | Test method |
| --- | --- | --- | --- | --- |
| Age, y | 62.50 ± 10.09 | 63.17 ± 7.57 | 0.8912 | t test |
| Body mass index, kg/m2 | 25.23 ± 3.73 | 23.97 ± 1.49 | 0.4465 | t test |
| Gender （male 1 female 0） | 6 (60.0%) | 4 (66.7%) | 1 | Fisher exact test |
| Smoking history | 4 (40.0%) | 2 (33.3%) | 1 | Fisher exact test |
| Hypertension | 5 (50.0%) | 2 (33.3%) | 0.6329 | Fisher exact test |
| Type 2 Diabetes | 3 (30.0%) | 1 (16.7%) | 1 | Fisher exact test |
| Hypercholesterolemia | 8 (80.0%) | 1 (16.7%) | 0.035 | Fisher exact test |
| Previous myocardial infarction | 5 (50.0%) | 0 (0.0%) | 0.0934 | Fisher exact test |
| Prior stroke/ transient ischemic attack | 1 (10.0%) | 0 (0.0%) | 1 | Fisher exact test |
| Chronic obstructive pulmonary disease | 1 (10.0%) | 0 (0.0%) | 1 | Fisher exact test |
| Renal insufficiency | 1 (10.0%) | 1 (16.7%) | 1 | Fisher exact test |

## Supplemental Table 2. The sequences of the siRNAs used in this experiment

| **Gene** | **Direction** | **Sequences 5'-3’** |
| --- | --- | --- |
| *Sr-a3-1* | Forward | GUGCUCGGUUAGACUUCAA |
|  | Reverse | UUGAAGUCUAACCGAGCAC |
| *Sr-a3-2* | Forward | GAGCUCUACUACCUGAAUA |
|  | Reverse | UAUUCAGGUAGUAGAGCUC |
| *Sr-a3-3* | Forward | GAAGCUUGUGUCAAUGCAA |
|  | Reverse | UUGCAUUGACACAAGCUUC |
| *Isg15* | Forward | CCAAAGCGGUGAAAUGCUA |
|  | Reverse | UAGCAUUUCACCGCUUUGG |
| *Usp18* | Forward | AUAGGUACACUGGACAUCC |
|  | Reverse | GGAUGUCCAGUGUACCUAU |

**Supplemental Table 3.** The sequences of the qPCR primers used in this experiment

| **Gene** | **Species** | **Direction** | **Sequences 5'-3’** |
| --- | --- | --- | --- |
| *Sr-a3* | Mouse | Forward | CAATGTGGTCACGTTTCCTAGC |
|  |  | Reverse | CTTGGACTCTCCTTTATCCAGGG |
| *IL-1β* | Mouse | Forward | AGGCTCCGAGATGAACAA |
|  |  | Reverse | AAGGCATTAGAAACAGTCC |
| *IL-6* | Mouse | Forward | TTCTTGGGACTGATGCTG |
|  |  | Reverse | CTGGCTTTGTCTTTCTTGTT |
| *NOX2* | Mouse | Forward | ACTCCTTGGGTCAGCACTGG |
|  |  | Reverse | GTTCCTGTCCAGTTGTCTTCG |
| *Nrf2* | Mouse | Forward | GCCCACATTCCCAAACAAGAT |
|  |  | Reverse | CCAGAGAGCTATTGAGGGACTG |
| *GAPDH* | Mouse | Forward | TGGATTTGGACGCATTGGTC |
|  |  | Reverse | TTTGCACTGGTACGTGTTGAT |
| *Ifn-β* | Mouse | Forward | AACTCCACCAGCAGACAGTG |
|  |  | Reverse | CATCCAGGCGTAGCTGTTGT |
| *Tnf-α* | Mouse | Forward | CAACCTCCTCTCTGCCATCAAGA |
|  |  | Reverse | CTGGAAGACCCCTCCCAGATAGA |
| *MCP-1* | Mouse | Forward | CCACTCACCTGCTGCTACTCAT |
|  |  | Reverse | CACTGTCACACTGGTCACTCCT |
| *Sr-a3* | Rat | Forward | AGGTCATGGGCCTACAGCTA |
|  |  | Reverse | TGTGCAAGACAGCCGTACAT |
| *GAPDH* | Rat | Forward | TGATGACATCAAGAAGGTGGTGAAG |
|  |  | Reverse | TCCTTGGAGGCCATGTAGGCCAT |
| *SR-A3* | Human | Forward | TGCATGGATACTGACCCTGA |
|  |  | Reverse | GCCGTGTTACCAGCTTCTTC |
| *GAPDH* | Human | Forward | CGGAGTCAACGGATTTGGTCGTAT |
|  |  | Reverse | AGCCTTCTCCATGGTGGTGAAGAC |
